# LC-MS/MS metabolomics analysis: Leaf harvesting plus pesticide spraying can further improve the growth quality of *Kandelia obovata* Sheue & al.

**DOI:** 10.3389/fpls.2025.1573160

**Published:** 2025-09-10

**Authors:** Ziyu Zhao, Sheng Yang, Qiuxia Chen

**Affiliations:** ^1^ Wenzhou Key Laboratory of Resource Plant Innovation and Utilization, Zhejiang Institute of Subtropical Crops, Zhejiang Academy of Agricultural Sciences, Wenzhou, China; ^2^ College of Forestry and Biotechnology, Zhejiang Agricultural and Forestry University, Hangzhou, China

**Keywords:** *Kandelia obovata*, spraying, leaf picking, LC-MS/MS, untargeted metabolomics analysis

## Abstract

This study aims to reveal the changes in the growth of new leaves and soil nutrient capacity of *Kandelia candel* under different pest control treatments, and to explore the effects of each treatment on the metabolome of new leaves and soil of *Kandelia candel* by non-targeted metabolomics. Four treatments were set up in Yanpu Bay, Wenzhou, Zhejiang from June to October 2023: CK (T1), Leaf picking (T2), Spraying (T3), Leaf picking plus Spraying (T4). The experimental results showed that all three treatments promoted the germination of new branches, the increase in the number of leaves and the improvement of plant quality to varying degrees, among which T4 had the most significant effect. LC-MS/MS analysis showed that the metabolites of soil and new leaves changed significantly under the four treatment conditions, mainly involving the formation of lipids and lipid molecules, organic acids and their derivatives in the soil, and lipids and lipid molecules, phenylpropanoids and polyketides in the new leaves. 528 metabolites in the soil and 1174 metabolites in the new leaves were identified. This study revealed the differences in metabolites of soil and new leaves under different pest control treatments by combining Spearman analysis and KEGG database. The results showed that primary and secondary metabolites in the soil, such as lipids, lipid-like molecules, and organic acids and their derivatives, may play an important role in pest resistance, especially the biosynthesis of the linoleic acid pathway and secondary bile acid biosynthesis, which had the highest enrichment. Similarly, primary and secondary metabolites in new leaves, such as lipids, lipid-like molecules, phenylpropanoids, and polyketides, may also play an important role in pest resistance, with the highest biosynthesis enrichment of flavonoid and flavonol biosynthesis and flavonoid biosynthesis. These findings provide a new theoretical basis for the metabolic mechanism of pest control in *Kandelia candel*, and provide new ideas and useful metabolites for genetic improvement of mangrove plant resistance using molecular breeding technology.

## Introduction

Mangrove forests are unique woody plant communities that grow in the intertidal zones of tropical and subtropical regions, playing a critical role in maintaining the ecological balance of coastal ecosystems (Duke et al., 2016; Duke et al., 2020; Nagelkerken et al., 2021). Among the various species of mangroves, *Kandelia obovata* is the most widely distributed in China. Due to its superior cold resistance, it has become one of the dominant species in the northern regions of the country (Huang et al., 2023; Xu et al., 2023). However, in recent years, the northern mangrove regions of Zhejiang Province have been increasingly affected by pest infestations, exacerbated by the limited number of species available in these areas. Notably, the armored scale insect (*Pseudaulacaspis cockerelli*) has emerged as a major pest, causing significant damage to *Kandelia obovata* populations (Xu Y. et al., 2021; Liu et al., 2023). Research has shown that various mangrove species, such as *Rhizophora mangle*, *Avicennia marina*, and *Bruguiera gymnorrhiza*, respond to pest attacks by increasing the concentration of secondary metabolites, including tannins, quercetin, kaempferol, ferulic acid, caffeic acid, gallic acid, flavonoids, citral, menthol, β-caryophyllene, pine oil, quinoline, isoquinoline, and indole-3-acetic acid (IAA) (Duarte et al., 2020; Kumar et al., 2020; Gao et al., 2021; Chen et al., 2022). These metabolites enhance the plants’ chemical defenses, improving their resistance to pests, boosting antioxidant capacity, and strengthening their stress tolerance. This, in turn, inhibits pest growth and survival, which significantly increases the plants’ survival rates and competitive advantages. Pest infestations lead to substantial metabolic shifts in mangrove plants, affecting physiological parameters, metabolites, and hormone levels, ultimately influencing plant growth, reproduction, and resilience to environmental stresses (Ravi et al., 2020). Specifically, pest damage has been shown to alter sugar and amino acid metabolism in mangrove leaves, while also affecting hormone regulation and stress response mechanisms. The application of chemical pesticides, such as thiacloprid and imidacloprid, has been demonstrated to reduce pest damage to mangrove plants, often accompanied by the upregulation of defensive metabolites and changes in bioactive components, potentially enhancing pest resistance (Gajger and Dar, 2021). However, the use of abamectin has been associated with a decrease in chlorophyll content, stomatal density, and an increase in water and bicarbonate levels, suggesting that it may negatively affect photosynthetic efficiency and water balance in the plants (Devillers, 2020; Lu et al., 2020).

Metabolomics, a key area within omics research, is particularly useful for detecting subtle changes in gene expression and protein activity through the analysis of metabolites (Wang et al., 2006). By identifying minor variations between samples, metabolomics has become an essential tool in plant pest research, particularly for studying changes in nutritional content and pest resistance in mangrove species. Untargeted metabolomics, which analyzes the total metabolite content of biological samples, allows for a detailed characterization of molecular phenotypes and comparison of metabolite profiles across different sample groups (Muñoz-Redondo et al., 2021). High-throughput metabolomics methods have recently gained prominence due to their ability to provide comprehensive metabolite profiles of biological samples (Das et al., 2022). Techniques such as GC-MS-based metabolomics, which focus on compounds involved in primary metabolism, and newer approaches such as UPLC-TOF-MS, which offer high sensitivity, accuracy, and time efficiency, enable more precise measurements of metabolites at low concentrations (Tenuta et al., 2020). The application of untargeted metabolomics in pest studies has provided insights into the metabolic responses of plants such as rice, tomato, *Arabidopsis thaliana*, and grape to pest infestations. These responses include the accumulation of secondary metabolites (e.g., phenolic acids, flavonoids, alkaloids, and glucosinolates), activation of plant hormone signaling pathways (e.g., salicylic acid and jasmonic acid), and changes in primary metabolites (e.g., sugars and organic acids). These metabolic adjustments enhance the plant’s direct defense mechanisms and its ability to attract natural predators, offering valuable insights into pest resistance mechanisms and integrated pest management strategies (Yactayo-Chang et al., 2020; Naskar et al., 2021; Li et al., 2024).

To enhance the growth and quality of *Kandelia obovata* in the coastal mangrove region of Wen Zhou, Zhejiang, this study employed a combination of pest control measures, including chemical spraying and leaf removal. A pesticide spraying trial was conducted using a mixture of abamectin, thiamethoxam, and imidacloprid at a 3:50:100 ratio. Recent studies have indicated that modern insecticides such as thiamethoxam, imidacloprid, chlorpyrifos, indoxacarb, and abamectin benzoate are relatively safe for beneficial predatory hemipterans and armored scale insects (Elzen, 2001). Foliar application of imidacloprid has been shown to effectively control *Cycads* scale insects without damaging plant tissues, making it effective against light to moderate infestations (Emshousen et al., 2004). Moreover, chemicals like glyphosate, bitter orange alkaloids, and thiamethoxam have demonstrated effective control over *Kandelia obovata*’s oyster scale insect (Ghanim and Elgohary, 2015). Chemical pesticide treatments induce the production of secondary metabolites in plants, which act as a defensive response to pesticide stress. For example, under thiamethoxam treatment, polyphenol and flavonoid levels in soybean seedlings were significantly higher than in the control group (Dhungana et al., 2016). Abamectin benzoate, a widely used insecticide and acaricide, is considered safe for humans, livestock, crops, and most beneficial arthropods at recommended doses, and is biodegradable (Wang et al., 2015). It is extensively used for pest control in grains, economic crops, and vegetables. Chlorpyrifos has proven effective against the white shield scale of oil camellia, and abamectin also exhibits efficacy in controlling this pest. Using a combination of abamectin and chlorpyrifos in rotation has been proposed to delay the onset of pesticide resistance (Hu et al., 2018). Metabolites, the final products of gene transcription and protein expression, form the material basis of plant phenotypes under the influence of both internal and external factors. Consequently, chemical pesticides indirectly affect plant secondary metabolism, which in turn influences their biological activity. Secondary metabolism and metabolite accumulation are regulated by the plant itself, as well as by various biotic and abiotic factors. Through advanced metabolomics research, a deeper understanding of the plant-environment relationship can be achieved, elucidating the functional roles of plant genes and providing a theoretical foundation for future research in metabolomics and functional genomics (Jay et al., 2019; Fredy et al., 2021). Despite the growing body of research, no studies have yet explored the effects of pesticide spraying on *Kandelia obovata* leaves and soil using LC-MS-based untargeted metabolomics.

The present study aims to investigate the pest and disease issues affecting mangroves in southern Zhejiang. Using *Kandelia obovata* along the Pu Wan Bay as an experimental model, a short-term field trial was conducted in the intertidal zone. The experimental treatments were as follows: T2 (leaf removal group), T3 (pesticide spraying group), T4 (leaf removal + pesticide spraying group), and T1 (control group). The effects of these treatments on *Kandelia obovata* growth, new shoots, leaf quality, and soil physicochemical properties were assessed. LC-MS was employed to qualitatively and quantitatively analyze metabolites in newly grown leaves (L1, L2, L3, L4) and soil (S1, S2, S3, S4) under the four treatments, followed by comparative analysis. The metabolic products and pathways under these conditions were identified to clarify the metabolic phenotypes and strategies employed by *Kandelia obovata* in response to pesticide treatments. This research will provide a foundation for breeding pest-resistant varieties and advancing pest management technologies.

This study proposes two hypotheses: (1) Treatment S4 will improve the soil physicochemical properties of pest-damaged *Kandelia obovata*, correlating with linoleic acid and sphingolipid levels; (2) Treatment L3 will promote the quality of new leaves and shoots in pest-damaged *Kandelia obovata*, with vegetation background indicators correlating with quinic acid and choline parameters. Testing these hypotheses will yield valuable insights into the adaptive strategies of pest-damaged *Kandelia obovata* under pesticide and leaf removal treatments. Ultimately, this study seeks to elucidate the interactions between leaf metabolites and plant growth indicators, as well as the relationship between soil properties and soil metabolites, providing practical solutions for pest control in artificial mangrove forests in northern China.

## Materials and methods

### The overview of the experimental site

**Figure 1:**
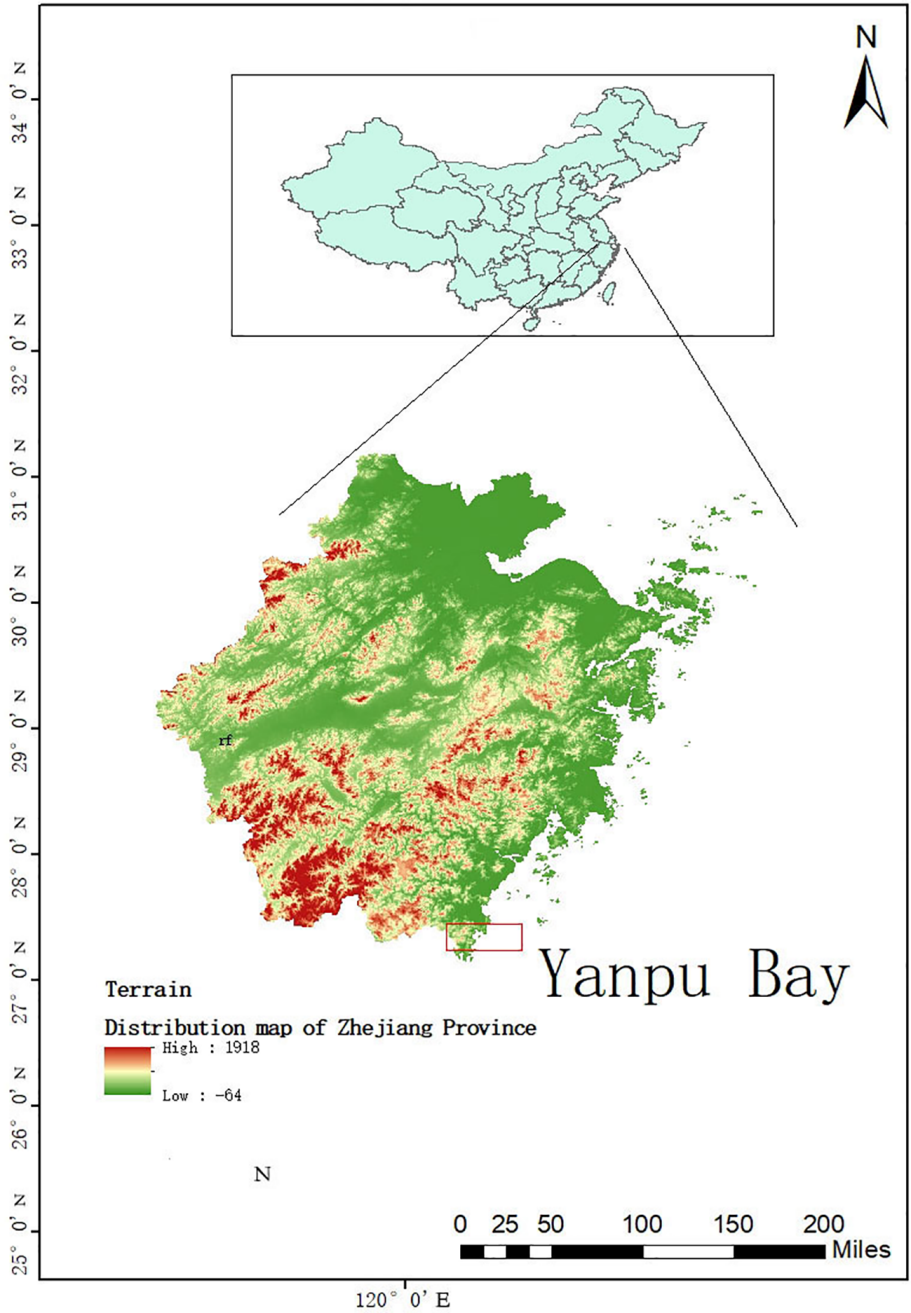
Geographical location of the sampling sites in Yanpu Bay.

The experimental site is located in the Yanpu Bay of Cangnan County, Wenzhou City, Zhejiang Province (27°30′N, 120°23′E), at an elevation of 300 meters above sea level, characterized by flat terrain (**Figure 1**). This area is classified as having a subtropical monsoon climate, with an annual average temperature ranging from 14°C to 18°C, and an annual precipitation of 1753.9 mm, indicating abundant rainfall throughout the year. The soil in the experimental area is saline-alkali soil. Prior to the experiment, the surface soil (10–20 cm depth) exhibited a pH of 7.8, an organic matter content of 10.72 g·kg^-^¹, total nitrogen content of 1.23 g·kg^-^¹, total phosphorus content of 0.75 g·kg^-^¹, available phosphorus content of 17.19 mg·kg^-^¹, available potassium content of 1.91 g·kg^-^¹, soluble salt content of 27.75 g·kg^-^¹, available nitrogen content of 65.85 mg·kg^-^¹, ammonium nitrogen content of 335.01 mg·kg^-^¹, and nitrate nitrogen content of 69.34 mg·kg^-^¹. The soil texture was composed of clay: silt: fine sand in the proportions of 18.29%, 71.15%, and 10.56%, respectively.

### Experimental design

Four treatments were applied along the embankment in parallel: T1 (CK), the control treatment; T2, leaf removal treatment; T3, pesticide application treatment; and T4, combined leaf removal and pesticide application treatment. A completely randomized block design was used, with each plot measuring 25 m² (5 m × 5 m), and each treatment was replicated three times. Starting from June 2023, a short-term positioning experiment was conducted on the mudflat outside the facility. Leaf removal was performed in treatments T2 and T4, and pesticide application was performed in treatments T3 and T4. Pesticide was applied twice in T3 and T4, at the end of August and September 2023, respectively, to ensure that the pesticide dosage was consistent for both treatments.

### Sample collection

In October 2023, measurements of plant height, basal diameter, and canopy spread were conducted on *Kandelia obovata*. For each treatment, six randomly selected, healthy new branches were chosen. These branches were rinsed three times with sterile water, then dried, after which their length, diameter, and leaf count were recorded. Additionally, five healthy new leaves were selected from each treatment, frozen in liquid nitrogen, and stored at -80°C for subsequent plant leaf metabolomics analysis. Soil samples were also collected, divided into two portions: one portion was air-dried, sieved, and used for measuring soil chemical parameters, while the other portion was frozen in liquid nitrogen and stored at -80°C for soil metabolomics analysis.

Ammonium acetate (NH_4_AC) was purchased from Sigma Aldrich, Acetonitrile was purchased from Merck, ammonium hydroxide (NH_4_OH) and methanol were purchased from Fisher. Immediately freeze the plant leaf (80 mg of leaf or floral tissue) and soil samples in liquid nitrogen, followed by grinding them into a fine powder using a mortar and pestle. To extract metabolites, add 1000 µL of a methanol/acetonitrile/H_2_O (2:2:1, v/v/v) solution to the homogenized tissue. Centrifuge the mixture at 14,000 g for 20 minutes at 4°C. Transfer the supernatant to a vacuum concentrator for evaporation. For LC-MS analysis, re-dissolve the sample in 100 µL of a 1:1 (v/v) acetonitrile/water solvent, centrifuge at 14,000 g for 15 minutes at 4°C, and inject the supernatant into the system. To assess the stability and reproducibility of the analytical procedure, prepare quality control (QC) samples by pooling 10 µL from each sample, which are then analyzed alongside the experimental samples. QC samples are inserted and analyzed periodically after every five samples to ensure consistent performance.

Leaf morphology, including leaf area (single leaf surface area), leaf dry weight, and specific leaf area, was measured using the LA-S Plant Image Analyzer/Leaf Area Meter system software. New leaves of Autumn Olive (Elaeagnus umbellata) were oven-dried at 70°C to a constant weight, and dry weight was recorded. Soil pH was determined using a pH meter, while soil organic matter (SOM) content was measured via the potassium dichromate-heating method. Total nitrogen (TN) was quantified using the Kjeldahl method, and total phosphorus (TP) and available phosphorus (AP) were determined using the molybdenum-antimony colorimetric method. Soluble salts were measured by gravimetric analysis, available nitrogen (AN) was assessed using the diffusion method, and available potassium (AK) was measured using a flame photometer. Ammonium nitrogen (NH_4_-N) and nitrate nitrogen (NO_3_-N) were determined by colorimetric methods, and soil texture was analyzed using a laser diffraction particle size analyzer.

Analysis was performed using an UHPLC (1290 Infinity LC, Agilent Technologies) coupled to a quadrupole time-of-flight (AB Sciex TripleTOF 6600) in Shanghai Applied Protein Technology Co., Ltd. For HILIC separation, samples were analyzed using a 2.1 mm × 100 mm ACQUIY UPLC BEH Amide 1.7 µm column (waters, Ireland). In both ESI positive and negative modes, the mobile phase contained A=25 mM ammonium acetate and 25 mM ammonium hydroxide in water and B= acetonitrile. The gradient was 95% B for 0.5 min and was linearly reduced to 65% in 6.5 min, and then was reduced to 40% in 1 min and kept for 1 min, and then increased to 95% in 0.1 min, with a 3 min re-equilibration period employed. The ESI source conditions were set as follows: Ion Source Gas1 (Gas1) as 60, Ion Source Gas2 (Gas2) as 60, curtain gas (CUR) as 30, source temperature: 600°C, IonSpray Voltage Floating (ISVF) ± 5500 V. In MS only acquisition, the instrument was set to acquire over the m/z range 60-1000 Da, and the accumulation time for TOF MS scan was set at 0.20 s/spectra. In auto MS/MS acquisition, the instrument was set to acquire over the m/z range 25-1000 Da, and the accumulation time for product ion scan was set at 0.05 s/spectra. The product ion scan is acquired using information dependent acquisition (IDA) with high sensitivity mode selected. The parameters were set as follows: the collision energy (CE) was fixed at 35 V with ± 15 eV; declustering potential (DP), 60 V (+) and −60 V (−); exclude isotopes within 4 Da, candidate ions to monitor per cycle: 10. Analysis was performed using an UHPLC (Vanquish UHPLC, Thermo) coupled to a Orbitrap in Shanghai Applied Protein Technology Co., Ltd. For HILIC separation, samples were analyzed using a 2.1 mm × 100 mm ACQUIY UPLC BEH Amide 1.7 µm column (waters, Ireland). In both ESI positive and negative modes, the mobile phase contained A=25 mM ammonium acetate and 25 mM ammonium hydroxide in water and B= acetonitrile. The gradient was 98% B for 1.5 min and was linearly reduced to 2% in 10.5 min, and then kept for 2 min, and then increased to 98% in 0.1 min, with a 3 min re-equilibration period employed. The ESI source conditions were set as follows: Ion Source Gas1 (Gas1) as 60, Ion Source Gas2 (Gas2) as 60, curtain gas (CUR) as 30, source temperature: 600°C, IonSpray Voltage Floating (ISVF) ± 5500 V. In MS only acquisition, the instrument was set to acquire over the m/z range 80-1200 Da, the resolution was set at 60000 and the accumulation time was set at 100ms. In auto MS/MS acquisition, the instrument was set to acquire over the m/z range 70-1200 Da, the resolution was set at 30000 and the accumulation time was set at 50ms, exclude time within 4 s.

### Data processing

Data were organized using Excel 2019 software, and descriptive statistics were performed using IBM SPSS Statistics 26 software. One-way analysis of variance (ANOVA) and multiple comparisons were conducted using the LSD method (P < 0.05 for significance). Graphs were generated using Origin 2021 software (OriginLab Corp., USA).

The raw MS data were converted to MzXML files using ProteoWizard MSConvert before importing into freely available XCMS software. For peak picking, the following parameters were used: centWave m/z = 10 ppm, peakwidth = c (10, 60), prefilter = c (10, 100). For peak grouping, bw = 5, mzwid = 0.025, minfrac = 0.5 were used. CAMERA (Collection of Algorithms of MEtabolite pRofile Annotation) was sued for annotation of isotopes and adducts. In the extracted ion features, only the variables having more than 50% of the nonzero measurement values in at least one group were kept. Compound identification of metabolites was performed by comparing of accuracy m/z value (<10 ppm), and MS/MS spectra with an in-house database established with available authentic standards.

After sum-normalization, the processed data were analyzed by R package (ropls), where it was subjected to multivariate data analysis, including Pareto-scaled principal component analysis (PCA) and orthogonal partial least-squares discriminant analysis (OPLS-DA). The 7-fold cross-validation and response permutation testing were used to evaluate the robustness of the model. The variable importance in the projection (VIP) value of each variable in the OPLS-DA model was calculated to indicate its contribution to the classification. Student’s t test was applied to determine the significance of differences between two groups of independent samples. VIP > 1 and *P* < 0.05 were used to screen significant changed metabolites. Pearson’s correlation analysis was performed to determine the correlation between two variables. Subsequently, Fisher’s exact test was performed using the KEGG database (https://www.kegg.jp/kegg/pathway.html) to identify the biological pathways most relevant to the experimental treatments.

## Results

### Effects of different treatments on the growth of plants and new branches and leaves

As shown in **Table 1**, compared with T1 (CK), all three leaf treatments significantly increased the plant height of *Kandelia obovata*. Among these, the highest plant height (66 cm) was observed under T4 treatment, which represented a 33.1% increase (*P* < 0.05). All three treatments also improved leaf morphology, with T4 treatment resulting in significant increases in leaf area, leaf dry weight, and leaf area index by 42.4%, 82.4%, and 86.2%, respectively, compared to T1 (CK). Under T3 treatment, leaf area and leaf dry weight increased significantly by 13.2% and 52.9%, respectively, compared to T1 (CK). Under T2 treatment, leaf area and leaf area index increased significantly by 9.7% and 23.53%, respectively, compared to T1 (CK). Moreover, compared to T1 (CK), the number of leaves on new branches under T4, T3, and T2 treatments increased significantly by 58.2%, 32.6%, and 14.0%, respectively. The basal stem diameter of the plants increased significantly by 48.8%, 33.1%, and 18.5%, respectively. Additionally, the plant canopy width increased significantly by 19.2%, 9.0%, and 12.4% under T4, T3, and T2 treatments, respectively.

**Table 1:** Effects of different leaf treatments on *Kandelia obovata* seedlings, new shoots, and leaves.

	T1 (CK)	T2	T3	T4
Height/cm	49.60 ± 1.29a	57.40 ± 3.49b	60.20 ± 2.20b	66.00 ± 2.88bc
Basal stem/mm	38.20 ± 5.84a	40.28 ± 5.04ab	50.85 ± 4.67ab	56.86 ± 4.24b
Crown width/cm	67.60 ± 2.84a	76.00 ± 2.33ab	73.70 ± 2.93ab	80.60 ± 6.07b
New branch length/cm	19.70 ± 0.95a	22.70 ± 2.50a	31.83 ± 2.38b	32.67 ± 2.20b
New branch diameter/cm	4.07 ± 0.31a	4.91 ± 0.30a	6.25 ± 0.49b	7.42 ± 0.40b
New branch leaf number	14.33 ± 3.38a	16.33 ± 3.84ab	19.00 ± 4.73ab	22.67 ± 2.91b
LA/cm^2^·leaf^-1^	6.82 ± 0.89a	7.48 ± 0.99ab	7.72 ± 0.70ab	9.71 ± 0.88b
DW/g·leaf^-1^	0.17 ± 0.01a	0.21 ± 0.01a	0.26 ± 0.02b	0.31 ± 0.02b
LAI/m^2^·m^-2^	58.58 ± 3.25a	78.92 ± 1.56b	91.08 ± 3.23c	109.08 ± 1.74d

### Effects of different treatments on soil physical and chemical properties

As shown in **Table 2**, compared with T1 (CK), soil pH under T4, T3, and T2 treatments decreased by 3.85%, 2.57%, and 1.16%, respectively, though the differences were not statistically significant. Soil total phosphorus (TP) under T4 treatment increased by 5.33% compared to T1 (CK), but this difference was also not significant. However, soil organic matter (SOM) under T4 treatment significantly increased by 16.51% compared to T1 (CK), while soil available nitrogen (AN) under T3 treatment showed a significant increase of 6.17%. Soil available phosphorus (AP) under T4, T3, and T2 treatments significantly increased by 32.05%, 26.00%, and 36.82%, respectively, compared to T1 (CK). In contrast, soil ammonium nitrogen (NH_4_-N) under T4, T3, and T2 treatments significantly decreased by 66.04%, 56.98%, and 79.16%, respectively. Similarly, soil nitrate nitrogen (NO_3_-N) under T4, T3, and T2 treatments significantly decreased by 59.71 mg·kg^-1^, 60.68 mg·kg^-1^, and 62.04 mg·kg^-1^, respectively.

**Table 2:** Effects of different foliar treatments on soil physicochemical properties.

	T1 (CK)	T2	T3	T4
pH	7.79 ± 0.23a	7.49 ± 0.27a	7.59 ± 0.05a	7.70 ± 0.09a
SOM/g·kg^-1^	10.72 ± 0.24a	10.97 ± 0.50a	11.56 ± 0.36ab	12.49 ± 0.15b
AK/g·kg^-1^	1.91 ± 0.05b	1.94 ± 0.03b	1.86 ± 0.03ab	1.71 ± 0.08a
TP/g·kg^-1^	0.75 ± 0.01a	0.74 ± 0.04a	0.72 ± 0.03a	0.79 ± 0.03a
AP/mg·kg^-1^	17.19 ± 0.69a	22.70 ± 1.49b	21.66 ± 0.95b	23.52 ± 1.19b
TN/g·kg^-1^	1.23 ± 0.04a	1.30 ± 0.06a	1.22 ± 0.03a	1.10 ± 0.10a
AN/mg·kg^-1^	65.85 ± 2.02bc	58.13 ± 0.59a	69.91 ± 3.80c	60.08 ± 1.07ab
NH_4_^+^-N/mg·kg^-1^	335.01 ± 1.56c	198.15 ± 2.40a	213.41 ± 4.25b	201.76 ± 4.60a
NO_3_^–^N/mg·kg^-1^	69.34 ± 2.15b	7.30 ± 0.51a	8.66 ± 0.72a	9.63 ± 0.78a

### Non-targeted metabolomics analysis

**Figure 2:**
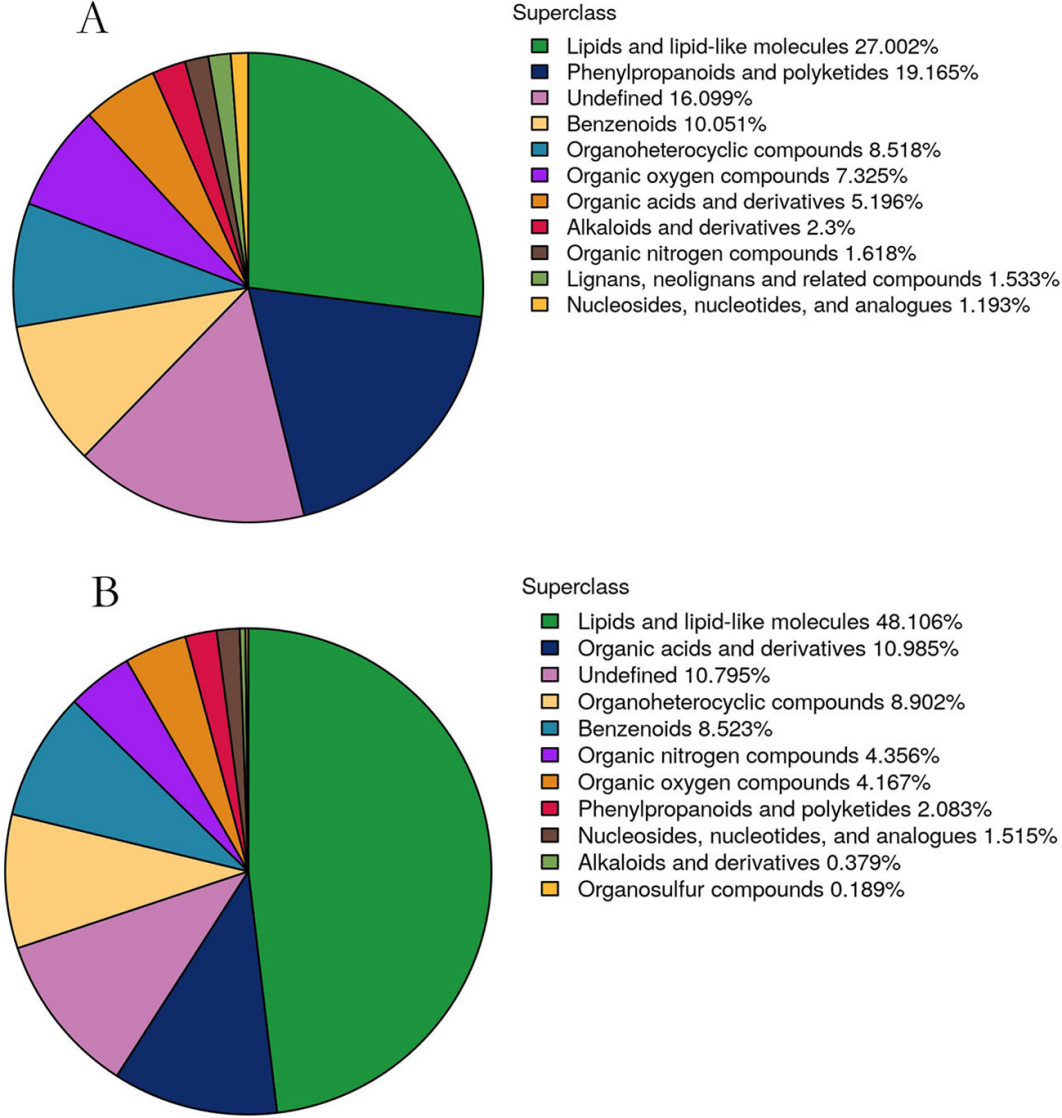
Quantitative proportions of identified metabolites in each chemical category. **(A, B)** A is the metabolite of new leaves, B is the metabolite of soil. The blocks in different colors represent various chemical classifications, and the percentages indicate the proportion of metabolites within each classification relative to the total number of metabolites. Metabolites with unspecified chemical categories are classified as undefined.

For the untargeted metabolite analysis, we selected four representative treatment samples: T1 (CK), T2, T3, and T4. The pesticides applied include abamectin, thiamethoxam, and pyriproxyfen. The benzoate salt of abamectin has been widely used as an insecticide and is approved by the U.S. Environmental Protection Agency for the control of emerald ash borer on ash trees (Poland et al., 2010). Thiamethoxam is an insecticide used to control pests such as mealybugs, leafhoppers, and whiteflies on vegetable crops (Liu and Chen, 2000). Pyriproxyfen was introduced to the U.S. in 1996 to protect cotton crops from whitefly infestations and can also be used to protect other crops. Therefore, these pesticides were applied to *Kandelia obovata* to evaluate the effects of spraying on the plant leaves and soil. A total of 24 samples (4 new leaf samples and 4 soil samples, with 3 replicates) were selected for untargeted metabolomic profiling. A total of 24 samples (4 new leaf samples and 4 soil samples, with 3 replicates each) were selected for untargeted metabolomic analysis. In the 4 leaf samples, a total of 1,174 metabolites were identified, including 481 in negative ion mode and 693 in positive ion mode, as detailed in **Supplementary Table 1**. Similarly, 528 metabolites were identified in the 4 soil samples, with 209 detected in negative ion mode and 319 in positive ion mode, as shown in **Supplementary Table 2**. Based on chemical taxonomy, primary chemical classifications were conducted for the 1,174 metabolites from the new leaves and the 528 metabolites from the soil. The quantitative proportions of metabolites in new leaves and soil are shown in **Figures 2A, B**. In the new leaves, the two most abundant chemical categories were lipids and lipid-like molecules, as well as phenylpropanoids and polyketides. In the soil samples, the two dominant chemical categories were lipids and lipid-like molecules, along with organic acids and their derivatives. Studies have shown that lipids and lipid-like molecules, as well as organic acids and their derivatives, are the primary chemical components in laboratory-fermented broccoli. Phenylpropanoids and related plant polyketides can attract pollinators, support the growth of secondary cell walls, provide protection against various plant diseases, and interact with beneficial soil microorganisms (Yu and Jez, 2008; Xu X. et al., 2021).

**Figure 3:**
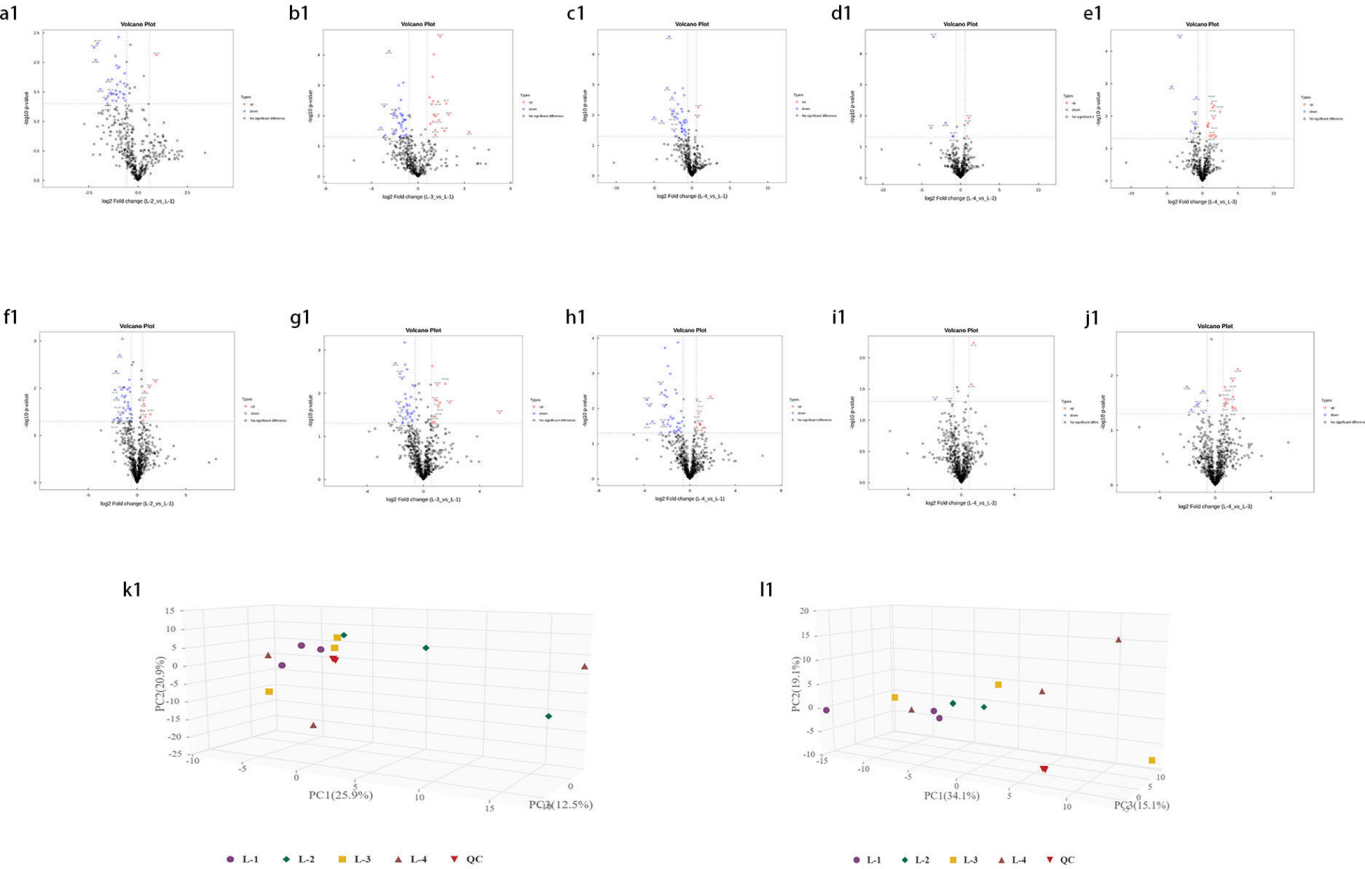
Volcano plot of identified metabolites. **(a1–e1)** represent the negative ion mode, while **(f1–j1)** represent the positive ion mode. Red points indicate upregulated differential metabolites, blue points indicate downregulated metabolites, and gray points represent metabolites with insignificant expression and small differences. **(k1)** 3D score plot of principal component analysis in the negative ion mode and **(l1)** in the positive ion mode.

Univariate statistical analysis of all identified metabolites. A fold change (FC) analysis was performed on all metabolites (including undefined chemical categories). Differential metabolites with FC > 1.5 or FC < 0.67 and P < 0.05 were highlighted. The volcano plots for young leaves and soil samples are shown in **Figures 3 (a1-j1)**, **4 (a2-j2)**, respectively. Compared with L1 (CK), in L2, there are 13 downregulated metabolites (8 in negative ion mode, 5 in positive ion mode) and 3 upregulated metabolites (2 in negative ion mode, 1 in positive ion mode). In L3, there are 16 downregulated metabolites (8 in negative ion mode, 8 in positive ion mode) and 9 upregulated metabolites (7 in negative ion mode, 2 in positive ion mode). In L4, there are 20 downregulated metabolites (9 in negative ion mode, 11 in positive ion mode) and 1 upregulated metabolite (in positive ion mode). Compared with L4, L2 has 2 downregulated metabolites (both in negative ion mode), L3 has 1 downregulated metabolite (in positive ion mode) and 4 upregulated metabolites (1 in negative ion mode, 3 in positive ion mode). In the negative ion mode, the most upregulated metabolite in the comparison between L1 and L2 is Agnuside, while in the positive ion mode, the most upregulated metabolites are 1-(3-Aminophenyl)ethenone and alpha-D-Glucopyranoside, methyl. 9-HOTrE, [2-(3,4-Dihydroxyphenyl)-5-hydroxy-7-methoxy-4-oxo-3-[(2S,3R,4R,5R,6S)-3,4,5-trihydroxy-6-methyloxan-2-yl]oxychromen-8-yl] acetate, and Morin (negative ion mode), as well as (6,6-Dimethylbicyclo[3.1.1]hept-2-yl)methyl 6-O-[(2R,3R,4R)-3,4-dihydroxy-4-(hydroxymethyl)tetrahydro-2-furanyl]-beta-D-glucopyranoside, 16-hydroxy-2,2,4a,6a,6b,11,11,14b-octamethyl-4H,4bH,5H,6H,7H,8H,9H,10H,12H,12aH,14H,14aH,15H,16H,16aH-piceno[3,4-d][1,3]dioxine-8a-carboxylic acid, 3-[4-Methyl-1-(2-methylpropanoyl)-3-oxocyclohexyl]butanoic acid, Globularin, and Syringin (positive ion mode) are the seven most downregulated metabolites in the comparison between L1 and L2. In the negative ion mode, the most upregulated metabolite in the comparison between L1 and L3 is Quinic acid, while in the positive ion mode, it is Drofenine. (1S,4aS,7S,7aS)-1-[(2S,3R,4S,5S,6R)-6-[[(E)-3-(3,4-dihydroxyphenyl)prop-2-enoyl]oxymethyl]-3,4,5-trihydroxyoxan-2-yl]oxy-7-hydroxy-7-methyl-4a,5,6,7a-tetrahydro-1H-cyclopenta[c]pyran-4-carboxylic acid, 13-Oxotrideca-9,11-dienoic acid, and 9-HOTrE (negative ion mode), as well as 18-Deoxyleucopaxillone A, 3-[4-Methyl-1-(2-methylpropanoyl)-3-oxocyclohexyl]butanoic acid and (S)-7-(((2-O-6-Deoxy-alpha-L-mannopyranosyl)-beta-D-glucopyranosyl)oxy)-2,3-dihydro-5-hydroxy-2-(3-hydroxy-4-methoxyphenyl)-4H-1-benzopyran-4-one (positive ion mode) are the six most downregulated metabolites in the comparison between L1 and L3. In the negative ion mode, there are no upregulated metabolites in the comparison between L1 and L4, while in the positive ion mode, 2-{[6-O-(beta-D-Glucopyranosyl)-beta-D-glucopyranosyl]oxy}-2-phenylacetamide is the most upregulated metabolite. (1S,4aS,7S,7aS)-1-[(2S,3R,4S,5S,6R)-6-[[(E)-3-(3,4-dihydroxyphenyl)prop-2-enoyl]oxymethyl]-3,4,5-trihydroxyoxan-2-yl]oxy-7-hydroxy-7-methyl-4a,5,6,7a-tetrahydro-1H-cyclopenta[c]pyran-4-carboxylic acid, Ginsenoside F2, 13-Oxotrideca-9,11-dienoic acid, (6,6-Dimethylbicyclo[3.1.1]hept-2-yl)methyl (negative ion mode), as well as 6-O-[(2R,3R,4R)-3,4-dihydroxy-4-(hydroxymethyl)tetrahydro-2-furanyl]-beta-D-glucopyranoside, 18-Deoxyleucopaxillone A, and 3-[4-Methyl-1-(2-methylpropanoyl)-3-oxocyclohexyl]butanoic acid (positive ion mode) are the seven most downregulated metabolites in the comparison between L1 and L4. In the negative ion mode, the most downregulated metabolites in the comparison between L4 and L2 are 4-Caffeoylquinic acid and 4-O-Caffeoylquinic acid; in the positive ion mode, no downregulated metabolites are observed. No upregulated metabolites were found in the comparison between L4 and L2. In the negative ion mode, the most downregulated metabolite in the comparison between L4 and L3 is 16-hydroxy-2,2,4a,6a,6b,11,11,14b-octamethyl-4H,4bH,5H,6H,7H,8H,9H,10H,12H,12aH,14H,14aH,15H,16H,16aH-piceno[3,4-d][1,3]dioxine-8a-carboxylic acid, while no downregulated metabolites were found in the positive ion mode. 2-{[6-O-(beta-D-Glucopyranosyl)-beta-D-glucopyranosyl]oxy}-2-phenylacetamide, (6beta,22E)-6-Hydroxystigmasta-4,22-dien-3-one, and Citropten (positive ion mode), as well as PG 34:1 (positive ion mode), are the four most upregulated metabolites in the comparison between L4 and L3.

**Figure 4:**
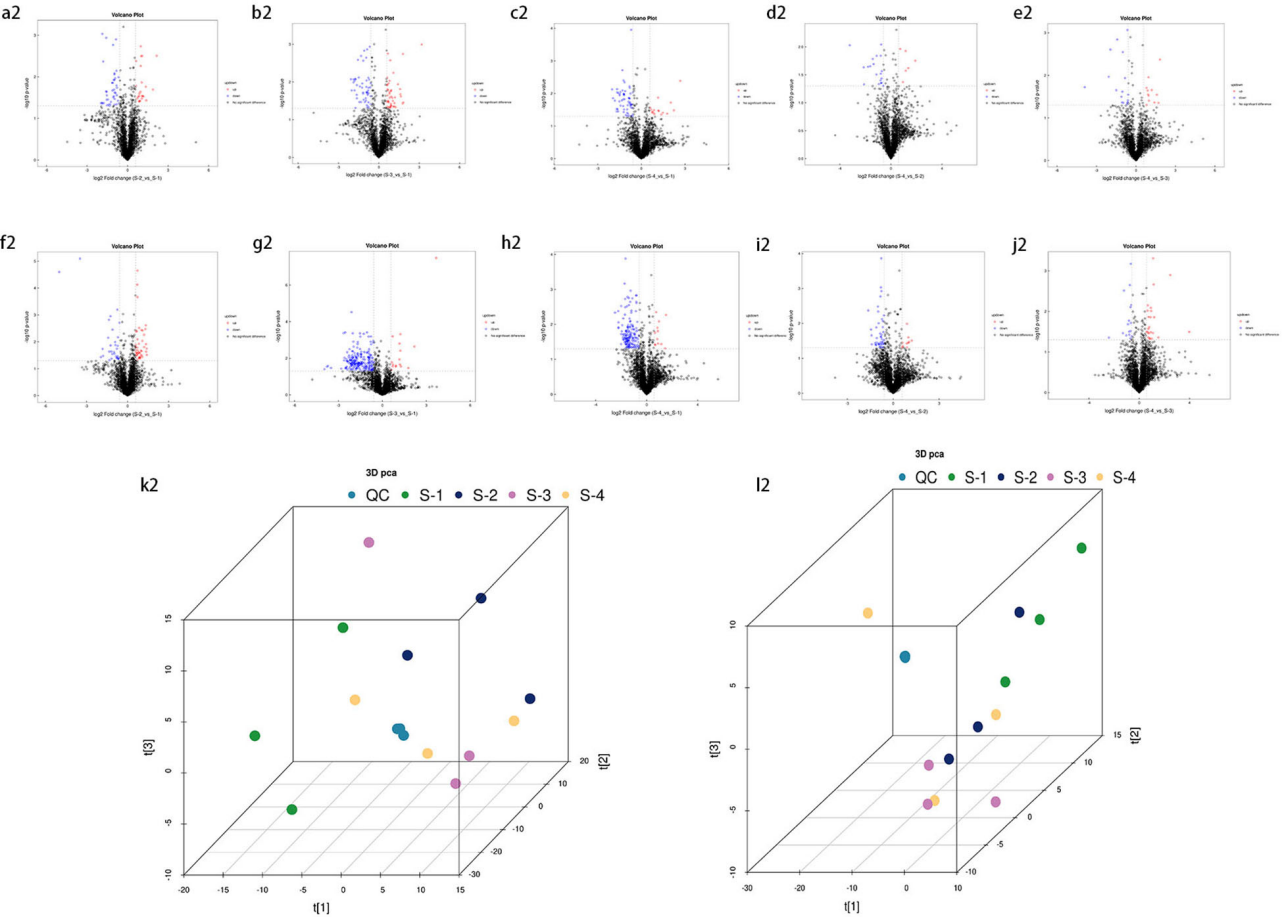
Volcano plot of identified metabolites. Panels **(a2–e2)** represent the negative ion mode, while **(f2–j2)** correspond to the positive ion mode. Red dots indicate upregulated differential metabolites, blue dots represent downregulated differential metabolites, and gray dots denote metabolites with insignificant expression differences. Additionally, panel **(k2)** presents the **3D principal component analysis (PCA) score plot** for the **negative ion mode**, while panel **(l2)** shows the corresponding plot for the **positive ion mode**.

Compared to S1 (CK), S2 exhibited 10 downregulated metabolites (7 in the negative ion mode and 3 in the positive ion mode) and 12 upregulated metabolites (10 in the negative ion mode and 2 in the positive ion mode). In S3, 20 metabolites were downregulated (8 in the negative ion mode and 12 in the positive ion mode), while 7 metabolites were upregulated (5 in the negative ion mode and 2 in the positive ion mode). S4 showed 13 downregulated metabolites (6 in the negative ion mode and 7 in the positive ion mode) and 3 upregulated metabolites (2 in the negative ion mode and 1 in the positive ion mode). Compared to S4, S2 contained 4 downregulated metabolites, all in the negative ion mode, whereas S3 exhibited 3 downregulated metabolites (all in the negative ion mode) and 1 upregulated metabolite (in the negative ion mode). In the comparison between S1 and S2, the most upregulated metabolite in the negative ion mode was Mestranol, whereas C17-sphinganine exhibited the highest upregulation in the positive ion mode. Conversely, the most downregulated metabolites in S2 included Dodecyl sulfate, (Z)-5,8,11-trihydroxyoctadec-9-enoic acid, and Zinniol in the negative ion mode, and Gabapentin, Dicyclohexylamine, and 2,6-di-tert-butyl-4-hydroxymethylphenol in the positive ion mode. In the comparison between S1 and S3, the most upregulated metabolite in the negative ion mode was Oligomycin B, while in the positive ion mode, N-myristoylsphinganine exhibited the highest upregulation. In contrast, the most downregulated metabolites in S3 included Deoxycholic acid, Dodecyl sulfate, and 1,2-dioleoyl-sn-glycero-3-phosphoethanolamine in the negative ion mode, and Glycodeoxycholic acid, Ursodeoxycholic acid, and 1-(1Z-octadecenyl)-2-(9Z-octadecenoyl)-sn-glycero-3-phosphocholine in the positive ion mode. In the comparison between S1 and S4, the most upregulated metabolite in the negative ion mode was 15-cyclohexylpentanorprostaglandin F2α, while in the positive ion mode, Caffeine showed the highest upregulation. Conversely, the most downregulated metabolites in S4 included Deoxycholic acid, γ-linolenic acid, and Heptadecanoic acid in the negative ion mode, and Glycodeoxycholic acid, 1-(1Z-octadecenyl)-2-(9Z-octadecenoyl)-sn-glycero-3-phosphocholine, and Ursodeoxycholic acid in the positive ion mode. In the comparison between S4 and S2, the most downregulated metabolite in the negative ion mode was Octacosanoic acid, while in the positive ion mode, Met-Met-Arg showed the highest downregulation. Notably, no upregulated metabolites were observed in S2 relative to S4. Finally, in the comparison between S4 and S3, the most downregulated metabolite in the negative ion mode was 2-(2-hydroxybut-3-en-2-yl)-3a,6,6,9a-tetramethyl-2,4,5,5a,7,8,9,9b-octahydro-1H-benzo[e][1]benzofuran-4,5-diol, while no downregulated metabolites were detected in the positive ion mode. In contrast, the most upregulated metabolite in S3 was Ricinoleic acid in the negative ion mode, with no upregulated metabolites identified in the positive ion mode.

### Multivariate statistical analysis

**Figure 5:**
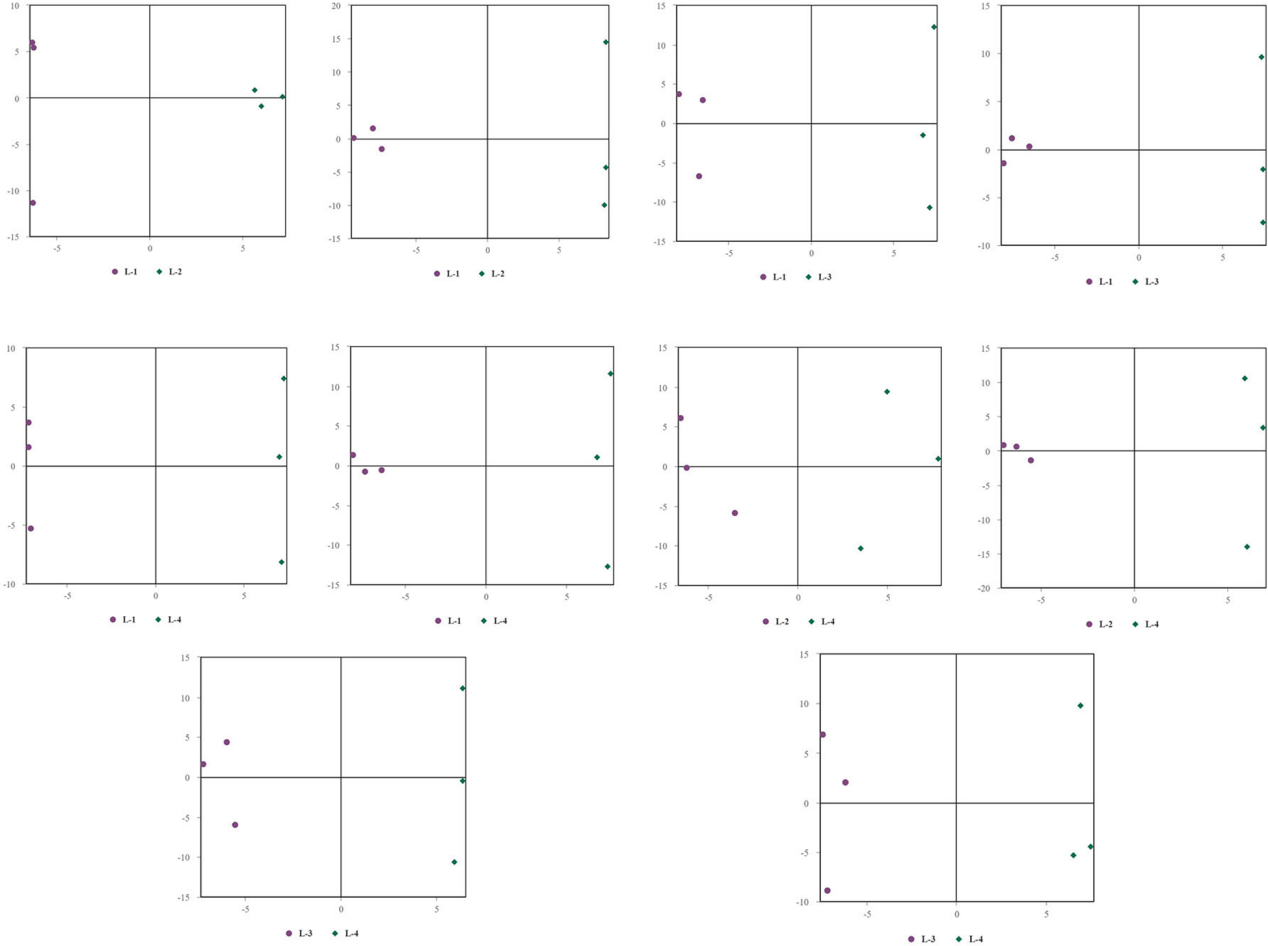
PCA analysis in leaf samples.

**Figure 6:**
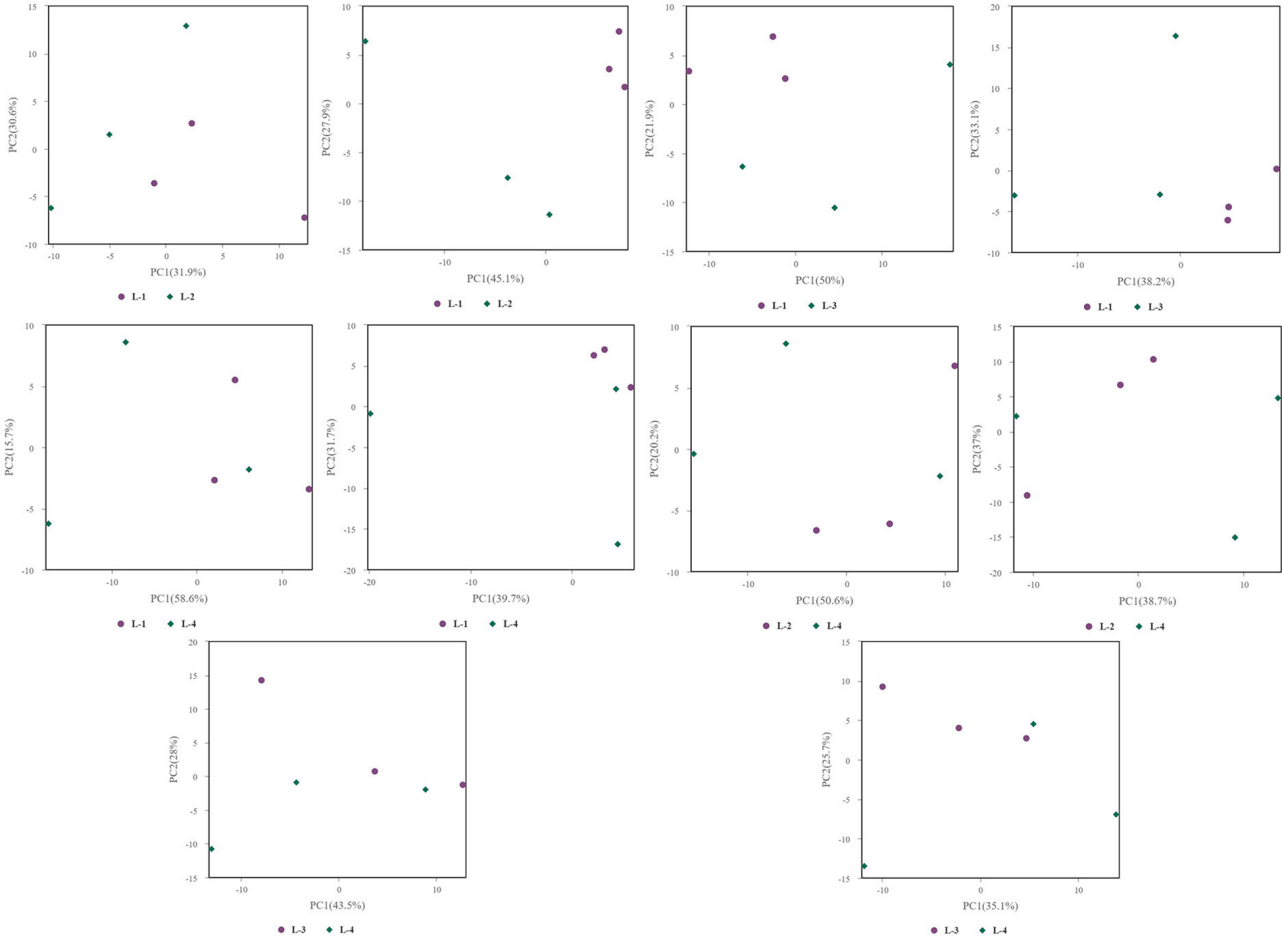
PCA analysis in soil samples.

**Figure 7:**
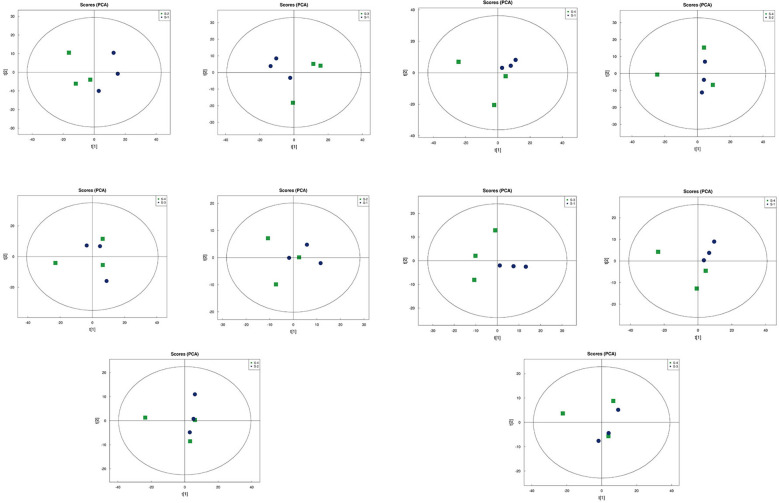
OPLS-DA analysis in leaf samples.

**Figure 8:**
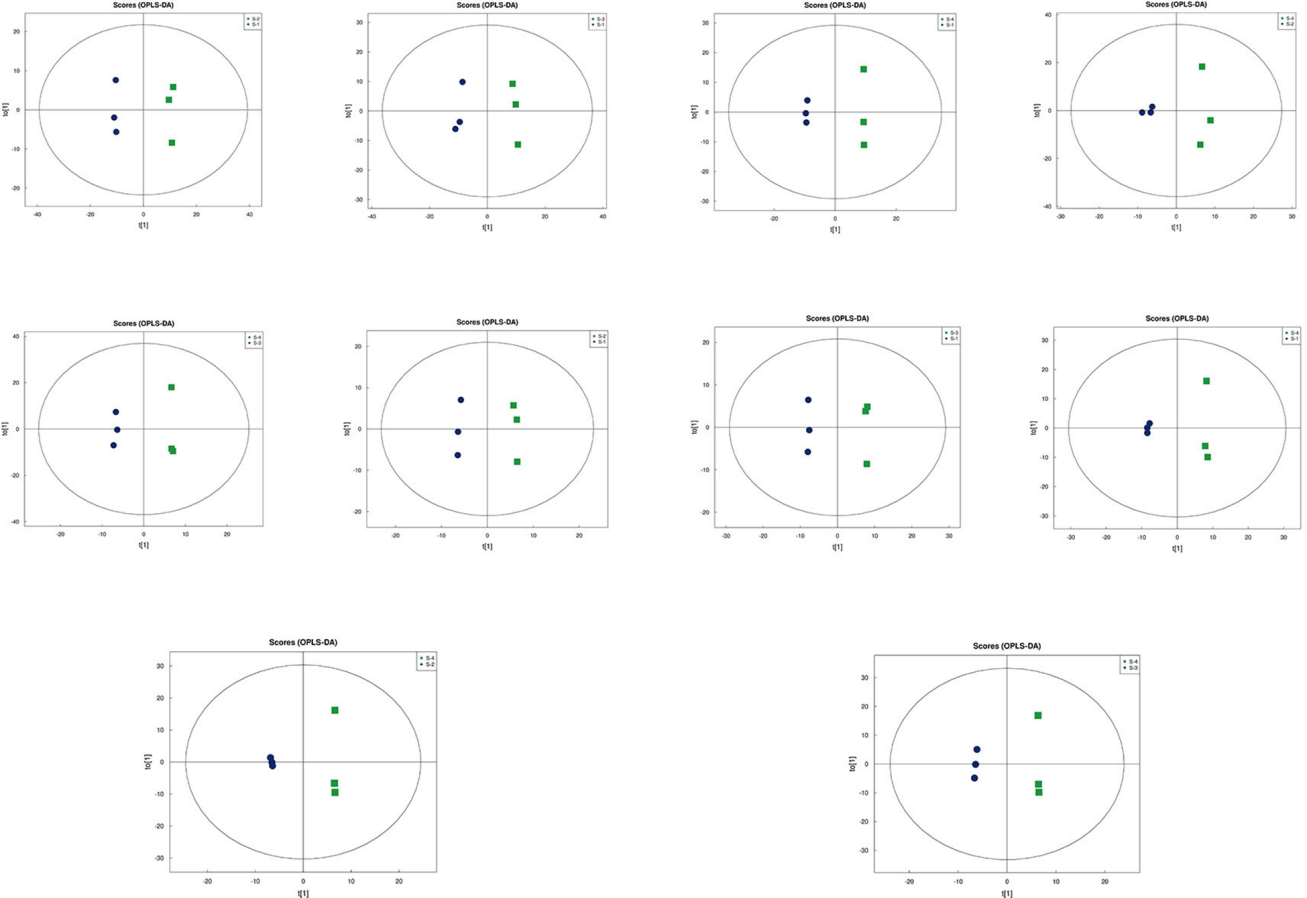
OPLS-DA analysis in soil samples.

**Figure 9:**
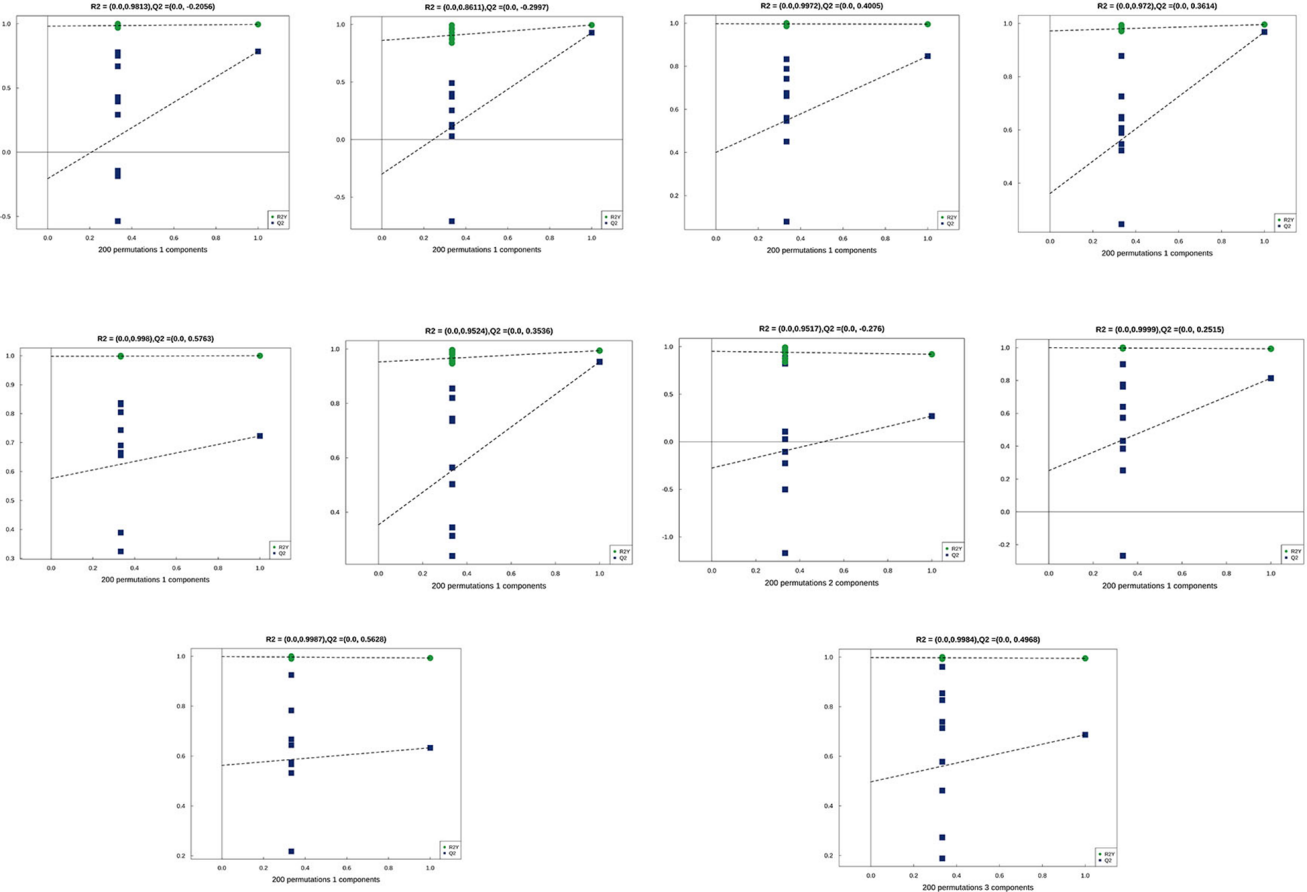
Permutation testing in leaf samples.

**Figure 10:**
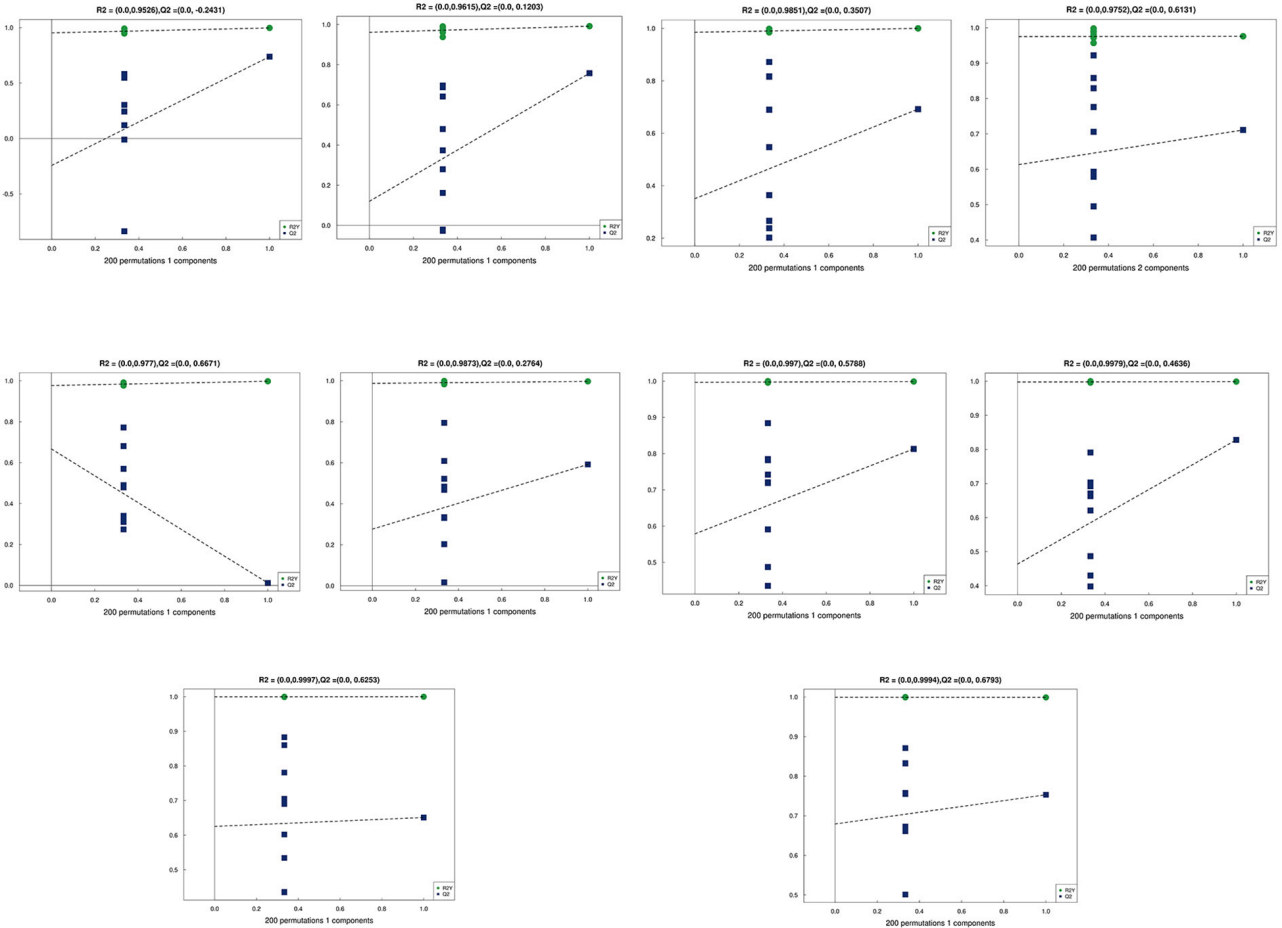
Permutation testing in soil samples. The horizontal axis in the figure represents the degree of permutation retention, which is the proportion consistent with the order of the Y variables in the original model, while the vertical axis represents the values of R_2_ and Q_2_. The green dots represent R_2_, the blue dots represent Q_2_, and the two dashed lines represent the regression lines for R_2_ and Q_2_, respectively. The R_2_ and Q_2_ in the upper right corner indicate that the permutation retention is equal to 1, which is the R_2_ and Q_2_ values of the original model.

**Figure 11:**
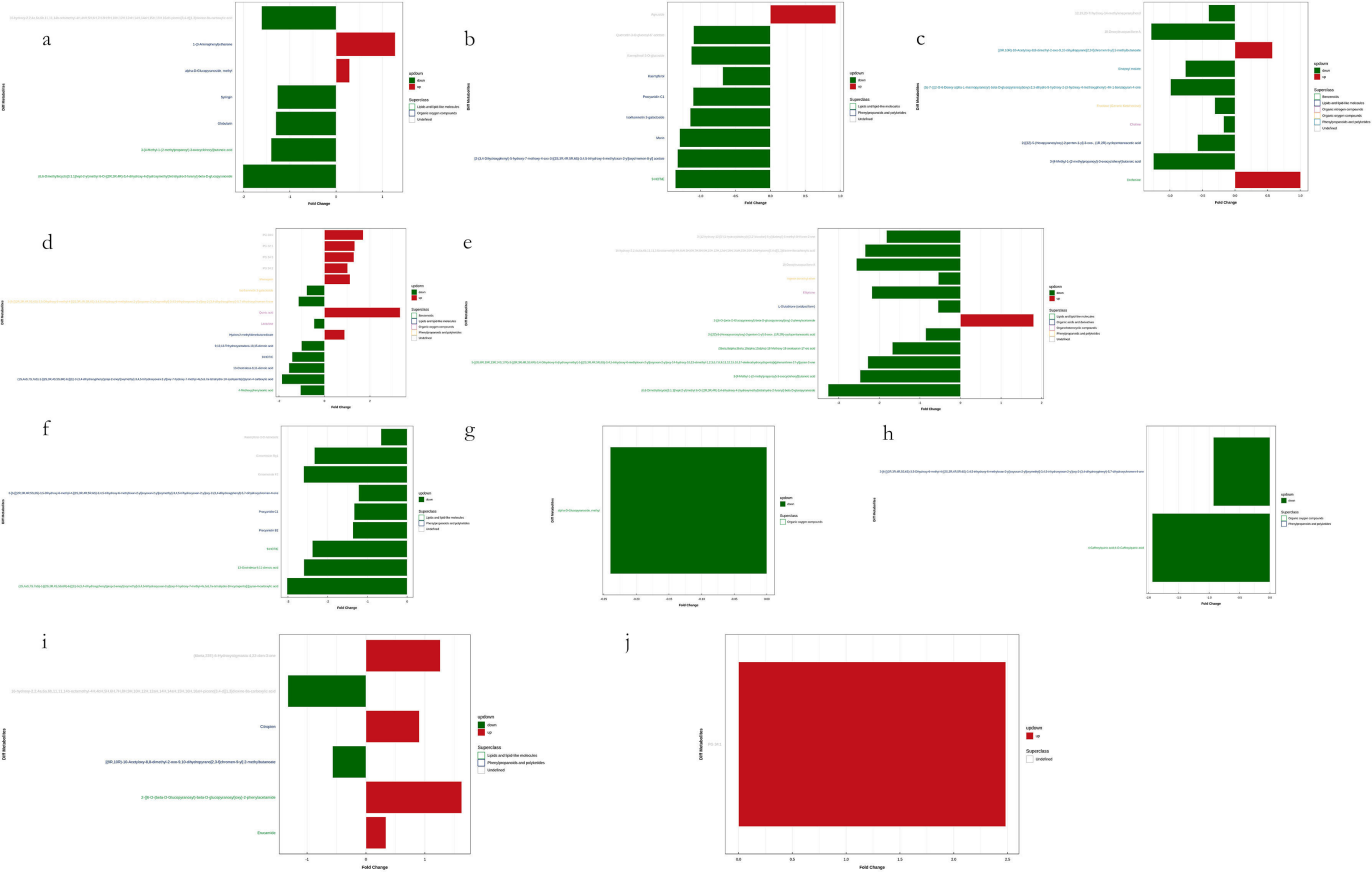
Visualization of significant differential metabolites in Leaf samples. Six comparisons among four groups of samples in each mode. **(a, c, e, g, i)** are negative ion mode and **(b, d, f, h, j)** are positive ion mode. Blocks in red are upregulated metabolites and blocks in green are downregulated.

**Figure 12:**
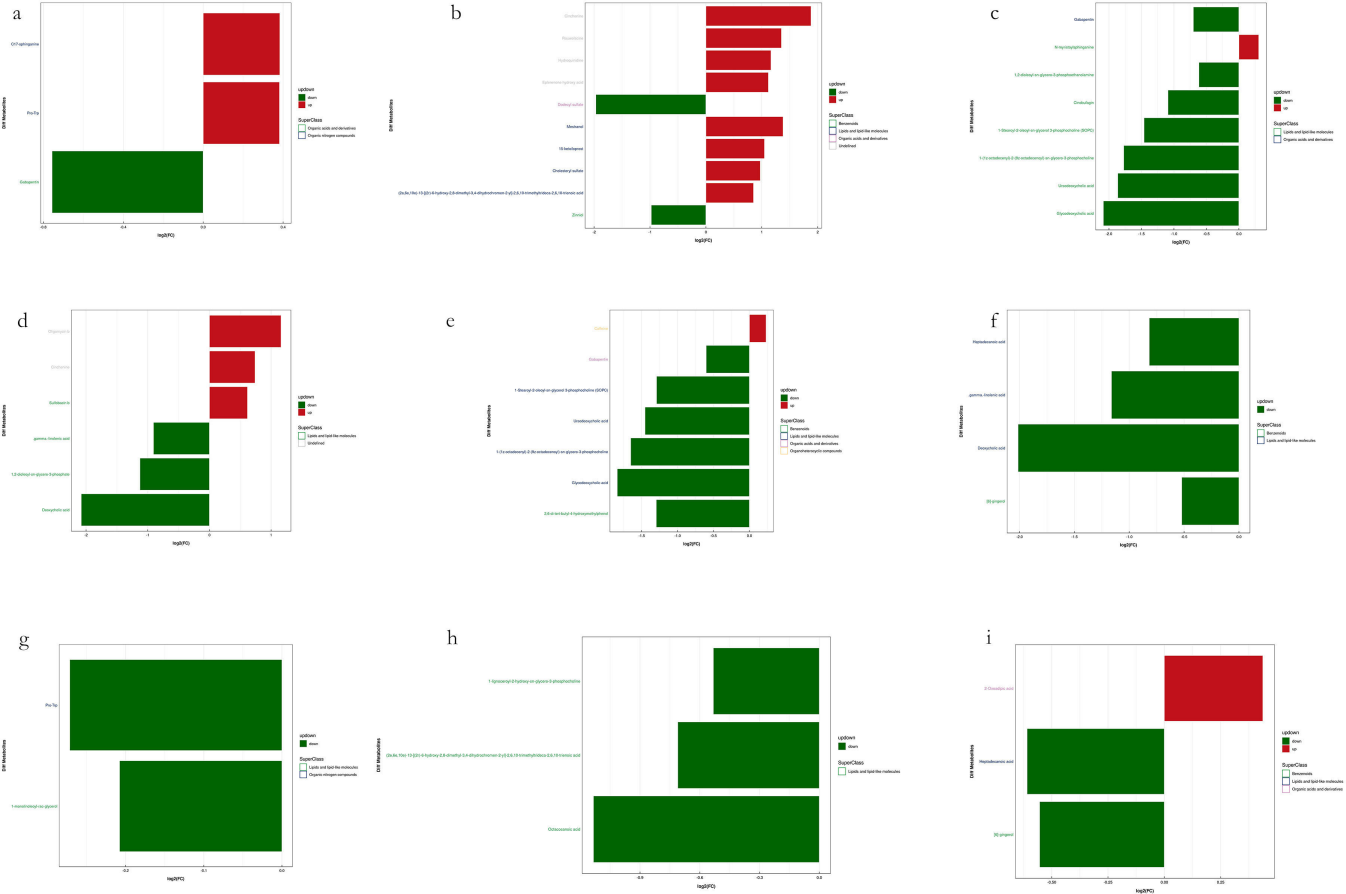
Visualization of significant differential metabolites in Soil samples. Six comparisons among four groups of samples in each mode. **(a, c, e, g, i)** are negative ion mode and **(b, d, f, h)** are positive ion mode. Blocks in red are upregulated metabolites and blocks in green are downregulated.

Principal Component Analysis (PCA) 3D Score Plot. As shown in **Figures 3k1, l1** and **Figures 4k2, l2**, a principal component analysis (PCA) was performed to evaluate metabolic variations among samples. The first, second, and third principal components (PC1, PC2, and PC3) explained 25.9%, 20.9%, and 12.5% of the variance in the negative ion mode, and 34.1%, 19.1%, and 15.1% of the variance in the positive ion mode, respectively. **Figure 3** demonstrates that samples L1 and L2 cluster together, whereas L3 and L4 form a separate cluster, indicating a clear separation between the two groups. When combined with the volcano plot results from univariate statistical analysis, the PCA analysis confirms that L1 and L2 are distinct from L3 and L4, reflecting significant metabolic differences between these groups. Similarly, **Figure 4** shows that samples S1 and S2 cluster separately from S3 and S4, suggesting a clear metabolic distinction between these groups. The PCA results, in conjunction with the volcano plot analysis, indicate that S1 and S2 are independent from S3 and S4, highlighting significant differences in metabolic profiles. Overall, these findings suggest that metabolite composition is influenced by defoliation and pesticide spraying treatments. Multivariate statistical analysis includes Principal Component Analysis (PCA) and Orthogonal Partial Least Squares Discriminant Analysis (OPLS-DA). PCA is an unsupervised data analysis method that performs linear reorganization of all previously identified metabolites to form a set of new composite variables. At the same time, several composite variables are selected based on the problem being analyzed to reflect as much information as possible from the original variables, thus achieving dimensionality reduction. Additionally, PCA of metabolites can generally reflect the variability between and within sample groups. Therefore, in data analysis, PCA is typically applied first to observe the overall distribution trends of samples between groups and the differences between samples across groups. To distinguish the accumulated metabolic differences between the leaf treatment group and the control group, PCA is first used to perform the analysis, obtaining the PCA score plots for five principal components, i.e., cationic PCA score plot and anionic PCA score plot (leaf group in **Figure 5**, soil group in **Figure 6**). The PCA model parameters obtained from 7-fold cross-validation are as follows: in L1 vs L2, the cationic PCA model has R_2_X (cum) = 0.821, and the anionic PCA model has R_2_X (cum) = 0.851; in L1 vs L3, the cationic PCA model has R_2_X (cum) = 0.855, and the anionic PCA model has R_2_X (cum) = 0.85; in L1 vs L4, the cationic PCA model has R_2_X (cum) = 0.856, and the anionic PCA model has R_2_X (cum) = 0.893; in L2 vs L4, the cationic PCA model has R_2_X (cum) = 0.863, and the anionic PCA model has R_2_X (cum) = 0.889; in L3 vs L4, the cationic PCA model has R_2_X (cum) = 0.835, and the anionic PCA model has R_2_X (cum) = 0.894. In S1 vs S2, the cationic PCA model has R_2_X (cum) = 0.795, and the anionic PCA model has R_2_X (cum) = 0.799; in S1 vs S3, the cationic PCA model has R_2_X (cum) = 0.754, and the anionic PCA model has R_2_X (cum) = 0.84; in S1 vs S4, the cationic PCA model has R_2_X (cum) = 0.845, and the anionic PCA model has R_2_X (cum) = 0.866; in S2 vs S4, the cationic PCA model has R_2_X (cum) = 0.808, and the anionic PCA model has R_2_X (cum) = 0.77; in S3 vs S4, the cationic PCA model has R_2_X (cum) = 0.835, and the anionic PCA model has R_2_X (cum) = 0.895. The closer R_2_X is to 1, the more stable and reliable the model is, indicating that the PCA model established is stable and can be used for subsequent metabolic difference analysis. To more accurately display the metabolic differences between the leaf treatment group and the control group, a supervised Orthogonal Partial Least Squares Discriminant Analysis (OPLS-DA) model was used for further analysis. The leaf treatment group samples are primarily distributed on the negative half-axis of PC1, while the control group samples are primarily distributed on the positive half-axis of PC1, indicating that the model can effectively differentiate between the two treatment types for both leaf and soil samples (**Figures 7**, **8**). A permutation test was performed on the OPLS-DA model (leaf treatment in **Figure 9**, soil treatment in **Figure 10**), where the initial OPLS-DA model on the right shows a Q_2_ value much higher than the Q_2_ value of the random model on the left, demonstrating that the OPLS-DA model is not overfitted and has good reliability. For each pattern, four groups underwent six rounds of OPLS-DA, and all OPLS-DA models passed the permutation test, with Q_2_ > 5, indicating that the model is stable and reliable. Differential metabolites with VIP > 1 and p-value < 0.05 were selected as significantly different metabolites, as shown in **Figures 11**, **12**.

Among the upregulated metabolites identified in leaf samples, the most frequently detected and recurrent compounds included quinic acid, 2-{[6-O-(β-D-glucopyranosyl)-β-D-glucopyranosyl]oxy}-2-phenylacetamide, phosphatidylglycerols (PGs) such as PG 30:0, PG 32:1, PG 34:3, and PG 34:2, as well as maesopsin, 1-(3-aminophenyl)ethenone, drofenine, agnuside, and Hydron; 2-methylidenebutanedioate. Additionally, other highly recurrent metabolites included [(9R,10R)-10-acetyloxy-8,8-dimethyl-2-oxo-9,10-dihydropyrano[2,3-f]chromen-9-yl] 2-methylbutanoate and α-D-glucopyranoside, methyl. Conversely, the most frequently detected downregulated metabolites in leaf samples included (6,6-dimethylbicyclo[3.1.1]hept-2-yl)methyl 6-O-[(2R,3R,4R)-3,4-dihydroxy-4-(hydroxymethyl)tetrahydro-2-furanyl]-β-D-glucopyranoside, (1S,4aS,7S,7aS)-1-[(2S,3R,4S,5S,6R)-6-[[(E)-3-(3,4-dihydroxyphenyl)prop-2-enoyl]oxymethyl]-3,4,5-trihydroxyoxan-2-yl]oxy-7-hydroxy-7-methyl-4a,5,6,7a-tetrahydro-1H-cyclopenta[c]pyran-4-carboxylic acid, 13-oxotrideca-9,11-dienoic acid, and 18-deoxyleucopaxillone A. Notably, the Ginsenosides (Ginsenoside F2 and Ginsenoside Rg1), along with 9-hydroperoxyoctadecatrienoic acid (9-HOTrE), 3-[4-methyl-1-(2-methylpropanoyl)-3-oxocyclohexyl]butanoic acid, and ellipticine, were also significantly downregulated. Furthermore, complex secondary metabolites such as 16-hydroxy-2,2,4a,6a,6b,11,11,14b-octamethyl-4H,4bH,5H,6H,7H,8H,9H,10H,12H,12aH,14H,14aH,15H,16H,16aH-piceno[3,4-d][1,3]dioxine-8a-carboxylic acid and 5-[(3S,8R,10R,13R,14S,17R)-3-[(2R,3R,4R,5S,6R)-3,4-dihydroxy-6-(hydroxymethyl)-5-[(2S,3R,4R,5R,6S)-3,4,5-trihydroxy-6-methyloxan-2-yl]oxyoxan-2-yl]oxy-14-hydroxy-10,13-dimethyl-1,2,3,6,7,8,9,11,12,15,16,17-dodecahydrocyclopenta[a]phenanthren-17-yl]pyran-2-one exhibited consistent downregulation.

In the soil metabolomic analysis, the most frequently upregulated metabolites included Cinchonine, Mestranol, Rauwolscine, Hydroquinidine, Eplerenone hydroxy acid, 15-ketoiloprost, Oligomycin B, and Cholesteryl sulfate. Additionally, (2E,6E,10E)-13-[(2R)-6-hydroxy-2,8-dimethyl-3,4-dihydrochromen-2-yl]-2,6,10-trimethyltrideca-2,6,10-trienoic acid was highly recurrent. Furthermore, Sulfobacin B, C17-sphinganine, Pro-Trp (proline-tryptophan dipeptide), N-myristoylsphinganine, and caffeine were among the most prominent upregulated metabolites in soil samples. Lastly, the most frequently downregulated metabolites identified in leaf samples included glycodeoxycholic acid, deoxycholic acid, ursodeoxycholic acid, 1-(1Z-octadecenyl)-2-(9Z-octadecenoyl)-sn-glycero-3-phosphocholine, and dodecyl sulfate. The observed downregulation of these metabolites suggests alterations in lipid metabolism, bile acid pathways, and phospholipid signaling. The differential regulation of metabolites in both leaf and soil samples suggests significant metabolic shifts in response to environmental or experimental conditions. The upregulation of phosphatidylglycerols, ginsenosides, and secondary metabolites in leaves indicates increased biosynthetic activity related to stress adaptation, signaling, and metabolic modulation. Meanwhile, the soil metabolomic profile reflects potential microbial interactions, nutrient cycling dynamics, and modifications in soil organic matter composition.

### KEGG pathway analysis

**Figure 13:**
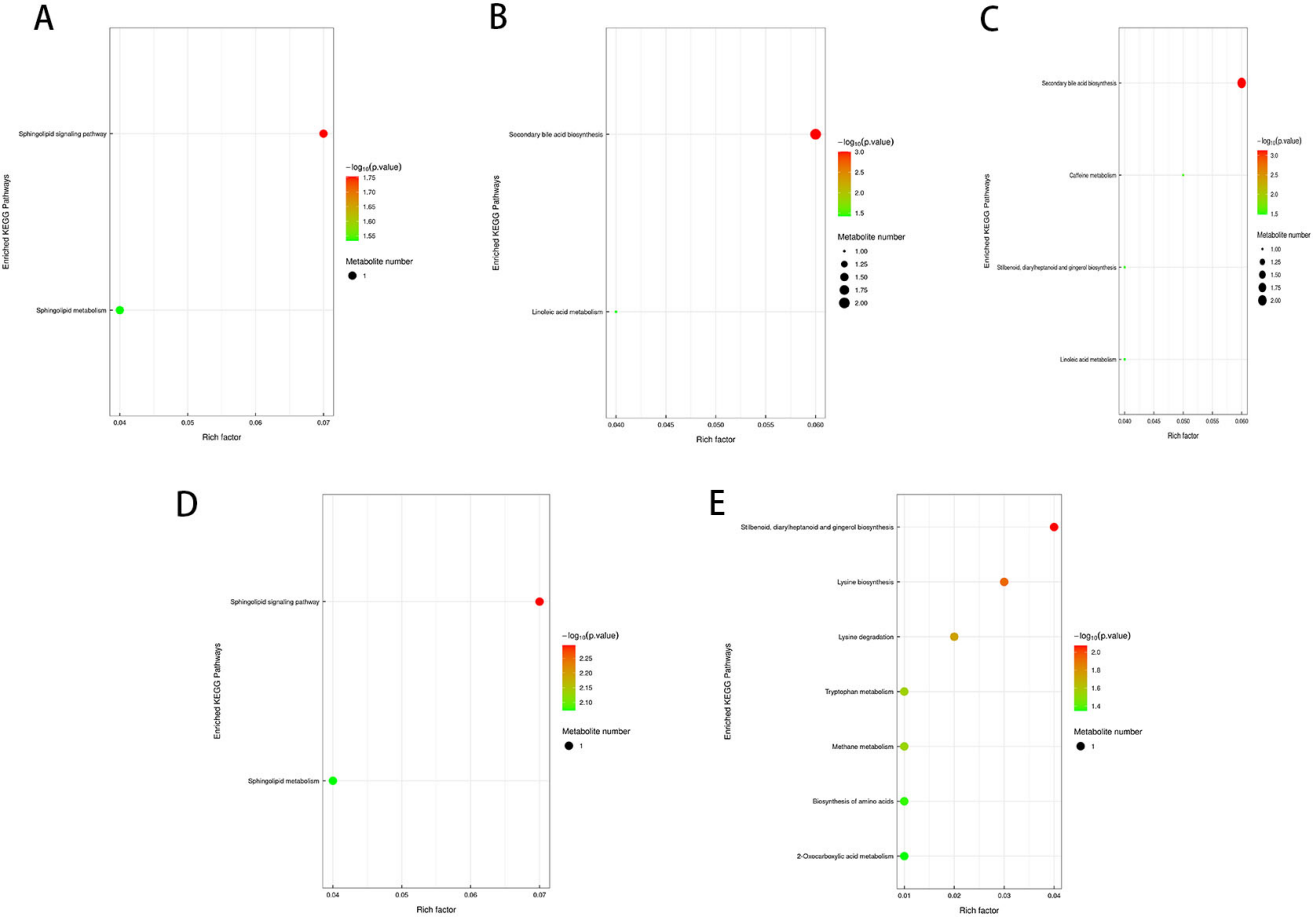
KEGG pathway enrichment bubble plot **(A–E)**. Each bubble in the plot represents a metabolic pathway based on the top 20 pathways selected according to P-value significance. The horizontal axis indicates the enrichment factor (Rich Factor ≤ 1), which represents the proportion of differentially expressed metabolites in a given KEGG pathway relative to the total annotated metabolites in that pathway. The bubble size corresponds to the number of differentially expressed metabolites in each KEGG pathway. The bubble color reflects the enrichment significance of each pathway, with a color gradient representing the P-value calculated using the Fisher’s Exact Test. The P-value is represented as -log_10_ P, where darker red colors indicate smaller P-values, signifying higher significance levels for pathway enrichment.

Prior to conducting KEGG (Kyoto Encyclopedia of Genes and Genomes) pathway annotation, differentially expressed metabolites identified under both positive and negative ion modes were merged for further analysis. Using sample comparison groups as a reference, the analytical results are as follows: KEGG pathway enrichment analysis is based on KEGG pathways, with metabolic pathways from closely related species or the target organism serving as the reference background. Fisher’s Exact Test is employed to assess the statistical significance of metabolite enrichment in each pathway. This approach allows for the identification of metabolic and signal transduction pathways that are significantly affected under the given conditions. The smaller the P-value, the more pronounced the differences in a particular metabolic pathway, indicating its potential biological significance in response to the experimental conditions. The KEGG pathway enrichment analysis of *Kandelia obovata* leaf treatment revealed distinct metabolic alterations across different comparisons. In the L2 vs. L1 comparison, among the two differentially expressed metabolites, one was enriched in four metabolic pathways, including Flavonoid biosynthesis (ko00941), Flavone and flavonol biosynthesis (ko00944), Metabolic pathways (ko01100), and Biosynthesis of secondary metabolites (ko01110). Notably, Kaempferol was significantly enriched in both the Flavone and flavonol biosynthesis and Flavonoid biosynthesis pathways (*P* < 0.05, **Figure 13A**), exhibiting a 0.62-fold downregulation compared to L1. In the L3 vs. L1 comparison, among the three differentially expressed metabolites, two were enriched in seven metabolic pathways, namely Metabolic pathways (ko01100), Phenylalanine, tyrosine and tryptophan biosynthesis (ko00400), Glycine, serine and threonine metabolism (ko00260), Glycerophospholipid metabolism (ko00564), ABC transporters (ko02010), Cholinergic synapse (ko04725), and Bile secretion (ko04976). Specifically, Quinic acid was significantly enriched in the Phenylalanine, tyrosine, and tryptophan biosynthesis pathway, while Choline was significantly enriched in the Cholinergic synapse, Glycine, serine and threonine metabolism, Glycerophospholipid metabolism, and Bile secretion pathways (*P* < 0.05, **Figure 13B**). Compared to L1, Quinic acid exhibited a 10.04-fold upregulation, whereas Choline showed a 0.89-fold downregulation. Others, L4 and L1, L2 and L4, and L3 and L4 were not enriched in the KEGG pathway. The KEGG pathway enrichment analysis of soil samples revealed significant metabolic differences across various comparisons. In the comparison between S2 and S1, two out of seven differential metabolites were enriched in four metabolic pathways, including sphingolipid metabolism (ko00600), steroid hormone biosynthesis (ko00140), metabolic pathways (ko01100), and sphingolipid signaling pathway (ko04071). Specifically, Pro-Trp was significantly enriched in both sphingolipid metabolism and sphingolipid signaling pathways (*P* < 0.05), with an upregulation of 1.3-fold compared to S1. In the comparison between S3 and S1, six out of eight differential metabolites were enriched in five pathways, including secondary bile acid biosynthesis (ko00121), bile secretion (ko04976), linoleic acid metabolism (ko00591), biosynthesis of unsaturated fatty acids (ko01040), and metabolic pathways (ko01100). Gamma-linolenic acid was significantly enriched in linoleic acid metabolism, while deoxycholic acid and ursodeoxycholic acid were significantly enriched in secondary bile acid biosynthesis (*P* < 0.05), with downregulations of 0.53-fold, 0.24-fold, and 0.27-fold, respectively, compared to S1. In the comparison between S4 and S1, six out of seven differential metabolites were enriched in nine pathways, including metabolic pathways (ko01100), secondary bile acid biosynthesis (ko00121), biosynthesis of secondary metabolites (ko01110), linoleic acid metabolism (ko00591), biosynthesis of unsaturated fatty acids (ko01040), bile secretion (ko04976), stilbenoid, diarylheptanoid, and gingerol biosynthesis (ko00945), caffeine metabolism (ko00232), and microbial metabolism in diverse environments (ko01120). Deoxycholic acid and ursodeoxycholic acid were significantly enriched in secondary bile acid biosynthesis, caffeine was enriched in caffeine metabolism, [6]-gingerol was enriched in stilbenoid, diarylheptanoid, and gingerol biosynthesis, and gamma-linolenic acid was enriched in linoleic acid metabolism (*P* < 0.05). Compared to S1, these metabolites exhibited downregulations of 0.25-fold and 0.36-fold, upregulation of 1.17-fold, and downregulations of 0.69-fold and 0.45-fold, respectively. In the comparison between S2 and S4, one out of two differential metabolites was enriched in three pathways, including sphingolipid signaling pathway (ko04071), sphingolipid metabolism (ko00600), and metabolic pathways (ko01100), with Pro-Trp significantly enriched in both sphingolipid-related pathways (*P* < 0.05), showing a downregulation of 0.83-fold compared to S2. Finally, in the comparison between S3 and S4, the two differential metabolites were enriched in ten pathways, including biosynthesis of secondary metabolites (ko01110), lysine biosynthesis (ko00300), lysine degradation (ko00310), tryptophan metabolism (ko00380), methane metabolism (ko00680), metabolic pathways (ko01100), microbial metabolism in diverse environments (ko01120), 2-oxocarboxylic acid metabolism (ko01210), biosynthesis of amino acids (ko01230), and stilbenoid, diarylheptanoid, and gingerol biosynthesis (ko00945). [6]-Gingerol was significantly enriched in the stilbenoid, diarylheptanoid, and gingerol biosynthesis pathway, while 2-oxoadipic acid was significantly enriched in multiple pathways related to lysine metabolism, tryptophan metabolism, methane metabolism, amino acid biosynthesis, and 2-oxocarboxylic acid metabolism (*P* < 0.05), with downregulation of 0.68-fold and upregulation of 1.35-fold compared to S3.

## Discussion

**Figure 14:**
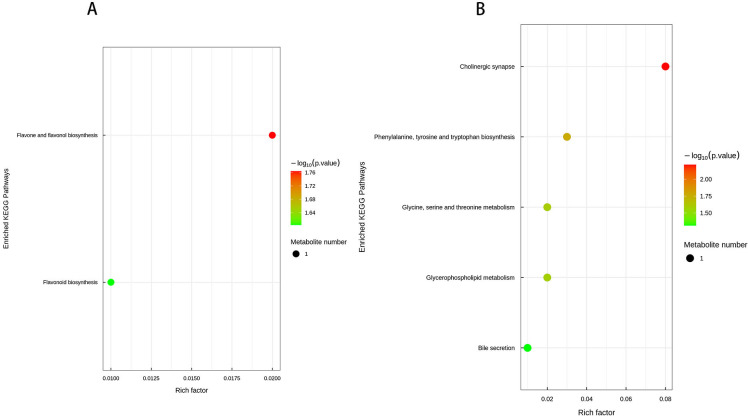
KEGG pathway enrichment bubble plot **(A, B)**. Each bubble in the plot represents a metabolic pathway, with the top 20 pathways selected based on statistical significance (P-value). The horizontal axis and bubble size indicate the impact factor in topological analysis, where larger bubbles correspond to pathways with greater impact. The vertical axis and bubble color represent the P-value, transformed using -log_10_ P, where darker colors indicate smaller P-values, signifying higher enrichment significance. The enrichment factor denotes the proportion of differentially expressed metabolites relative to the total annotated metabolites in a given pathway.

In the present study, we observed that defoliation, foliar spraying, and their combination significantly enhanced the growth of new branches in *Kandelia obovata*, as evidenced by increased length, diameter, leaf number, leaf area (LA), dry weight (DW), and leaf area index (LAI) (**Table 1**). Compared with the control group, the increase in soil organic matter (SOM) and available phosphorus (AP) content, as well as the decrease in available potassium (AK), available nitrogen (AN), ammonium nitrogen (NH_4_N), and nitrate nitrogen (NO_3_N) content, were positively correlated with defoliation and foliar spraying, with the combination treatment yielding the best results (**Table 2**). Under the combined treatment of defoliation and foliar spraying, the trend of plant growth was positively correlated with that of foliar spraying alone, but not significantly correlated with defoliation alone, indicating that foliar spraying had a slightly greater impact on the growth of *K. obovata* than defoliation alone (**Table 1**). Similar trends were observed in plant height and canopy width accumulation (**Table 1**). Additionally, these conclusions were corroborated by the identification of differential metabolites and validation through methods such as PCA, OPLS-DA, and permutation testing (**Figures 5**–**10**). The results of differential metabolites indicated that the combination of defoliation and foliar spraying produced more significant metabolic changes than either treatment alone (**Figures 11**, **12**). The KEGG pathway analysis also revealed that the combined treatment was the most effective (**Figures 13A–E**, **14A, B**). These findings suggest that the combination of defoliation and foliar spraying has a greater impact on plant growth and development than either treatment individually.

### The combined effects of defoliation and spraying treatments on the growth and development of *Kandelia obovata* and the enhancement of newly emerged leaf quality

Although the underlying triggers for the low statistical significance observed in metabolomic data under the combined treatment of defoliation and spraying remain to be elucidated, the results indicate that this combined treatment effectively promotes the growth of newly emerged branches and leaves in *Kandelia obovata*, exhibiting superior effects compared to defoliation or spraying alone. We hypothesize several possible reasons for the low significance in metabolomic responses under the combined treatment. First, the frequency and dosage of the spraying treatment may have been insufficient, potentially preventing the expression of many responsive metabolites. Second, the plant’s intrinsic adaptive growth characteristics may also contribute to this phenomenon. Specifically, during the initial phase of spraying, plants typically express stress-responsive metabolites; however, as the treatment persists, an adaptive phase may follow, in which some initially responsive metabolites return to baseline levels. We further speculate that the combined effects of defoliation and spraying might enhance the plant’s adaptive responses, leading to the observed metabolic patterns. This hypothesis is supported by the fact that despite the phenotypic changes in leaf morphology, differential metabolites and their associated pathways did not exhibit significant alterations.

### Untargeted metabolomics reveals key metabolites and metabolic pathways in *Kandelia obovata* under defoliation and pesticide treatment

The adaptive mechanisms of mangrove plants in response to defoliation and pesticide treatment are highly complex. Untargeted metabolomic analysis can extract valuable data to predict differential metabolites in both soil and leaves associated with defoliation and pesticide application, thereby providing critical insights into intricate metabolic networks. This study aims to identify novel potential biomarkers to enhance the understanding of differential metabolites and pathway mechanisms in newly developed leaves and soil of *Kandelia obovata* under different treatment conditions.

Research has shown that flavonoids, a group of secondary metabolites widely present in plants, play an important role in plant physiological metabolism, stress resistance, disease resistance, and growth development (Fürtauer et al., 2019). Flavonoids not only regulate plant growth but also influence insect behavior and metabolism, inducing avoidance or deterrence responses, and even interfering with their normal physiological functions. In severe cases, flavonoids can cause insect poisoning or even death, making them secondary metabolites with significant insecticidal activity (Zhang et al., 2004). Quercetin, a typical flavonoid, is widely distributed in various fruits, vegetables, and herbaceous plants and can enhance plants’ resistance to both biotic and abiotic stresses (Jiao et al., 2023). In this study, L2 treatment led to the relative downregulation of quercetin, suggesting that leaf plucking positively affects the healthy growth of newly formed leaves in Solanum torvum. Moreover, phenylalanine, after being catalyzed by phenylalanine ammonia-lyase, can synthesize various compounds related to plant defense (Zhou et al., 2018). In rice, phenylalanine under the action of specific proteases endows the plant with broad-spectrum resistance to pests and diseases (He et al., 2019; Zhai et al., 2019). At the same time, tyrosine (Kircher et al., 1970) and some alkaloids (Tempone et al., 2005) also exhibit properties that enhance plant resistance or deter herbivorous insects, which is consistent with the results of the L4 treatment in this study. Metabolomic analysis further revealed significant differences in the secondary metabolites between the L4 and L1 treatments, mainly characterized by a decrease in nutrients and an increase in insect-repellent components. Notably, metabolites related to plant resistance were significantly enriched in the leaves, especially with a marked upregulation of repellent and insecticidal compounds, which was consistent with the experimental results of the L4 treatment. Additionally, quinic acid was significantly upregulated after pesticide spraying, indicating its key role in protecting Solanum torvum leaves from scale insect damage. This conclusion is consistent with the research on the protective role of quinic acid against western flower thrips on eggplant (Liu et al., 2022). In the long-term co-evolution between plants and insects, plants have developed various adaptive defense mechanisms, one of which is to interfere with the physiological functions of insects by inhibiting the activity of insect detoxifying enzymes (Lu et al., 2004). Exogenous toxins may inhibit the activity of detoxifying enzymes in larvae, leading to the accumulation of toxins in insects and eventually causing death (Ishikawa et al., 2001). Acetylcholinesterase, as the enzyme that hydrolyzes the neurotransmitter acetylcholine, plays an important role in regulating insect nerve function and detoxification (Zhu et al., 2017). This study found that choline, as part of the methyl donor in the metabolic process, decreased after pesticide treatment, which may be related to the use of methamidophos. Therefore, L4 treatment can be considered one of the most suitable measures for newly formed leaves. Unsaturated fatty acids (such as linoleic acid and linolenic acid) also play an important role in plants’ resistance to both biotic and abiotic stresses. They regulate the structure of cell membranes by increasing the unsaturation of membrane lipids, thereby affecting the functionality of membrane lipids (Muqier, 2023). Linolenic acid can be gradually metabolized into jasmonic acid (JA), which plays a key role in regulating plant growth and resistance (Reszczyńska and Hanaka, 2020). Jasmonic acid is an important plant hormone that significantly upregulates its synthesis when plants are subjected to insect feeding or physical damage. It activates downstream signaling pathways, induces the expression of plant defense genes, and ultimately promotes the synthesis of insect-resistant secondary metabolites (Liechti and Farmer, 2002; Lim et al., 2017). In this study, the S4 treatment led to a significant accumulation of linolenic acid and linoleic acid, further confirming their important roles in the plant’s insect resistance mechanism. However, γ-linolenic acid was downregulated in the S2 treatment, indicating that S2 treatment alone may not effectively enhance the plant’s insect resistance. In contrast, in the S4 treatment, due to the synergistic effect of pesticide spraying and leaf plucking, linoleic acid levels increased significantly, with thiamethoxam possibly playing a key role. Moreover, previous studies have shown that the accumulation of unsaturated fatty acids (especially linolenic acid and linoleic acid) is closely related to plants’ resistance to biotic and abiotic stresses. As such, these fatty acids are receiving increasing attention as part of the plant defense system (He et al., 2020). Liu et al.’s research showed that three typical pesticides (the herbicide butachlor, the insecticide chlorpyrifos, and the fungicide triadimenol) induce significant metabolic disturbances in rice, manifesting as a decrease in saturated fatty acids (such as palmitic acid and stearic acid) and an increase in unsaturated fatty acids (such as linolenic acid) (Liu and Zhu, 2020). This result aligns with the changes in soil metabolic characteristics after the S4 treatment in this study. The findings of this study not only provide a theoretical basis for optimizing the insect resistance strategy of *Kandelia obovata* but also offer new insights into the management of soil environments for *Kandelia obovata* growth. However, the insect resistance mechanisms of red mangrove plants other than *Kandelia obovata* still require further exploration. In conclusion, L4 treatment can alleviate pest damage and pesticide residue on *Kandelia obovata* leaves by upregulating quinic acid, while S4 treatment, through the upregulation of phenylalanine, linolenic acid, and linoleic acid, may play a key role in metabolic regulation during insect defense. This study lays the foundation for understanding the insect-resistant metabolic phenotype and defense strategies of *Kandelia obovata* after pesticide treatment and provides important theoretical support for the breeding and molecular improvement of insect-resistant varieties of *Kandelia obovata*.

## Conclusion

The long-term implementation of leaf-picking and pesticide spraying treatments has significantly reduced the damage caused by pests such as scale insects to the leaves of eggplant, while promoting the growth of the plants. This approach has improved the sprouting rate of new branches and the quality of newly developed leaves. Furthermore, the pesticide treatment reshapes the soil and leaf metabolomes, enhancing the chemical properties of the soil and enriching the metabolic products that are beneficial for eggplant growth. It has also reduced the resistance of eggplant to pests and diseases. Among the three treatments—leaf-picking, pesticide spraying, and the combination of both—the leaf-picking and pesticide spraying combination had the most significant impact on the subsequent branches and leaves. Therefore, combining leaf-picking with pesticide spraying offers an effective strategy for managing common pest and disease problems in eggplant cultivation in the future.

## Data Availability

The original contributions presented in the study are included in the article/supplementary material. Further inquiries can be directed to the corresponding authors.
