# Supplementary material for: LC-MS/MS metabolomics analysis: Leaf harvesting plus pesticide spraying can further improve the growth quality of Kandelia obovata Sheue & al
Source: Front Plant Sci. 2025 Sep 10;16:1573160. doi: 10.3389/fpls.2025.1573160 (PMC12462053; doi:10.3389/fpls.2025.1573160)
Supplement: Supplementary file 1 [file Table1.docx]

**Supplementary Table 1.** The list of a total of 1174 identified metabolites in kandelia leaves samples.

| **No.** | **rt(min)** | | **m/z** | | **Name** | | **Adduct** | **Superclass** | | |
| --- | --- | --- | --- | --- | --- | --- | --- | --- | --- | --- |
| 1 | 9.556 | 705.38214 | | 1-O-[(2alpha,3beta,5xi,6beta,9xi,18xi,19alpha)-2,3,6,19,23-pentahydroxy-28-oxoolean-12-en-28-yl]-beta-D-glucopyranose | | [M+Na]+ | | | Lipids and lipid-like molecules |  |
| 2 | 0.64 | 367.13678 | | (-)-Erythro-Anethole glycol 2-glucoside | | [M+Na]+ | | | Organic oxygen compounds |  |
| 3 | 4.839 | 760.2981 | | (-)-Syringaresinol di-O-glucoside | | [M+H]+ | | | Undefined |  |
| 4 | 4.036 | 561.19623 | | (+)-Cycloolivil glucoside | | [M+Na]+ | | | Lignans, neolignans and related compounds |  |
| 5 | 6.783 | 271.0918 | | (+)-Dihydrokawain-5-ol | | [M+Na]+ | | | Phenylpropanoids and polyketides |  |
| 6 | 11.454 | 219.17172 | | (+)-Nootkatone | | [M+H]+ | | | Undefined |  |
| 7 | 10.393 | 313.23206 | | (12Z)-9,10-Dihydroxyoctadec-12-enoic acid | | [M+Na]+ | | | Lipids and lipid-like molecules |  |
| 8 | 4.796 | 651.21521 | | (15S,16Z,17S)-16-ethylidene-4-hydroxy-15-[(2S,3R,4S,5S,6R)-3,4,5-trihydroxy-6-(hydroxymethyl)oxan-2-yl]oxy-2,10,14,20-tetraoxatetracyclo[21.2.2.13,7.012,17]octacosa-1(25),3,5,7(28),12,23,26-heptaene-11,19-dione | | [M+Na]+ | | | Phenylpropanoids and polyketides |  |
| 9 | 9.764 | 317.11163 | | (1E,4E)-1,5-Bis(4-methoxyphenyl)penta-1,4-dien-3-one | | [M+Na]+ | | | Benzenoids |  |
| 10 | 4.985 | 553.22498 | | (1R,2R,3R,3aS,5aS,6R,7R,10R,10aR,10cR)-1,2,6,7-Tetrahydroxy-3,7,10a,10c-tetramethyl-4-oxo-2,3,3a,4,5a,6,6a,7,10,10a,10b,10c-dodecahydro-1H-phenanthro[10,1-bc]furan-10-yl beta-D-glucopyranoside | | [M+Na]+ | | | Lipids and lipid-like molecules |  |
| 11 | 5.361 | 487.21271 | | (1R,2S,4R,5R)-5-Hydroxy-1,7,7-trimethylbicyclo[2.2.1]hept-2-yl 6-O-[(2R,3R,4R)-tetrahydro-3,4-dihydroxy-4-(hydroxymethyl)-2-furanyl]-beta-D-glucopyranoside | | [M+Na]+ | | | Lipids and lipid-like molecules |  |
| 12 | 8.274 | 415.20737 | | (1S,2R,4aR,8aR)-1-Acetoxy-7-isopropylidene-1,4a-dimethyl-6-oxodecahydro-2-naphthalenyl 2,3-dimethyl-2-oxiranecarboxylate | | [M+Na]+ | | | Lipids and lipid-like molecules |  |
| 13 | 5.488 | 609.2243 | | (1S,2S,3R,4S,7R,10S,12R,15S)-4,12-bis(acetyloxy)-1,9,15-trihydroxy-10,14,17,17-tetramethyl-11-oxo-6-oxatetracyclo[11.3.1.0³,¹⁰.0⁴,⁷]heptadec-13-en-2-yl benzoate | | [M+Na]+ | | | Lipids and lipid-like molecules |  |
| 14 | 11.512 | 522.35474 | | (2-Hydroxy-3-octadec-9-enoyloxypropyl) 2-(trimethylazaniumyl)ethyl phosphate | | [M+H]+ | | | Lipids and lipid-like molecules |  |
| 15 | 9.428 | 518.32117 | | (2-Hydroxy-3-octadeca-6,9,12-trienoyloxypropyl) 2-(trimethylazaniumyl)ethyl phosphate | | [M+H]+ | | | Lipids and lipid-like molecules |  |
| 16 | 4.906 | 353.15594 | | (2,6,6-Trimethyl-4-oxo-2-cyclohexen-1-yl)methyl beta-D-glucopyranoside | | [M+Na]+ | | | Organic oxygen compounds |  |
| 17 | 10.831 | 252.23167 | | (2E,4E)-N-(2-Methylpropyl)dodeca-2,4-dienamide | | [M+H]+ | | | Lipids and lipid-like molecules |  |
| 18 | 9.382 | 359.21637 | | (2E,6E,10Z)-12-Hydroxy-10-(hydroxymethyl)-6-methyl-2-(4-methylpent-3-enyl)dodeca-2,6,10-trienoic acid | | [M+Na]+ | | | Lipids and lipid-like molecules |  |
| 19 | 4.192 | 295.07706 | | (2E)-4-[4-(2-Chlorophenyl)piperazin-1-yl]-4-oxobut-2-enoic acid | | [M+H]+ | | | Organoheterocyclic compounds |  |
| 20 | 3.106 | 325.151 | | (2E)-4-{Methyl[3-phenyl-3-(pyridin-2-yl)propyl]amino}-4-oxobut-2-enoic acid | | [M+H]+ | | | Organoheterocyclic compounds |  |
| 21 | 14.405 | 749.51501 | | (2R,3S,4R,5R,8R,10R,11R,13S,14R)-11-[(2S,3R,4S,6R)-4-(Dimethylamino)-3-hydroxy-6-methyloxan-2-yl]oxy-2-ethyl-3,4,10-trihydroxy-13-[(2R,4R,5S,6S)-5-hydroxy-4-methoxy-4,6-dimethyloxan-2-yl]oxy-3,5,6,8,10,12,14-heptamethyl-1-oxa-6-azacyclopentadecan-15-one | | [M+H]+ | | | Organic oxygen compounds |  |
| 22 | 4.977 | 355.16879 | | (2R,3S,4S,5R,6S)-2-(hydroxymethyl)-6-[4-(hydroxymethyl)-1-propan-2-ylcyclohex-3-en-1-yl]oxyoxane-3,4,5-triol | | [M+Na]+ | | | Lipids and lipid-like molecules |  |
| 23 | 12.491 | 293.28021 | | (2R)-1-(5-Hydroxy-3-methylpentyl)-2,5,5,8a-tetramethyldecahydro-2-naphthalenol | | [M-H2O+H]+ | | | Lipids and lipid-like molecules |  |
| 24 | 7.279 | 729.36481 | | (2S,3R,4R,5R,6S)-2-{[(2S,3R,4S,5S)-4,5-Dihydroxy-2-[(1'S,2R,4'S,7'S,8'R,9'S,13'R,14'R,16'S)-7',9',13'-trimethyl-5-methylidene-5'-oxaspiro[oxane-2,6'-pentacyclo[10.8.0.0(2),.0,.0(1)(3),(1)]icosan]-18'-en-14'-oloxy]oxan-3-yl]oxy}-6-methyloxane-3,4,5-triol | | [M+Na]+ | | | Lipids and lipid-like molecules |  |
| 25 | 4.628 | 493.22302 | | (2S,3R,4S,5S,6R)-2-[(2R,3R,4S,5S,6R)-4,5-Dihydroxy-6-(hydroxymethyl)-2-(3-hydroxy-6-methylheptoxy)oxan-3-yl]oxy-6-(hydroxymethyl)oxane-3,4,5-triol | | [M+Na]+ | | | Lipids and lipid-like molecules |  |
| 26 | 5.328 | 765.24829 | | (2S,3R,4S,5S,6R)-2-[4-[(3S,3aR,6S,6aR)-6-[3,5-Dimethoxy-4-[(2S,3R,4S,5S,6R)-3,4,5-trihydroxy-6-(hydroxymethyl)oxan-2-yl]oxyphenyl]-1,3,3a,4,6,6a-hexahydrofuro[3,4-c]furan-3-yl]-2,6-dimethoxyphenoxy]-6-(hydroxymethyl)oxane-3,4,5-triol | | [M+Na]+ | | | Lignans, neolignans and related compounds |  |
| 27 | 9.287 | 487.30566 | | (2S,3R,5R,10R,13R,14S,17R)-17-[(2S,3R)-3,6-Dihydroxy-6-methylheptan-2-yl]-2,3,14-trihydroxy-10,13-dimethyl-2,3,4,5,9,11,12,15,16,17-decahydro-1H-cyclopenta[a]phenanthren-6-one | | [M+Na]+ | | | Lipids and lipid-like molecules |  |
| 28 | 5.948 | 253.10223 | | (2S)-2-[(1S,2R)-1,2-Dihydroxypentyl]-4-methoxy-2,3-dihydropyran-6-one | | [M+Na]+ | | | Organoheterocyclic compounds |  |
| 29 | 3.133 | 433.14914 | | (2S)‐2‐(2‐aminoacetamido)‐3‐(4‐cyanophenyl)‐N‐(9H‐fluoren‐2‐yl)propanamide | | [M+Na]+ | | | Organic acids and derivatives |  |
| 30 | 6.584 | 503.2417 | | (3E)-7-Hydroxy-3,7-dimethyl-3-octen-1-yl 6-O-(6-deoxy-alpha-L-mannopyranosyl)-beta-D-glucopyranoside | | [M+Na]+ | | | Lipids and lipid-like molecules |  |
| 31 | 5.353 | 451.19473 | | (3R,3aR,4S,6S,7aR)-6-ethenyl-4-hydroxy-3,6-dimethyl-7-[(E)-1-[(2R,3R,4S,5S,6R)-3,4,5-trihydroxy-6-(hydroxymethyl)oxan-2-yl]oxyprop-1-en-2-yl]-3,3a,4,5,7,7a-hexahydro-1-benzofuran-2-one | | [M+Na]+ | | | Lipids and lipid-like molecules |  |
| 32 | 13.832 | 333.24008 | | (3R,4R,5R)-3-[(11E)-11,15-Hexadecadien-9-yn-1-yl]-4-hydroxy-5-methyldihydro-2(3H)-furanone | | [M+H]+ | | | Organoheterocyclic compounds |  |
| 33 | 0.799 | 369.11621 | | (4-Hydroxy-3,5-dimethoxyphenyl)methyl-beta-D-glucopyranoside | | [M+Na]+ | | | Organic oxygen compounds |  |
| 34 | 12.199 | 235.1683 | | (4S,5Z,6S)-4-(2-methoxy-2-oxoethyl)-5-[2-[(E)-3-phenylprop-2-enoyl]oxyethylidene]-6-[(2S,3R,4S,5S,6R)-3,4,5-trihydroxy-6-(hydroxymethyl)oxan-2-yl]oxy-4H-pyran-3-carboxylic acid | | [M+H]+ | | | Lipids and lipid-like molecules |  |
| 35 | 3.479 | 437.10474 | | (5-Benzoyloxy-4,6-dihydroxy-3-methoxycyclohexen-1-yl)methyl benzoate | | [M+K]+ | | | Benzenoids |  |
| 36 | 12.134 | 349.2334 | | (5beta,8alpha,9beta,10alpha,13alpha)-18-Methoxy-18-oxokauran-17-oic acid | | [M+H]+ | | | Lipids and lipid-like molecules |  |
| 37 | 5.077 | 249.1104 | | (5S,6S)-4-Methoxy-5-hydroxy-6-phenethyl-5,6-dihydro-2H-pyran-2-one | | [M+H]+ | | | Phenylpropanoids and polyketides |  |
| 38 | 14.344 | 271.23929 | | (5xi,9xi)-Beyer-15-en-18-ol | | [M-H2O+H]+ | | | Lipids and lipid-like molecules |  |
| 39 | 6.596 | 471.21768 | | (6,6-Dimethylbicyclo[3.1.1]hept-2-yl)methyl 6-O-[(2R,3R,4R)-3,4-dihydroxy-4-(hydroxymethyl)tetrahydro-2-furanyl]-beta-D-glucopyranoside | | [M+Na]+ | | | Lipids and lipid-like molecules |  |
| 40 | 13.287 | 427.35446 | | (6beta,22E)-6-Hydroxystigmasta-4,22-dien-3-one | | [M+H]+ | | | Undefined |  |
| 41 | 6.125 | 381.09299 | | (7E,13E)-9,15-Dihydroxy-4,10,16-trimethyl-1,5,11-trioxacyclohexadeca-7,13-diene-2,6,12-trione | | [M+K]+ | | | Phenylpropanoids and polyketides |  |
| 42 | 6.807 | 553.29083 | | (all-E)-beta-Cryptoxanthin | | [M+H]+ | | | Lipids and lipid-like molecules |  |
| 43 | 0.671 | 379.11938 | | (E)-1,4-Bis(4-hydroxy-3-methoxyphenyl)-2,3-dimethylbut-2-ene-1,4-dione | | [M+Na]+ | | | Lignans, neolignans and related compounds |  |
| 44 | 5.372 | 419.18372 | | (E)-3-(4-Methoxyphenyl)-1-[2,4,6-trimethoxy-3-(3-methylbut-2-enyl)phenyl]prop-2-en-1-one | | [M+Na]+ | | | Phenylpropanoids and polyketides |  |
| 45 | 7.772 | 316.18811 | | (E)-Piperolein A | | [M+H]+ | | | Organoheterocyclic compounds |  |
| 46 | 13.321 | 611.46198 | | (S)-7-(((2-O-6-Deoxy-alpha-L-mannopyranosyl)-beta-D-glucopyranosyl)oxy)-2,3-dihydro-5-hydroxy-2-(3-hydroxy-4-methoxyphenyl)-4H-1-benzopyran-4-one | | [M+H]+ | | | Phenylpropanoids and polyketides |  |
| 47 | 3.073 | 167.06892 | | (S)-Suspensaside | | [M+H]+ | | | Organic oxygen compounds |  |
| 48 | 6.292 | 469.20032 | | (Z)-2,6-Dimethyl-7-(4-methyl-5-oxooxolan-2-yl)-3-[[3,4,5-trihydroxy-6-(hydroxymethyl)oxan-2-yl]oxymethyl]hept-5-enoic acid | | [M+Na]+ | | | Lipids and lipid-like molecules |  |
| 49 | 5.686 | 611.24554 | | [(1S,2R,4S,9R,10R,14S,15S,17S)-9-(Furan-3-yl)-1-hydroxy-15-[(1R)-1-hydroxy-2-methoxy-2-oxoethyl]-10,14,16,16-tetramethyl-7,18-dioxo-3,8-dioxapentacyclo[12.3.1.02,4.04,13.05,10]octadecan-17-yl] 2-methylpropanoate | | [M+Na]+ | | | Lipids and lipid-like molecules |  |
| 50 | 4.837 | 443.16745 | | [(1S,3R,7Z,9R,12S,13R,15R)-13-Butan-2-yl-13-hydroxy-3,12-dimethyl-4,11-dioxo-10,14,16-trioxatetracyclo[7.5.1.13,6.012,15]hexadeca-5,7-dien-7-yl]methyl acetate | | [M+Na]+ | | | Organoheterocyclic compounds |  |
| 51 | 8.516 | 715.38416 | | [(2R,3R,4S,5S,6R)-3-Acetyloxy-2-(acetyloxymethyl)-5-hexanoyloxy-6-[(2S,3R)-2,3,4-trihydroxybutoxy]oxan-4-yl] 14-hydroxytetradecanoate | | [M+Na]+ | | | Lipids and lipid-like molecules |  |
| 52 | 2.523 | 496.15527 | | [(2R,3S,4S,5R,6R)-3,4,5-trihydroxy-6-[2-(4-hydroxyphenyl)ethoxy]oxan-2-yl]methyl 2-(2-oxo-1,3-dihydroindol-3-yl)acetate | | [M+Na]+ | | | Lipids and lipid-like molecules |  |
| 53 | 4.591 | 689.2489 | | [(2R,3S,4S,5R,6R)-6-[1,7-Bis(4-hydroxyphenyl)-5-oxoheptan-3-yl]oxy-3,4,5-trihydroxyoxan-2-yl]methyl (E)-3-(3,4-dimethoxyphenyl)prop-2-enoate | | [M+Na]+ | | | Phenylpropanoids and polyketides |  |
| 54 | 5.874 | 519.24023 | | [(2R,3S,4S,5R,6S)-3,4,5-Trihydroxy-6-[(2S,3R,4S,5S,6R)-3,4,5-trihydroxy-6-(hydroxymethyl)oxan-2-yl]oxyoxan-2-yl]methyl 8-methylnonanoate | | [M+Na]+ | | | Lipids and lipid-like molecules |  |
| 55 | 0.662 | 411.14795 | | [(9R,10R)-10-Acetyloxy-8,8-dimethyl-2-oxo-9,10-dihydropyrano[2,3-f]chromen-9-yl] 2-methylbutanoate | | [M+Na]+ | | | Phenylpropanoids and polyketides |  |
| 56 | 3.334 | 427.15579 | | [1-(3,4-Dihydroxy-5-methoxyphenyl)-7-(3,4-dihydroxyphenyl)heptan-3-yl] acetate | | [M+Na]+ | | | Phenylpropanoids and polyketides |  |
| 57 | 8.623 | 432.23477 | | [1,14-Dihydroxy-8-(hydroxymethyl)-4,12,12,15-tetramethyl-5-oxo-13-tetracyclo[8.5.0.02,6.011,13]pentadeca-3,8-dienyl] acetate | | [M+ACN+H]+ | | | Lipids and lipid-like molecules |  |
| 58 | 9.605 | 387.24622 | | [12-Hydroxy-6-(hydroxymethyl)-10-methyl-2-(4-methylpent-3-enyl)dodeca-2,6,10-trienyl] acetate | | [M+Na]+ | | | Lipids and lipid-like molecules |  |
| 59 | 11.411 | 733.41107 | | [17-(2,6-Dihydroxy-6-methyl-3-oxoheptan-2-yl)-3-hydroxy-4,4,9,13,14-pentamethyl-2-[3,4,5-trihydroxy-6-(hydroxymethyl)oxan-2-yl]oxy-2,3,7,8,10,11,12,15,16,17-decahydro-1H-cyclopenta[a]phenanthren-16-yl] acetate | | [M+Na]+ | | | Lipids and lipid-like molecules |  |
| 60 | 3.755 | 549.15936 | | [1a-(Hydroxymethyl)-2-[3,4,5-trihydroxy-6-(hydroxymethyl)oxan-2-yl]oxy-2,5a,6,6a-tetrahydro-1bH-oxireno[5,6]cyclopenta[1,3-c]pyran-6-yl] 3,4-dimethoxybenzoate | | [M+Na]+ | | | Phenylpropanoids and polyketides |  |
| 61 | 7.899 | 729.35815 | | [2-[4-Hydroxy-2,5-bis(hydroxymethyl)-3-(2-methylpropanoyloxy)oxolan-2-yl]oxy-6-(hydroxymethyl)-4,5-bis(2-methylpropanoyloxy)oxan-3-yl] decanoate | | [M+Na]+ | | | Lipids and lipid-like molecules |  |
| 62 | 8.552 | 743.37708 | | [2-[4-Hydroxy-2,5-bis(hydroxymethyl)-3-(3-methylbutanoyloxy)oxolan-2-yl]oxy-6-(hydroxymethyl)-4,5-bis(2-methylpropanoyloxy)oxan-3-yl] decanoate | | [M+Na]+ | | | Lipids and lipid-like molecules |  |
| 63 | 3.869 | 455.1127 | | [3-Hydroxy-1-(4-methoxy-7-oxofuro[3,2-g]chromen-9-yl)oxy-3-methylbutan-2-yl] (E)-2-methylbut-2-enoate | | [M+K]+ | | | Phenylpropanoids and polyketides |  |
| 64 | 11.32 | 707.39929 | | [3,4,5-Trihydroxy-6-(hydroxymethyl)oxan-2-yl] 4,6-dihydroxy-7,12,16-trimethyl-15-(4,5,6-trihydroxy-6-methylheptan-2-yl)pentacyclo[9.7.0.01,3.03,8.012,16]octadecane-7-carboxylate | | [M+Na]+ | | | Lipids and lipid-like molecules |  |
| 65 | 6.387 | 501.16879 | | [3,4,5-Trihydroxy-6-[(3,4,5-trihydroxyoxan-2-yl)oxymethyl]oxan-2-yl] 2,6,6-trimethylcyclohexene-1-carboxylate | | [M+K]+ | | | Organic oxygen compounds |  |
| 66 | 11.545 | 355.28195 | | [4,6-diethyl-6-(4-ethyl-2-methyloctyl)-3H-1,2-dioxin-3-yl]acetic acid | | [M+H]+ | | | Undefined |  |
| 67 | 5.885 | 285.16748 | | [5-Hydroxy-3-(hydroxymethyl)-2-oxo-6-propan-2-ylcyclohex-3-en-1-yl] 3-methylbutanoate | | [M+H]+ | | | Lipids and lipid-like molecules |  |
| 68 | 4.616 | 469.16425 | | [8-[2-(3-Methylbutanoyloxy)propan-2-yl]-2-oxo-8,9-dihydrofuro[2,3-h]chromen-9-yl] 3-methylbutanoate | | [M+K]+ | | | Phenylpropanoids and polyketides |  |
| 69 | 13.375 | 863.49005 | | {6-[2-(hexadecanoyloxy)-3-(icosa-5,8,11,14,17-pentaenoyloxy)propoxy]-3,4,5-trihydroxyoxan-2-yl}methanesulfonic acid | | [M+Na]+ | | | Undefined |  |
| 70 | 2.404 | 136.0751 | | 1-(3-Aminophenyl)ethanone | | [M+H]+ | | | Organic oxygen compounds |  |
| 71 | 1.265 | 351.10751 | | 1-(4-Hydroxyphenyl)-3-[(2R,3R,4S,5S,6R)-3,4,5-trihydroxy-6-(hydroxymethyl)oxan-2-yl]oxypropan-1-one | | [M+Na]+ | | | Lipids and lipid-like molecules |  |
| 72 | 0.802 | 158.07906 | | 1-Acetylproline | | [M+H]+ | | | Organic acids and derivatives |  |
| 73 | 7.793 | 202.21434 | | 1-Decanamine, N,N-dimethyl-, N-oxide | | [M+H]+ | | | Organic nitrogen compounds |  |
| 74 | 11.068 | 284.29346 | | 1-Deoxymethylsphinganine | | [M-H2O+H]+ | | | Organic nitrogen compounds |  |
| 75 | 11.863 | 510.35648 | | 1-Heptadecanoyl-sn-glycero-3-phosphocholine | | [M+H]+ | | | Lipids and lipid-like molecules |  |
| 76 | 11.36 | 496.33804 | | 1-Hexadecanoyl-sn-glycero-3-phosphocholine | | [M+H]+ | | | Lipids and lipid-like molecules |  |
| 77 | 11.553 | 454.29214 | | 1-Hexadecanoyl-sn-glycero-3-phosphoethanolamine | | [M+H]+ | | | Lipids and lipid-like molecules |  |
| 78 | 8.532 | 689.3692 | | 1-O-[(2alpha,3beta,5xi,6beta,9xi,18xi)-2,3,6,23-Tetrahydroxy-28-oxoolean-12-en-28-yl]-beta-D-glucopyranose | | [M+Na]+ | | | Lipids and lipid-like molecules |  |
| 79 | 11.161 | 482.32007 | | 1-Pentadecanoyl-sn-glycero-3-phosphocholine | | [M+H]+ | | | Lipids and lipid-like molecules |  |
| 80 | 12.175 | 524.37109 | | 1-Stearoyl-sn-glycero-3-phosphocholine | | [M+H]+ | | | Lipids and lipid-like molecules |  |
| 81 | 10.22 | 258.27734 | | 1-Tetradecanamine, N,N-dimethyl-, N-oxide | | [M+H]+ | | | Organic nitrogen compounds |  |
| 82 | 4.539 | 113.05785 | | 1,4-Cyclohexanedione | | [M+H]+ | | | Organic oxygen compounds |  |
| 83 | 7.959 | 261.1799 | | 1,9-Heptadecadiene-4,6-diyne-3,8-diol | | [M+H]+ | | | Lipids and lipid-like molecules |  |
| 84 | 6.573 | 295.15112 | | 11-(Phenylcarbonyl)-7,11-diazatricyclo[7.3.1.0<2,7>]trideca-2,4-dien-6-one | | [M+H]+ | | | Organoheterocyclic compounds |  |
| 85 | 6.124 | 399.1969 | | 11,12-Methylenedioxykopsinaline | | [M+H]+ | | | Alkaloids and derivatives |  |
| 86 | 8.575 | 289.17975 | | 12-Hydroxy-13-(hydroxymethyl)-3,5,7-trimethyl-2,4-tetradecadienedioic acid | | [M-3H2O+H]+ | | | Lipids and lipid-like molecules |  |
| 87 | 8.561 | 317.2095 | | 12-Oxopimara-9(11),15-dien-18-oic acid | | [M+H]+ | | | Lipids and lipid-like molecules |  |
| 88 | 12.788 | 337.27225 | | 12,19,20-Trihydroxy-14-methylenegeranylnerol | | [M+H-H2O]+ | | | Undefined |  |
| 89 | 10.915 | 305.24741 | | 13-Hydroxy-7,14-labdadien-6-one | | [M+H]+ | | | Lipids and lipid-like molecules |  |
| 90 | 13.916 | 429.37076 | | 13'-Hydroxy-alpha-tocopherol | | [M+H-H2O]+ | | | Undefined |  |
| 91 | 4.021 | 701.23926 | | 15-(2-Amino-2-oxoethyl)-10,11,23-trihydroxy-18-[(3-methyl-2-oxopentanoyl)amino]-9,14,17-trioxo-N-[(1Z)-1-propen-1-yl]-8,13,16-triazatetracyclo[18.3.1.0~2,7~.0~6,10~]tetracosa-1(24),2,4,6,20,22-hexaene-12-carboxamide | | [M+Na]+ | | | Organic acids and derivatives |  |
| 92 | 11.93 | 529.3808 | | 16-hydroxy-2,2,4a,6a,6b,11,11,14b-octamethyl-4H,4bH,5H,6H,7H,8H,9H,10H,12H,12aH,14H,14aH,15H,16H,16aH-piceno[3,4-d][1,3]dioxine-8a-carboxylic acid | | [M+H]+ | | | Undefined |  |
| 93 | 11.188 | 437.33908 | | 16-Hydroxy-4,4,8,10,14-pentamethyl-17-(4,5,6-trihydroxy-6-methylheptan-2-yl)-1,2,5,6,7,9,11,12,15,16-decahydrocyclopenta[a]phenanthren-3-one | | [M-3H2O+H]+ | | | Lipids and lipid-like molecules |  |
| 94 | 12.959 | 425.37253 | | 17(21)-Hopen-6-one | | [M+H]+ | | | Lipids and lipid-like molecules |  |
| 95 | 11.759 | 541.38074 | | 18-Deoxyleucopaxillone A | | [M+H-H2O]+ | | | Undefined |  |
| 96 | 12.043 | 311.09177 | | 2-(3,4-Dimethoxyphenyl)-7-hydroxy-4H-chromen-4-one | | [M+H]+ | | | Phenylpropanoids and polyketides |  |
| 97 | 4.14 | 533.16119 | | 2-(4-Hydroxyphenyl)ethyl (1S,4aR,7aR)-4a-hydroxy-7-methyl-5-oxo-1-[(2S,3R,4S,5S,6R)-3,4,5-trihydroxy-6-(hydroxymethyl)oxan-2-yl]oxy-1,6,7,7a-tetrahydrocyclopenta[c]pyran-4-carboxylate | | [M+Na]+ | | | Lipids and lipid-like molecules |  |
| 98 | 2.644 | 268.10519 | | 2-(6-Aminopurin-9-yl)-5-(hydroxymethyl)oxolane-3,4-diol | | [M+H]+ | | | Nucleosides, nucleotides, and analogues |  |
| 99 | 5.537 | 227.16222 | | 2-(7-Hydroxy-6-methyloctyl)-2H-furan-5-one | | [M+H]+ | | | Lipids and lipid-like molecules |  |
| 100 | 6.395 | 355.17114 | | 2-(Hydroxymethyl)-6-(6-hydroxy-6-methyl-3-propan-2-ylcyclohex-3-en-1-yl)oxyoxane-3,4,5-triol | | [M+Na]+ | | | Lipids and lipid-like molecules |  |
| 101 | 4.354 | 545.19653 | | 2-(Hydroxymethyl)-6-[5-[3-(hydroxymethyl)-5-(3-hydroxypropyl)-7-methoxy-2,3-dihydro-1-benzofuran-2-yl]-2-methoxyphenoxy]oxane-3,4,5-triol | | [M+Na]+ | | | Phenylpropanoids and polyketides |  |
| 102 | 8.762 | 343.22256 | | 2-[(2'R,8R,8aS)-2',4,4,7,8a-Pentamethylspiro[2,3,4a,5-tetrahydro-1H-naphthalene-8,5'-oxolane]-2'-yl]acetic acid | | [M+Na]+ | | | Lipids and lipid-like molecules |  |
| 103 | 6.107 | 533.25439 | | 2-[(2R,4As,8S,8aS)-8-[2-[(4aS,7R,8aR)-7-(1-carboxyethenyl)-1-hydroxy-4a-methyl-2-oxo-6,7,8,8a-tetrahydro-5H-naphthalen-1-yl]ethyl]-4a-methyl-7-oxo-1,2,3,4,8,8a-hexahydronaphthalen-2-yl]prop-2-enoic acid | | [M+Na]+ | | | Lipids and lipid-like molecules |  |
| 104 | 4.658 | 411.1647 | | 2-[(2Z)-5-(Hexopyranosyloxy)-2-penten-1-yl]-3-oxo-, (1R,2R)-cyclopentaneacetic acid | | [M+Na]+ | | | Lipids and lipid-like molecules |  |
| 105 | 3.367 | 250.14162 | | 2-[(E)-3-(4-Hydroxyphenyl)prop-2-enoyl]oxyethyl-trimethylazanium | | [M]+ | | | Phenylpropanoids and polyketides |  |
| 106 | 5.071 | 600.26465 | | 2-[[5-(4-Hydroxy-3,5-dimethoxyphenyl)-6,7-bis(hydroxymethyl)-1,3-dimethoxy-5,6,7,8-tetrahydronaphthalen-2-yl]oxy]-6-(hydroxymethyl)oxane-3,4,5-triol | | [M+NH4]+ | | | Lignans, neolignans and related compounds |  |
| 107 | 9.479 | 345.23788 | | 2-[2-[(1S,8aR)-1,4,4a,5,6,7,8,8a-Octahydro-2-(hydroxymethyl)-5,5,8a-trimethyl-1-naphthalenyl]ethyl]-, (2Z)-2-butene-1,4-diol | | [M+Na]+ | | | Lipids and lipid-like molecules |  |
| 108 | 4.446 | 561.19421 | | 2-[4-[4-[Hydroxy-(4-hydroxy-3-methoxyphenyl)methyl]-3-(hydroxymethyl)oxolan-2-yl]-2-methoxyphenoxy]-6-(hydroxymethyl)oxane-3,4,5-triol | | [M+Na]+ | | | Lignans, neolignans and related compounds |  |
| 109 | 7.491 | 421.21735 | | 2-[5-[2-[2-[5-(2-Oxopropyl)oxolan-2-yl]propanoyloxy]butyl]oxolan-2-yl]propanoic acid | | [M+Na]+ | | | Organic acids and derivatives |  |
| 110 | 11.352 | 543.3653 | | 2-[7-(acetyloxy)-2-hydroxy-6-(hydroxymethyl)-3a,6,9a,11a-tetramethyl-1H,2H,3H,5H,5aH,7H,8H,9H,11H-cyclopenta[a]phenanthren-1-yl]-6-methyl-5-methylideneheptanoic acid | | [M+H]+ | | | Undefined |  |
| 111 | 3.098 | 498.15182 | | 2-{[6-O-(beta-D-Glucopyranosyl)-beta-D-glucopyranosyl]oxy}-2-phenylacetamide | | [M+Na]+ | | | Lipids and lipid-like molecules |  |
| 112 | 12.489 | 413.26721 | | 2-Acetoxy-4-pentadecylbenzoic acid | | [M+Na]+ | | | Benzenoids |  |
| 113 | 10.233 | 316.28262 | | 2-Aminooctadec-8-ene-1,3,4-triol | | [M+H]+ | | | Organic nitrogen compounds |  |
| 114 | 11.068 | 302.30487 | | 2-Aminooctadecane-1,3-diol | | [M+H]+ | | | Organic nitrogen compounds |  |
| 115 | 8.993 | 318.29977 | | 2-Aminooctadecane-1,3,4-triol | | [M+H]+ | | | Organic nitrogen compounds |  |
| 116 | 2.884 | 205.09683 | | 2-Azaniumyl-3-(1H-indol-3-yl)propanoate | | [M+H]+ | | | Organoheterocyclic compounds |  |
| 117 | 13.665 | 257.22293 | | 2-ethenyl-2,4b-dimethyl-8-methylidene-3,4,4a,5,6,7,8a,9-octahydro-1H-phenanthrene | | [M+H]+ | | | Undefined |  |
| 118 | 3.609 | 137.05766 | | 2-Hydroxyacetophenone | | [M+H]+ | | | Organic oxygen compounds |  |
| 119 | 8.136 | 165.05386 | | 2-Hydroxycinnamic acid | | [M+H]+ | | | Phenylpropanoids and polyketides |  |
| 120 | 4.112 | 561.19366 | | 2-Methoxy-4-[(2R,3R,4S)-tetrahydro-4-hydroxy-4-[(4-hydroxy-3-methoxyphenyl)methyl]-3-(hydroxymethyl)-2-furanyl]phenyl-beta-D-glucopyranoside | | [M+Na]+ | | | Lignans, neolignans and related compounds |  |
| 121 | 5.391 | 327.15805 | | 2-Methoxy-4-[1-hydroxy-2-[2-methoxy-4-(1-propenyl)phenoxy]propyl]phenol | | [M-H2O+H]+ | | | Lignans, neolignans and related compounds |  |
| 122 | 4.362 | 575.18451 | | 2-Methoxy-4-[tetrahydro-3a,6a-dihydroxy-4-(4-hydroxy-3-methoxyphenyl)-1H,3H-furo[3,4-c]furan-1-yl]phenyl-beta-D-glucopyranoside | | [M+Na]+ | | | Lignans, neolignans and related compounds |  |
| 123 | 12.353 | 363.28439 | | 2-Methoxy-4-pentadecylbenzoic acid | | [M+H]+ | | | Benzenoids |  |
| 124 | 2.627 | 311.07486 | | 2-Methyl-3-[(2S,3R,4S,5S,6R)-3,4,5-trihydroxy-6-(hydroxymethyl)oxan-2-yl]oxypyran-4-one | | [M+Na]+ | | | Organic oxygen compounds |  |
| 125 | 5.209 | 179.10863 | | 2-Methylpropyl benzoate | | [M+H]+ | | | Undefined |  |
| 126 | 11.253 | 303.24997 | | 2-Monomyristin | | [M+H]+ | | | Lipids and lipid-like molecules |  |
| 127 | 11.84 | 331.28009 | | 2-Palmitoylglycerol | | [M+H]+ | | | Lipids and lipid-like molecules |  |
| 128 | 4.068 | 136.07356 | | 2-Phenylacetamide | | [M+H]+ | | | Benzenoids |  |
| 129 | 5.046 | 451.15674 | | 2-Phenylethyl 3-O-(4-carboxy-3-hydroxy-3-methylbutanoyl)-beta-D-glucopyranoside | | [M+Na]+ | | | Lipids and lipid-like molecules |  |
| 130 | 3.372 | 439.15808 | | 2-Phenylethyl 6-O-[(2S,3R,4R)-3,4-dihydroxy-4-(hydroxymethyl)tetrahydro-2-furanyl]-beta-D-glucopyranoside | | [M+Na]+ | | | Organic oxygen compounds |  |
| 131 | 2.993 | 478.19077 | | 2‐{5‐[3‐(3‐fluorophenoxy)phenyl]‐1‐(piperidin‐4‐ylmethyl)pyrrole‐2‐carbonyl}pyridine | | [M+Na]+ | | | Benzenoids |  |
| 132 | 3.8 | 379.14709 | | 2,3-Dihydro-2-(1-hydroxy-1-methylethyl)-4-methoxy-6-(2-phenylethyl)-7-benzofurancarboxylic acid | | [M+Na]+ | | | Phenylpropanoids and polyketides |  |
| 133 | 13.96 | 285.23831 | | 2,3-Dihydroxypropyl tetradecanoate | | [M-H2O+H]+ | | | Lipids and lipid-like molecules |  |
| 134 | 7.571 | 395.20239 | | 2,3-Dimethoxystrychnidin-10-one | | [M+H]+ | | | Alkaloids and derivatives |  |
| 135 | 13.411 | 415.35229 | | 2,3-dimethyl-5-(3,7,11,15-tetramethylhexadec-2-en-1-yl)cyclohexa-2,5-diene-1,4-dione | | [M+H]+ | | | Undefined |  |
| 136 | 12.858 | 313.26938 | | 2,4-Dihydroxyheptadecyl acetate | | [M-H2O+H]+ | | | Lipids and lipid-like molecules |  |
| 137 | 8.097 | 519.25488 | | 2,4a,6-Trihydroxy-8-isopropyl-1,1-dimethyl-2,3,4,4a,5,10,11,11a-octahydro-1H-dibenzo[a,d][7]annulen-7-yl beta-D-glucopyranoside | | [M+Na]+ | | | Lipids and lipid-like molecules |  |
| 138 | 3.236 | 122.095 | | 2,6-Dimethylaniline | | [M+H]+ | | | Benzenoids |  |
| 139 | 4.705 | 162.05296 | | 2,8-Quinolinediol | | [M+H]+ | | | Organoheterocyclic compounds |  |
| 140 | 5.379 | 333.05917 | | 2'-Deoxyinosine 5'-monophosphate | | [M+H]+ | | | Nucleosides, nucleotides, and analogues |  |
| 141 | 12.389 | 441.36826 | | 22-Hydoxy-2-hopen-1-one | | [M+H]+ | | | Lipids and lipid-like molecules |  |
| 142 | 10.893 | 443.38824 | | 22-Hydroxy-6-hopanone | | [M+H]+ | | | Lipids and lipid-like molecules |  |
| 143 | 10.796 | 443.38251 | | 22-Hydroxy-7-hopanone | | [M+H]+ | | | Lipids and lipid-like molecules |  |
| 144 | 12.257 | 311.23563 | | 3-(10-Phenyldecyl)Phenol | | [M+H]+ | | | Undefined |  |
| 145 | 8.814 | 787.44794 | | 3-(1H-indol-3-ylmethyl)-6,18-dimethyl-9-(2-methylpropyl)-12-(1-phenylethyl)-15-propan-2-yl-1,4,7,10,13,16,19-heptazacyclotricosane-2,5,8,11,14,17,20-heptone | | [M+H]+ | | | Organic acids and derivatives |  |
| 146 | 3.74 | 167.06902 | | 3-(2-Hydroxyphenyl)propanoic acid | | [M+H]+ | | | Phenylpropanoids and polyketides |  |
| 147 | 5.23 | 353.1167 | | 3-(4-Hydroxyphenyl)propanoic acid 3-(4-hydroxy-3-methoxyphenyl)propyl ester | | [M+Na]+ | | | Benzenoids |  |
| 148 | 10.466 | 721.33472 | | 3-[(2-O-hexopyranosylhexopyranosyl)oxy]-14-hydroxy-, (17E)-card-17-enolide | | [M+Na]+ | | | Lipids and lipid-like molecules |  |
| 149 | 5.759 | 647.15863 | | 3-[(2S,3R,4S,5S,6R)-4,5-Dihydroxy-6-(hydroxymethyl)-3-[(2S,3R,4S,5R,6R)-3,4,5-trihydroxy-6-(hydroxymethyl)oxan-2-yl]oxyoxan-2-yl]oxy-5-hydroxy-2-(4-hydroxyphenyl)-7-methoxychromen-4-one | | [M+Na]+ | | | Phenylpropanoids and polyketides |  |
| 150 | 5.67 | 345.18802 | | 3-[(4As,12aR)-2-methyl-1,3,4,5,12,12a-hexahydropyrido[3,4-b]acridin-4a-yl]phenol | | [M+H]+ | | | Organoheterocyclic compounds |  |
| 151 | 4.743 | 545.19995 | | 3-[[2,3-Dihydro-2-(4-hydroxy-3-methoxyphenyl)-3-(hydroxymethyl)-7-methoxybenzofuran]-5-yl]propyl beta-D-glucopyranoside | | [M+Na]+ | | | Phenylpropanoids and polyketides |  |
| 152 | 6.743 | 589.25232 | | 3-[[6-[[1,4a-Dimethyl-6-methylidene-5-[2-(5-oxo-2H-furan-4-yl)ethyl]-3,4,5,7,8,8a-hexahydro-2H-naphthalen-1-yl]methoxy]-3,4,5-trihydroxyoxan-2-yl]methoxy]-3-oxopropanoic acid | | [M+Na]+ | | | Lipids and lipid-like molecules |  |
| 153 | 3.77 | 381.11499 | | 3-[2-Methoxy-3-[(2S,3R,4S,5S,6R)-3,4,5-trihydroxy-6-(hydroxymethyl)oxan-2-yl]oxyphenyl]propanoic acid | | [M+Na]+ | | | Organic oxygen compounds |  |
| 154 | 1.362 | 367.10202 | | 3-[3-hydroxy-2-[(2S,3R,4S,5S,6R)-3,4,5-trihydroxy-6-(hydroxymethyl)oxan-2-yl]oxyphenyl]propanoic acid | | [M+Na]+ | | | Organic oxygen compounds |  |
| 155 | 12.242 | 269.17432 | | 3-[4-Methyl-1-(2-methylpropanoyl)-3-oxocyclohexyl]butanoic acid | | [M+H]+ | | | Lipids and lipid-like molecules |  |
| 156 | 13.117 | 589.48022 | | 3-{12-hydroxy-12-[5'-(1-hydroxydodecyl)-[2,2'-bioxolan]-5-yl]dodecyl}-5-methyl-5H-furan-2-one | | [M+H-H2O]+ | | | Undefined |  |
| 157 | 13.607 | 573.48218 | | 3-{7-[5-(1-hydroxyhenicosa-4,8-dien-1-yl)oxolan-2-yl]heptyl}-5-methyl-5H-furan-2-one | | [M+H]+ | | | Undefined |  |
| 158 | 13.722 | 547.4682 | | 3-{7-[5-(1-hydroxynonadec-4-en-1-yl)oxolan-2-yl]heptyl}-5-methyl-5H-furan-2-one | | [M+H]+ | | | Undefined |  |
| 159 | 3.602 | 105.03337 | | 3-Cyanopyridine | | [M+H]+ | | | Organoheterocyclic compounds |  |
| 160 | 5.448 | 463.21564 | | 3-Ethyl-4-hydroxy-4-methylpentyl 6-O-[(2S,3R,4R)-tetrahydro-3,4-dihydroxy-4-(hydroxymethyl)-2-furanyl]-beta-D-glucopyranoside | | [M+Na]+ | | | Lipids and lipid-like molecules |  |
| 161 | 3.443 | 385.14227 | | 3-Hydroxy-2-[3-methyl-2-[[3,4,5-trihydroxy-6-(hydroxymethyl)oxan-2-yl]oxymethyl]cyclopent-2-en-1-yl]propanoic acid | | [M+Na]+ | | | Lipids and lipid-like molecules |  |
| 162 | 11.708 | 565.39954 | | 3-Hydroxy-3',4'-didehydro-beta,psi-caroten-4-one | | [M+H]+ | | | Lipids and lipid-like molecules |  |
| 163 | 6.899 | 299.08746 | | 3-Hydroxy-7-methoxy-2-(4-methoxyphenyl)-4H-chromen-4-one | | [M+H]+ | | | Phenylpropanoids and polyketides |  |
| 164 | 3.147 | 165.05428 | | 3-Hydroxycinnamic acid | | [M+H]+ | | | Phenylpropanoids and polyketides |  |
| 165 | 2.222 | 153.0531 | | 3-Hydroxyphenylacetic acid | | [M+H]+ | | | Benzenoids |  |
| 166 | 4.843 | 383.1445 | | 3-Methoxy-4-[1-(3,4,5-trimethoxyphenyl)propan-2-yloxy]benzaldehyde | | [M+Na]+ | | | Benzenoids |  |
| 167 | 1.003 | 150.07594 | | 3-Methyladenine | | [M+H]+ | | | Organoheterocyclic compounds |  |
| 168 | 4.704 | 148.07417 | | 3-Methyloxindole | | [M+H]+ | | | Organoheterocyclic compounds |  |
| 169 | 2.964 | 485.16428 | | 3,4-Dimethoxyphenyl 6-O-(6-deoxy-alpha-L-mannopyranosyl)-hexopyranoside | | [M+Na]+ | | | Organic oxygen compounds |  |
| 170 | 12.661 | 459.35849 | | 3,5,5-trimethyl-4-(3,7,12,16,18-pentamethylnonadeca-1,3,5,7,9,11,13,15-octaen-1-yl)cyclohex-2-en-1-one | | [M+H]+ | | | Undefined |  |
| 171 | 7.091 | 527.24304 | | 3,5,5-Trimethyl-4-[3-[(6-O-beta-D-xylopyranosyl-beta-D-glucopyranosyl)oxy]butyl]-2-cyclohexen-1-one | | [M+Na]+ | | | Lipids and lipid-like molecules |  |
| 172 | 7.314 | 397.21695 | | 3,5,5-Trimethyl-4-[3-[(beta-D-glucopyranosyl)oxy]butyl]-3-cyclohexen-1-ol | | [M+Na]+ | | | Lipids and lipid-like molecules |  |
| 173 | 4.536 | 387.19702 | | 3,5,5-Trimethyl-4beta-hydroxy-4-[3-(beta-D-glucopyranosyloxy)-1-butenyl]-2-cyclohexene-1-one | | [M+H]+ | | | Lipids and lipid-like molecules |  |
| 174 | 9.281 | 291.13559 | | 3,6-Diphenylcyclohexan-1,2-diol | | [M+Na]+ | | | Organic oxygen compounds |  |
| 175 | 7.158 | 315.15518 | | 3,7-Dimethyl-10-phenylphenazin-10-ium-2,8-diamine | | [M]+ | | | Organoheterocyclic compounds |  |
| 176 | 7.547 | 977.48175 | | 3a-[3,4-Dihydroxy-6-[[3,4,5-trihydroxy-6-(hydroxymethyl)oxan-2-yl]oxymethyl]-5-(3,4,5-trihydroxy-6-methyloxan-2-yl)oxyoxan-2-yl]oxycarbonyl-5b,8,8,11a-tetramethyl-9-oxo-1-prop-1-en-2-yl-2,3,4,5,6,7,7a,10,11,11b,12,13,13a,13b-tetradecahydro-1H-cyclopenta[a]chrysene-5a-carboxylic acid | | [M+Na]+ | | | Lipids and lipid-like molecules |  |
| 177 | 7.756 | 493.24179 | | 3beta-HYDROXYDEOXODIHYDRODEOXYGEDUNIN | | [M+Na]+ | | | Organoheterocyclic compounds |  |
| 178 | 7.978 | 393.12973 | | 4-(1,3-Benzodioxol-5-ylmethyl)-3-[(3,4-dimethoxyphenyl)methyl]oxolan-2-one | | [M+Na]+ | | | Lignans, neolignans and related compounds |  |
| 179 | 5.034 | 305.09851 | | 4-(2,3-Dihydroxy-3-methylbutoxy)furo(3,2-g)chromen-7-one | | [M+H]+ | | | Phenylpropanoids and polyketides |  |
| 180 | 5.685 | 617.14447 | | 4-(3,4-Dihydroxyphenyl)-5-[(6-O-beta-D-xylopyranosyl-beta-D-glucopyranosyl)oxy]-7-methoxycoumarin | | [M+Na]+ | | | Phenylpropanoids and polyketides |  |
| 181 | 6.435 | 435.16098 | | 4-[(2R,3R,4S,5S,6R)-3,4,5-trihydroxy-6-(hydroxymethyl)oxan-2-yl]oxypentan-2-yl (E)-3-(4-hydroxyphenyl)prop-2-enoate | | [M+Na]+ | | | Lipids and lipid-like molecules |  |
| 182 | 10.917 | 409.27026 | | 4-[10-(5-hydroxy-3,5,6-trimethyl-2,6-dihydropyran-2-yl)undeca-1,3,5,7,9-pentaen-1-yl]-2,4,5-trimethylcyclopent-2-en-1-one | | [M+H]+ | | | Undefined |  |
| 183 | 7.862 | 465.27814 | | 4-[3-Methyl-5-(5,6,7-trihydroxy-1,2,4a,5-tetramethyl-3,4,6,7,8,8a-hexahydro-2H-naphthalen-1-yl)pentoxy]-4-oxobutanoic acid | | [M+Na]+ | | | Lipids and lipid-like molecules |  |
| 184 | 4.636 | 599.17773 | | 4-[3,4,5-Trihydroxy-6-(hydroxymethyl)oxan-2-yl]oxy-9-(3,4,5-trimethoxyphenyl)-5a,6,8a,9-tetrahydro-5H-[2]benzofuro[6,5-f][1,3]benzodioxol-8-one | | [M+Na]+ | | | Lignans, neolignans and related compounds |  |
| 185 | 5.387 | 409.17465 | | 4-[4-(beta-D-Glucopyranosyloxy)-2-hydroxy-2,6,6-trimethylcyclohexylidene]-3-buten-2-one | | [M+Na]+ | | | Lipids and lipid-like molecules |  |
| 186 | 11.076 | 478.28723 | | 4-[5-[[4-[5-[Acetyl(hydroxy)amino]pentylamino]-4-oxobutanoyl]-hydroxyamino]pentylamino]-4-oxobutanoic acid | | [M+NH4]+ | | | Lipids and lipid-like molecules |  |
| 187 | 2.672 | 303.13281 | | 4-[Formyl(6-methoxyquinolin-8-yl)amino]pentanoic acid | | [M+H]+ | | | Organoheterocyclic compounds |  |
| 188 | 9.809 | 381.20728 | | 4-Hydroxy-3-[(2E,6E)-4-hydroxy-3,7,11-trimethyldodeca-2,6,10-trienyl]benzoic acid | | [M+Na]+ | | | Lipids and lipid-like molecules |  |
| 189 | 4.052 | 179.06744 | | 4-Hydroxy-3-methoxycinnamaldehyde | | [M+H]+ | | | Benzenoids |  |
| 190 | 2.999 | 429.20535 | | 4-Hydroxy-6-methyl-3-methylidene-5-[5-[(2R,3R,4S,5S,6R)-3,4,5-trihydroxy-6-(hydroxymethyl)oxan-2-yl]oxypentan-2-yl]-3a,4,7,7a-tetrahydro-1-benzofuran-2-one | | [M+H]+ | | | Lipids and lipid-like molecules |  |
| 191 | 2.874 | 165.05568 | | 4-Hydroxycinnamic acid | | [M+H]+ | | | Phenylpropanoids and polyketides |  |
| 192 | 5.835 | 193.05016 | | 4-Methylesculetin | | [M+H]+ | | | Phenylpropanoids and polyketides |  |
| 193 | 7.341 | 525.30127 | | 4,4,8,10,14-Pentamethyl-17-(4,5,6-trihydroxy-6-methylheptan-2-yl)-2,5,6,7,9,15-hexahydro-1H-cyclopenta[a]phenanthrene-3,16-dione | | [M+K]+ | | | Lipids and lipid-like molecules |  |
| 194 | 11.17 | 393.27719 | | 4,9,13-trimethyl-15-(2,6,6-trimethylcyclohex-1-en-1-yl)pentadeca-2,4,6,8,10,12,14-heptaenoic acid | | [M+H]+ | | | Undefined |  |
| 195 | 6.314 | 453.17151 | | 4'-O-b-D-Glucosyl-5-O-methylvisamminol | | [M+H]+ | | | Organoheterocyclic compounds |  |
| 196 | 6.871 | 331.08151 | | 4',5,7-Trihydroxy-3,6-dimethoxyflavone | | [M+H]+ | | | Phenylpropanoids and polyketides |  |
| 197 | 12.571 | 351.24841 | | 5-(5-Methoxycarbonyl-5,8a-dimethyl-2-methylidene-3,4,4a,6,7,8-hexahydro-1H-naphthalen-1-yl)-3-methylpentanoic acid | | [M+H]+ | | | Lipids and lipid-like molecules |  |
| 198 | 6.731 | 209.15102 | | 5-(6-Methyl-6-hydroxyoctyl)furan-2(5H)-one | | [M-H2O+H]+ | | | Organoheterocyclic compounds |  |
| 199 | 4.537 | 225.14656 | | 5-(6-Methyl-7-oxooctyl)furan-2(5H)-one | | [M+H]+ | | | Organoheterocyclic compounds |  |
| 200 | 4.477 | 467.1875 | | 5-[(1R,3S,5R,8S)-3-(beta-D-glucopyranosyloxy)-8-hydroxy-1,5-dimethyl-6-oxabicyclo[3.2.1]oct-8-yl]-3-methyl-, (2Z,4E)-2,4-pentadienoic acid | | [M+Na]+ | | | Lipids and lipid-like molecules |  |
| 201 | 12.492 | 332.29022 | | 5-[(1S,4aS,8aS)-Decahydro-5,5,8a-trimethyl-2-methylene-1-naphthalenyl]-3-methyl-, (2E)-2-penten-1-ol | | [M+ACN+H]+ | | | Lipids and lipid-like molecules |  |
| 202 | 8.443 | 693.34766 | | 5-[(3S,8R,10R,13R,14S,17R)-3-[(2R,3R,4R,5S,6R)-3,4-Dihydroxy-6-(hydroxymethyl)-5-[(2S,3R,4R,5R,6S)-3,4,5-trihydroxy-6-methyloxan-2-yl]oxyoxan-2-yl]oxy-14-hydroxy-10,13-dimethyl-1,2,3,6,7,8,9,11,12,15,16,17-dodecahydrocyclopenta[a]phenanthren-17-yl]pyran-2-one | | [M+H]+ | | | Lipids and lipid-like molecules |  |
| 203 | 4.24 | 623.19598 | | 5-[[4,5-Dihydroxy-6-(hydroxymethyl)-3-[(3,4,5-trihydroxy-2-oxanyl)oxy]-2-oxanyl]oxy]-7,8-dimethoxy-3-(4-methoxyphenyl)-1-benzopyran-4-one | | [M+H]+ | | | Phenylpropanoids and polyketides |  |
| 204 | 7.65 | 401.22791 | | 5-[5-(Acetyloxymethyl)-1,2,4a-trimethyl-7-oxo-3,4,8,8a-tetrahydro-2H-naphthalen-1-yl]-3-methylpentanoic acid | | [M+Na]+ | | | Lipids and lipid-like molecules |  |
| 205 | 9.159 | 283.20612 | | 5-[5-Hydroxy-3-(hydroxymethyl)pentyl]-8a-(hydroxymethyl)-5,6-dimethyl-3,4,4a,6,7,8-hexahydronaphthalene-1-carboxylic acid | | [M-4H2O+H]+ | | | Lipids and lipid-like molecules |  |
| 206 | 1.09 | 423.08743 | | 5-Chloro-3-[(E)-3,4-dihydroxy-3,5-dimethylhept-1-enyl]-7,8-dihydroxy-7-methyl-8H-isochromen-6-one | | [M+K]+ | | | Organoheterocyclic compounds |  |
| 207 | 8.23 | 237.23248 | | 5-Cyclohexadecen-1-one | | [M+H]+ | | | Organic oxygen compounds |  |
| 208 | 13.263 | 263.23602 | | 5-dodecyl-4-hydroxy-4-methylcyclopent-2-en-1-one | | [M+H-H2O]+ | | | Undefined |  |
| 209 | 5.543 | 163.07298 | | 5-Hydroxy-1-tetralone | | [M+H]+ | | | Undefined |  |
| 210 | 5.475 | 699.2254 | | 5-Hydroxy-2-(4-methoxyphenyl)-8-(3-methylbut-2-enyl)-7-[3,4,5-trihydroxy-6-(hydroxymethyl)oxan-2-yl]oxy-3-(3,4,5-trihydroxy-6-methyloxan-2-yl)oxychromen-4-one | | [M+Na]+ | | | Lignans, neolignans and related compounds |  |
| 211 | 8.459 | 219.17172 | | 5-hydroxyculmorin | | [M-2H2O+H]+ | | | Lipids and lipid-like molecules |  |
| 212 | 0.67 | 127.03866 | | 5-Hydroxymethylfurfural | | [M+H]+ | | | Undefined |  |
| 213 | 4.442 | 455.15195 | | 5‐(1‐benzofuran‐2‐yl)‐N,N‐dimethyl‐2‐[4‐(piperazin‐1‐yl)phenyl]‐1,3‐thiazole‐4‐carboxamide | | [M+Na]+ | | | Organoheterocyclic compounds |  |
| 214 | 11.138 | 375.25391 | | 5,5,9,13-tetramethyl-7-(phenylmethylidene)tetracyclo[11.2.1.01,10.04,9]hexadec-14-en-6-one | | [M+H]+ | | | Undefined |  |
| 215 | 5.435 | 273.07303 | | 5,7-Dihydroxy-2-(4-hydroxyphenyl)chroman-4-one | | [M+H]+ | | | Phenylpropanoids and polyketides |  |
| 216 | 5.935 | 449.10507 | | 5,7-Dihydroxy-2-[4-hydroxy-3-[(2S,3R,4S,5R)-3,4,5-trihydroxyoxan-2-yl]oxyphenyl]-3-methoxychromen-4-one | | [M+H]+ | | | Phenylpropanoids and polyketides |  |
| 217 | 6.368 | 257.07889 | | 5,7-Dihydroxyflavanone | | [M+H]+ | | | Phenylpropanoids and polyketides |  |
| 218 | 5.963 | 307.10498 | | 5,7-Dimethoxyflavanone | | [M+Na]+ | | | Phenylpropanoids and polyketides |  |
| 219 | 13.738 | 255.20958 | | 5\|A-Androst-1-ene-3\|A,17\|A-diol | | [M-2H2O+H]+ | | | Lipids and lipid-like molecules |  |
| 220 | 6.203 | 453.20703 | | 6-(beta-D-Glucopyranosyloxy)-4,5,6,7,8,8a-hexahydro-5-hydroxy-5-(1-hydroxy-1-methylethyl)-3,8-dimethyl-2(1H)-azulenone | | [M+Na]+ | | | Lipids and lipid-like molecules |  |
| 221 | 4.982 | 493.16431 | | 6-[2-Hydroxy-3-methyl-3-[(2S,3R,4S,5S,6R)-3,4,5-trihydroxy-6-(hydroxymethyl)oxan-2-yl]oxybutyl]-5,7-dimethoxychromen-2-one | | [M+Na]+ | | | Lipids and lipid-like molecules |  |
| 222 | 8.935 | 415.21045 | | 6-[3-[(3,4-Dimethoxyphenyl)methyl]-4-methoxy-2-(methoxymethyl)butyl]-4-methoxy-1,3-benzodioxole | | [M-H2O+H]+ | | | Lignans, neolignans and related compounds |  |
| 223 | 13.94 | 397.38147 | | 6-{9a,11a-dimethyl-1H,2H,3H,3aH,3bH,4H,8H,9H,9bH,10H,11H-cyclopenta[a]phenanthren-1-yl}-3-ethyl-2-methylheptane | | [M+H]+ | | | Undefined |  |
| 224 | 5.785 | 677.16687 | | 6-Hydroxy-3-[3-hydroxy-4-[3,4,5-trihydroxy-6-[[3,4,5-trihydroxy-6-(hydroxymethyl)oxan-2-yl]oxymethyl]oxan-2-yl]oxyphenyl]-5,7-dimethoxychromen-4-one | | [M+Na]+ | | | Phenylpropanoids and polyketides |  |
| 225 | 1.997 | 239.06982 | | 6-Hydroxyflavone | | [M+H]+ | | | Phenylpropanoids and polyketides |  |
| 226 | 10.807 | 321.24136 | | 6-Oxocativic acid | | [M+H]+ | | | Lipids and lipid-like molecules |  |
| 227 | 14.204 | 405.25946 | | 6,7-Diketolithocholic acid | | [M+H]+ | | | Lipids and lipid-like molecules |  |
| 228 | 10.214 | 206.11511 | | 6,7-Dimethoxy-1-methyl-3,4-dihydroisoquinoline | | [M+H]+ | | | Organoheterocyclic compounds |  |
| 229 | 3.492 | 407.09344 | | 6,7-Dimethoxy-8-(beta-D-glucopyranosyloxy)-2H-1-benzopyran-2-one | | [M+Na]+ | | | Phenylpropanoids and polyketides |  |
| 230 | 4.935 | 719.21869 | | 6'-O-P-Coumaroylgenipin gentiobioside | | [M+Na]+ | | | Lipids and lipid-like molecules |  |
| 231 | 5.461 | 351.12268 | | 6a,7,8-Trihydroxy-3-methyl-3,4,5,6,7,12a-hexahydro-2H-benzo[a]anthracene-1,12-dione | | [M+Na]+ | | | Benzenoids |  |
| 232 | 7.229 | 393.14883 | | 7-Ethyl-10-Hydroxy-Camptothecin | | [M+H]+ | | | Undefined |  |
| 233 | 4.895 | 435.12906 | | 7-Hydroxy-2-(4-hydroxyphenyl)-5-[(2S,3R,4S,5S,6R)-3,4,5-trihydroxy-6-(hydroxymethyl)oxan-2-yl]oxy-2,3-dihydrochromen-4-one | | [M+H]+ | | | Phenylpropanoids and polyketides |  |
| 234 | 7.105 | 507.28619 | | 7-Hydroxy-4-[(3Z)-5-hydroxy-3-methyl-3-penten-1-yl]-4a,8,8-trimethyl-3-methylenedecahydro-2-naphthalenyl beta-D-mannopyranoside | | [M+Na]+ | | | Lipids and lipid-like molecules |  |
| 235 | 6.506 | 415.21857 | | 7-Hydroxymitragynine | | [M+H]+ | | | Alkaloids and derivatives |  |
| 236 | 12.121 | 401.33948 | | 7-Ketocholesterol | | [M+H]+ | | | Lipids and lipid-like molecules |  |
| 237 | 5.415 | 373.07648 | | 7,10-Dihydroxyspiro[11-oxatricyclo[4.4.1.01,6]undeca-3,8-diene-5,3'-2,4-dioxatricyclo[7.3.1.05,13]trideca-1(12),5,7,9(13),10-pentaene]-2-one | | [M+Na]+ | | | Benzenoids |  |
| 238 | 7.71 | 376.25845 | | 7''-ethyl-6-methyl-4'',7''-dihydro-3''H-dispiro[oxane-2,6'-[7,9,12]triazatricyclo[6.3.1.04,12]dodecane-10',2''-oxepin]-8'-en-3-ol | | [M+H]+ | | | Undefined |  |
| 239 | 4.434 | 425.17639 | | 7(S)-Chloro-7-deoxylincomycin | | [M+H]+ | | | Organic acids and derivatives |  |
| 240 | 8.131 | 425.13 | | 8-(2,3-Dihydroxy-3-methylbutyl)-5,7-dimethoxy-2-phenyl-2,3-dihydrochromen-4-one | | [M+K]+ | | | Phenylpropanoids and polyketides |  |
| 241 | 8.235 | 293.20847 | | 8-(4-Oxo-5-pent-2-enylcyclopent-2-en-1-yl)octanoic acid | | [M+H]+ | | | Lipids and lipid-like molecules |  |
| 242 | 9.732 | 293.21082 | | 8-[3-Oxo-2-[(E)-pent-2-enyl]cyclopenten-1-yl]octanoic acid | | [M+H]+ | | | Lipids and lipid-like molecules |  |
| 243 | 11.698 | 349.27341 | | 8-[5-(Acetyloxy)-3-methylpentyl]octahydro-4,4,8a-trimethyl-7-methylene-2(1H)-naphthalenone | | [M+H]+ | | | Lipids and lipid-like molecules |  |
| 244 | 4.509 | 399.17786 | | 8-Hydroxy-2,7,7,11,15-pentamethyl-5,12,16-trioxapentacyclo[9.8.0.0(2),.0,.0(1)(3),(1)]nonadec-13(18)-ene-3,17-dione | | [M+Na]+ | | | Organoheterocyclic compounds |  |
| 245 | 12.115 | 261.21771 | | 8-Hydroxy-9,10-epoxystearic acid | | [M-3H2O+H]+ | | | Lipids and lipid-like molecules |  |
| 246 | 4.258 | 559.18677 | | 8-Hydroxypinoresinol 4'-glucoside | | [M+Na]+ | | | Lignans, neolignans and related compounds |  |
| 247 | 0.644 | 305.12003 | | 8,8-Dimethyl-2-phenyl-4H,8H-benzo[1,2-b:3,4-b']dipyran-4-one | | [M+H]+ | | | Phenylpropanoids and polyketides |  |
| 248 | 4.529 | 209.15292 | | 9-Epiblumenol B | | [M-H2O+H]+ | | | Lipids and lipid-like molecules |  |
| 249 | 5.221 | 659.16168 | | 9-Methoxy-7-[4-[3,4,5-trihydroxy-6-[[3,4,5-trihydroxy-6-(hydroxymethyl)oxan-2-yl]oxymethyl]oxan-2-yl]oxyphenyl]-[1,3]dioxolo[4,5-g]chromen-8-one | | [M+Na]+ | | | Phenylpropanoids and polyketides |  |
| 250 | 6.241 | 379.13309 | | 9-Methoxycamptothecin | | [M+H]+ | | | Alkaloids and derivatives |  |
| 251 | 2.611 | 394.09027 | | 9-Nitro-20(S)-camptothecin | | [M+H]+ | | | Alkaloids and derivatives |  |
| 252 | 11.897 | 282.28046 | | 9-Octadecenamide | | [M+H]+ | | | Lipids and lipid-like molecules |  |
| 253 | 4.594 | 121.06442 | | Acetophenone | | [M+CH3OH+H]+ | | | Undefined |  |
| 254 | 6.262 | 693.30707 | | ACETYL ISOGAMBOGIC ACID | | [M+Na]+ | | | Undefined |  |
| 255 | 11.837 | 427.30206 | | Acetyl-Leu-Leu-Arg-al | | [M+H]+ | | | Organic acids and derivatives |  |
| 256 | 6.879 | 535.211 | | Aerucyclamide A | | [M+H]+ | | | Organic acids and derivatives |  |
| 257 | 5.67 | 331.07413 | | Aflatoxin G2 | | [M+H]+ | | | Phenylpropanoids and polyketides |  |
| 258 | 5.691 | 433.10812 | | Afzelin | | [M+H]+ | | | Phenylpropanoids and polyketides |  |
| 259 | 4.925 | 399.14188 | | Ailanthone | | [M+Na]+ | | | Lipids and lipid-like molecules |  |
| 260 | 3.482 | 371.12796 | | Ajugol | | [M+H]+ | | | Undefined |  |
| 261 | 4.316 | 373.12354 | | Alantrypinone | | [M+H]+ | | | Organoheterocyclic compounds |  |
| 262 | 3.942 | 503.15112 | | Albiflorin | | [M+Na]+ | | | Lipids and lipid-like molecules |  |
| 263 | 6.003 | 417.26337 | | Alfentanil | | [M+H]+ | | | Benzenoids |  |
| 264 | 9.428 | 513.34857 | | Alisol A | | [M+H]+ | | | Lipids and lipid-like molecules |  |
| 265 | 9.651 | 495.34149 | | Alisol B | | [M+Na]+ | | | Lipids and lipid-like molecules |  |
| 266 | 14.412 | 429.36783 | | All-rac-3,4-Dehydro-alpha-tocopherol | | [M+H]+ | | | Undefined |  |
| 267 | 2.203 | 363.12451 | | alpha-Aminoorcein | | [M+H]+ | | | Organoheterocyclic compounds |  |
| 268 | 11.556 | 457.36914 | | alpha-Boswellic acid | | [M+H]+ | | | Lipids and lipid-like molecules |  |
| 269 | 2.392 | 449.16467 | | alpha-D-Glucopyranoside, alpha-D-glucopyranosyl, 3-(2-methylbutanoate) | | [M+Na]+ | | | Lipids and lipid-like molecules |  |
| 270 | 0.662 | 217.07005 | | alpha-D-Glucopyranoside, methyl | | [M+Na]+ | | | Organic oxygen compounds |  |
| 271 | 1.311 | 273.09201 | | alpha-Naphthoflavone | | [M+H]+ | | | Phenylpropanoids and polyketides |  |
| 272 | 10.803 | 868.50397 | | alpha-Solanine | | [M+H]+ | | | Lipids and lipid-like molecules |  |
| 273 | 12.575 | 395.36362 | | alpha-Spinasterol | | [M+H-H2O]+ | | | Lipids and lipid-like molecules |  |
| 274 | 7.422 | 351.21463 | | Alteichin | | [M+H]+ | | | Benzenoids |  |
| 275 | 6.047 | 433.181 | | Alternaric acid | | [M+Na]+ | | | Lipids and lipid-like molecules |  |
| 276 | 4.766 | 455.18427 | | Amethopterin | | [M+H]+ | | | Organoheterocyclic compounds |  |
| 277 | 3.306 | 370.18427 | | Amisulpride | | [M+H]+ | | | Benzenoids |  |
| 278 | 6.617 | 265.18488 | | Ammothamnine | | [M+H]+ | | | Undefined |  |
| 279 | 2.406 | 163.08511 | | Anabasine | | [M+H]+ | | | Alkaloids and derivatives |  |
| 280 | 0.838 | 515.11517 | | Andrographidin B | | [M+Na]+ | | | Phenylpropanoids and polyketides |  |
| 281 | 7.121 | 587.33557 | | Anthranoyllycoctonine | | [M+H]+ | | | Lipids and lipid-like molecules |  |
| 282 | 3.435 | 557.18317 | | Arctiin | | [M+Na]+ | | | Lignans, neolignans and related compounds |  |
| 283 | 5.212 | 247.13115 | | Arglabin | | [M+H]+ | | | Undefined |  |
| 284 | 7.479 | 294.07684 | | Aristolactam I | | [M+H]+ | | | Undefined |  |
| 285 | 3.474 | 389.12451 | | Artemetin | | [M+H]+ | | | Undefined |  |
| 286 | 11.363 | 283.15155 | | Artemisinin | | [M+H]+ | | | Lipids and lipid-like molecules |  |
| 287 | 2.909 | 181.04837 | | Aspirin | | [M+H]+ | | | Benzenoids |  |
| 288 | 4.274 | 443.14996 | | Asteltoxin C | | [M+K]+ | | | Organoheterocyclic compounds |  |
| 289 | 5.691 | 449.10553 | | Astragalin | | [M+H]+ | | | Phenylpropanoids and polyketides |  |
| 290 | 8.672 | 727.37982 | | Atazanavir | | [M+Na]+ | | | Organic acids and derivatives |  |
| 291 | 9.638 | 321.14612 | | Auraptene | | [M+Na]+ | | | Lipids and lipid-like molecules |  |
| 292 | 10.842 | 311.25772 | | Avocadene 2-acetate | | [M-H2O+H]+ | | | Lipids and lipid-like molecules |  |
| 293 | 4.597 | 107.04828 | | Benzaldehyde | | [M-C6H10O4+H]+ | | | Benzenoids |  |
| 294 | 3.602 | 79.05345 | | Benzene | | [M-C6H10O5+H]+ | | | Benzenoids |  |
| 295 | 9.541 | 304.29654 | | Benzododecinium | | [M]+ | | | Benzenoids |  |
| 296 | 7.838 | 183.08047 | | Benzophenone | | [M+H]+ | | | Benzenoids |  |
| 297 | 4.901 | 590.289 | | Benzoylmesaconine | | [M+H]+ | | | Undefined |  |
| 298 | 3.874 | 455.15515 | | Benzyl gentiobioside | | [M+Na]+ | | | Organic oxygen compounds |  |
| 299 | 12.04 | 427.38983 | | beta-Amyrin | | [M+H]+ | | | Lipids and lipid-like molecules |  |
| 300 | 11.562 | 823.4798 | | beta-D-Glucopyranoside, (3beta,5xi,6alpha,9xi,12beta)-20-(beta-D-glucopyranosyloxy)-3,12-dihydroxydammar-24-en-6-yl | | [M+Na]+ | | | Lipids and lipid-like molecules |  |
| 301 | 0.626 | 118.08549 | | Betaine | | [M+H]+ | | | Organic acids and derivatives |  |
| 302 | 13.359 | 443.38541 | | Betulin | | [M+H]+ | | | Lipids and lipid-like molecules |  |
| 303 | 9.866 | 349.21063 | | Bexarotene | | [M+H]+ | | | Lipids and lipid-like molecules |  |
| 304 | 12.661 | 533.40912 | | Biochanin A 7-O-beta-d-glucoside-6''-O-malonate | | [M+H]+ | | | Phenylpropanoids and polyketides |  |
| 305 | 9.786 | 434.26807 | | Brachystamide B | | [M+Na]+ | | | Organoheterocyclic compounds |  |
| 306 | 6.823 | 281.17126 | | Brefeldin A | | [M+H]+ | | | Phenylpropanoids and polyketides |  |
| 307 | 4.717 | 433.14325 | | Bruceine D | | [M+Na]+ | | | Lipids and lipid-like molecules |  |
| 308 | 6.902 | 395.20264 | | Brucine | | [M+H]+ | | | Alkaloids and derivatives |  |
| 309 | 11.35 | 497.35553 | | Bryodulcosigenin | | [M+Na]+ | | | Lipids and lipid-like molecules |  |
| 310 | 4.18 | 881.30548 | | Bussein | | [M+Na]+ | | | Undefined |  |
| 311 | 5.753 | 318.09805 | | Buxifoliadine-H | | [M+H]+ | | | Organoheterocyclic compounds |  |
| 312 | 10.196 | 317.20682 | | Cafestol | | [M+H]+ | | | Organoheterocyclic compounds |  |
| 313 | 13.748 | 383.36432 | | Campesterol | | [M+H-H2O]+ | | | Lipids and lipid-like molecules |  |
| 314 | 5.965 | 153.1261 | | Camphor | | [M+H]+ | | | Lipids and lipid-like molecules |  |
| 315 | 9.44 | 379.14975 | | Carinatone | | [M+Na]+ | | | Phenylpropanoids and polyketides |  |
| 316 | 3.363 | 375.11057 | | Casticin | | [M+H]+ | | | Phenylpropanoids and polyketides |  |
| 317 | 14.402 | 607.26392 | | Cepharanthine | | [M+H]+ | | | Lignans, neolignans and related compounds |  |
| 318 | 7.659 | 551.26025 | | Chaetoglobosin A | | [M+Na]+ | | | Alkaloids and derivatives |  |
| 319 | 5.937 | 529.25269 | | Chaetoglobosin C | | [M+H]+ | | | Alkaloids and derivatives |  |
| 320 | 3.982 | 433.14639 | | Chaetoviridin A | | [M+H]+ | | | Organic oxygen compounds |  |
| 321 | 12.348 | 893.55847 | | Chlorophyll a from Chlorella | | [M]+ | | | Organoheterocyclic compounds |  |
| 322 | 11.099 | 907.46051 | | Chlorophyll b from Chlorella | | [M]+ | | | Organoheterocyclic compounds |  |
| 323 | 12.355 | 383.32812 | | Cholest-4,6-Dien-3-One | | [M+H]+ | | | Lipids and lipid-like molecules |  |
| 324 | 7.69 | 409.21658 | | Cholic acid | | [M+H]+ | | | Lipids and lipid-like molecules |  |
| 325 | 0.638 | 104.1063 | | Choline | | [M]+ | | | Organic nitrogen compounds |  |
| 326 | 6.902 | 301.06656 | | Chrysoeriol | | [M+H]+ | | | Phenylpropanoids and polyketides |  |
| 327 | 5.022 | 307.11249 | | Cimifugin | | [M+H]+ | | | Organoheterocyclic compounds |  |
| 328 | 7.447 | 295.18707 | | Cinchonine | | [M+H]+ | | | Undefined |  |
| 329 | 6.526 | 133.10013 | | Cinnamaldehyde | | [M+H]+ | | | Phenylpropanoids and polyketides |  |
| 330 | 5.064 | 477.13528 | | Cirsimarin | | [M+H]+ | | | Phenylpropanoids and polyketides |  |
| 331 | 9.09 | 223.2067 | | cis-Nerolidol | | [M+H]+ | | | Lipids and lipid-like molecules |  |
| 332 | 5.704 | 325.1586 | | Citalopram | | [M+H]+ | | | Benzenoids |  |
| 333 | 3.317 | 251.08817 | | Citrinin | | [M+H]+ | | | Organoheterocyclic compounds |  |
| 334 | 3.694 | 207.06177 | | Citropten | | [M+H]+ | | | Phenylpropanoids and polyketides |  |
| 335 | 13.151 | 251.19858 | | Clareolide | | [M+H]+ | | | Undefined |  |
| 336 | 6.917 | 347.23007 | | Corticosterone | | [M+H]+ | | | Lipids and lipid-like molecules |  |
| 337 | 13.775 | 363.21527 | | CORTISOL | | [M+H]+ | | | Undefined |  |
| 338 | 6.169 | 361.19861 | | Cortisone | | [M+H]+ | | | Lipids and lipid-like molecules |  |
| 339 | 5.527 | 383.20499 | | Corynoxeine | | [M+H]+ | | | Undefined |  |
| 340 | 6.525 | 233.14961 | | Costunolide | | [M+H]+ | | | Lipids and lipid-like molecules |  |
| 341 | 3.147 | 147.04298 | | Coumarin | | [M+H]+ | | | Phenylpropanoids and polyketides |  |
| 342 | 12.542 | 599.40259 | | Crassostreaxanthin B | | [M+H]+ | | | Lipids and lipid-like molecules |  |
| 343 | 4.107 | 447.16187 | | Crepidiaside B | | [M+Na]+ | | | Organic oxygen compounds |  |
| 344 | 11.179 | 539.29297 | | Cucurbitacin D | | [M+Na]+ | | | Lipids and lipid-like molecules |  |
| 345 | 1.903 | 489.13373 | | Curculigoside | | [M+H]+ | | | Undefined |  |
| 346 | 9.03 | 581.32629 | | Cyanidin 3-O-beta-D-sambubioside | | [M]+ | | | Phenylpropanoids and polyketides |  |
| 347 | 5.564 | 595.16412 | | Cyanidin 3-O-rutinoside | | [M]+ | | | Phenylpropanoids and polyketides |  |
| 348 | 7.614 | 697.33685 | | Cyanidin-3-(6''-malonylglucoside) | | [M+NH4]+ | | | Undefined |  |
| 349 | 13.354 | 306.27802 | | Cyclic cmp | | [M+H]+ | | | Organic oxygen compounds |  |
| 350 | 5.704 | 523.20905 | | cyclo[DL-Ala-DL-Trp-DL-Ala-DL-Val-Gly] | | [M+K]+ | | | Organic acids and derivatives |  |
| 351 | 10.408 | 513.34857 | | Cycloastragenol | | [M+H]+ | | | Undefined |  |
| 352 | 4.468 | 393.20465 | | Cylindrol B | | [M+Na]+ | | | Organic oxygen compounds |  |
| 353 | 8.687 | 292.12097 | | Cyproconazole | | [M+H]+ | | | Benzenoids |  |
| 354 | 0.986 | 266.07068 | | Cytidine | | [M+Na]+ | | | Nucleosides, nucleotides, and analogues |  |
| 355 | 6.48 | 489.23212 | | Cytidine 5'-diphosphocholine | | [M+H]+ | | | Nucleosides, nucleotides, and analogues |  |
| 356 | 7.184 | 161.09499 | | D-Alanyl-D-alanine | | [M+H]+ | | | Organic acids and derivatives |  |
| 357 | 3.9 | 179.02977 | | Daphnetin | | [M+H]+ | | | Phenylpropanoids and polyketides |  |
| 358 | 4.917 | 481.25552 | | Decaethylene glycol | | [M+Na]+ | | | Organic oxygen compounds |  |
| 359 | 3.333 | 374.11905 | | Deferasirox | | [M+H]+ | | | Organoheterocyclic compounds |  |
| 360 | 11.165 | 300.22681 | | Dehydroabietamide | | [M+H]+ | | | Lipids and lipid-like molecules |  |
| 361 | 13.7 | 451.35605 | | Dehydroeburicoic acid | | [M+H-H2O]+ | | | Undefined |  |
| 362 | 1.261 | 302.11981 | | Dehydroevodiamine | | [M+H]+ | | | Organoheterocyclic compounds |  |
| 363 | 3.505 | 322.12314 | | Dehydrohistidyltryptophyldiketopiperazine | | [M+H]+ | | | Organic acids and derivatives |  |
| 364 | 11.662 | 585.3739 | | Dehydroxynocardamine | | [M+H]+ | | | Phenylpropanoids and polyketides |  |
| 365 | 4.718 | 465.09885 | | Delphinidin 3-glucoside | | [M]+ | | | Phenylpropanoids and polyketides |  |
| 366 | 3.501 | 465.09616 | | Delphinidin-3-O-glucoside chloride | | [M]+ | | | Phenylpropanoids and polyketides |  |
| 367 | 5.82 | 213.18323 | | delta-Tridecalactone | | [M+H]+ | | | Undefined |  |
| 368 | 5.707 | 639.1864 | | Demethoxycentaureidin 7-O-rutinoside | | [M+H]+ | | | Phenylpropanoids and polyketides |  |
| 369 | 4.001 | 389.13 | | Demethylnobiletin | | [M+H]+ | | | Phenylpropanoids and polyketides |  |
| 370 | 11.252 | 325.23361 | | Denatonium | | [M]+ | | | Organic acids and derivatives |  |
| 371 | 13.897 | 593.27081 | | Deoxykhivorin | | [M+Na]+ | | | Organoheterocyclic compounds |  |
| 372 | 6.056 | 247.13142 | | Desacetoxymatricarin | | [M+H]+ | | | Organoheterocyclic compounds |  |
| 373 | 4.803 | 323.10339 | | Desmedipham | | [M+Na]+ | | | Benzenoids |  |
| 374 | 5.79 | 251.16101 | | Desmethylmianserin | | [M+H]+ | | | Organoheterocyclic compounds |  |
| 375 | 5.39 | 207.13815 | | Dexibuprofen | | [M+H]+ | | | Phenylpropanoids and polyketides |  |
| 376 | 11.571 | 583.40912 | | Diadinochrome A | | [M+H]+ | | | Lipids and lipid-like molecules |  |
| 377 | 4.765 | 605.21863 | | Diadinoxanthin A | | [M+Na]+ | | | Lipids and lipid-like molecules |  |
| 378 | 3.285 | 261.12784 | | Diaveridine | | [M+H]+ | | | Benzenoids |  |
| 379 | 9.64 | 279.15536 | | Dibutyl phthalate | | [M+H]+ | | | Benzenoids |  |
| 380 | 5.409 | 182.18875 | | Dicyclohexylamine | | [M+H]+ | | | Organic nitrogen compounds |  |
| 381 | 7.111 | 192.13844 | | Diethyltoluamide | | [M+H]+ | | | Benzenoids |  |
| 382 | 5.485 | 397.12234 | | Diffractaic acid | | [M+Na]+ | | | Phenylpropanoids and polyketides |  |
| 383 | 3.472 | 395.07202 | | Diflufenican | | [M+H]+ | | | Benzenoids |  |
| 384 | 0.83 | 287.10995 | | Dihydro-3-methylene-4-[(2E)-3-methyl-4-(tetrahydro-4-methyl-5-oxo-2-furanyl)-2-buten-1-yl]-, (4R)-2(3H)-furanone, | | [M+H-H2O]+ | | | Lipids and lipid-like molecules |  |
| 385 | 6.203 | 559.30292 | | Dihydrocelastryl diacetate | | [M+Na]+ | | | Benzenoids |  |
| 386 | 6.089 | 302.17139 | | Dihydrocodeine | | [M+H]+ | | | Alkaloids and derivatives |  |
| 387 | 12.485 | 391.2807 | | Dioctyl phthalate | | [M+H]+ | | | Benzenoids |  |
| 388 | 9.674 | 891.46637 | | Dioscin | | [M+Na]+ | | | Lipids and lipid-like molecules |  |
| 389 | 13.044 | 415.31519 | | Diosgenin | | [M+H]+ | | | Lipids and lipid-like molecules |  |
| 390 | 10.916 | 251.04523 | | Diphenyl phosphate | | [M+H]+ | | | Organic acids and derivatives |  |
| 391 | 13.683 | 282.27557 | | Dodemorph | | [M+H]+ | | | Organoheterocyclic compounds |  |
| 392 | 2.382 | 154.0844 | | Dopamine | | [M+H]+ | | | Benzenoids |  |
| 393 | 2.19 | 296.14648 | | Dosulepin | | [M+H]+ | | | Organoheterocyclic compounds |  |
| 394 | 10.054 | 318.23953 | | Drofenine | | [M+H]+ | | | Benzenoids |  |
| 395 | 7.845 | 237.15916 | | Dropropizine | | [M+H]+ | | | Organoheterocyclic compounds |  |
| 396 | 9.779 | 503.29462 | | Ecdysterone | | [M+Na]+ | | | Lipids and lipid-like molecules |  |
| 397 | 5.899 | 414.21811 | | Echimidine N-oxide | | [M+H]+ | | | Alkaloids and derivatives |  |
| 398 | 13.423 | 551.42316 | | Echinenone | | [M+H]+ | | | Lipids and lipid-like molecules |  |
| 399 | 7.678 | 247.12886 | | Ellipticine | | [M+H]+ | | | Organoheterocyclic compounds |  |
| 400 | 9.893 | 327.23163 | | Ent-17-Hydroxy-16beta-kauran-19-al | | [M+Na]+ | | | Lipids and lipid-like molecules |  |
| 401 | 3.247 | 308.10928 | | Ergonovine | | [M-H2O+H]+ | | | Alkaloids and derivatives |  |
| 402 | 13.047 | 338.34024 | | Erucamide | | [M+H]+ | | | Lipids and lipid-like molecules |  |
| 403 | 3.986 | 350.15839 | | Erucifoline | | [M+H]+ | | | Alkaloids and derivatives |  |
| 404 | 5.359 | 413.17358 | | Erysubin F | | [M+Na]+ | | | Phenylpropanoids and polyketides |  |
| 405 | 1.546 | 238.10748 | | Ethopabate | | [M+H]+ | | | Benzenoids |  |
| 406 | 4.06 | 197.08057 | | Ethyl vanillate | | [M+H]+ | | | Benzenoids |  |
| 407 | 7.425 | 194.11615 | | Ethyl-4-dimethylaminobenzoate | | [M+H]+ | | | Benzenoids |  |
| 408 | 13.793 | 409.38095 | | Euphol | | [M+H-H2O]+ | | | Undefined |  |
| 409 | 9.21 | 615.35297 | | Exemestane | | [M+H]+ | | | Lipids and lipid-like molecules |  |
| 410 | 2.46 | 336.10623 | | Fenamiphos sulfone | | [M+H]+ | | | Benzenoids |  |
| 411 | 2.706 | 300.08389 | | Fenbendazole | | [M+H]+ | | | Organoheterocyclic compounds |  |
| 412 | 5.48 | 319.06729 | | Fenofibric acid | | [M+H]+ | | | Benzenoids |  |
| 413 | 11.895 | 304.25998 | | Fenpropimorph | | [M+H]+ | | | Benzenoids |  |
| 414 | 5.648 | 535.10962 | | Flavone base + 4O, O-MalonylHex | | [M+H]+ | | | Phenylpropanoids and polyketides |  |
| 415 | 4.31 | 415.13367 | | Flecainide | | [M+H]+ | | | Benzenoids |  |
| 416 | 0.801 | 310.13785 | | Fluoxetine | | [M+H]+ | | | Benzenoids |  |
| 417 | 1.733 | 334.12302 | | Flurtamone | | [M+H]+ | | | Benzenoids |  |
| 418 | 6.015 | 433.22455 | | Forskolin | | [M+H]+ | | | Lipids and lipid-like molecules |  |
| 419 | 5.913 | 427.16998 | | Fragransin B2 | | [M+Na]+ | | | Lignans, neolignans and related compounds |  |
| 420 | 4.904 | 223.06003 | | Fraxidin | | [M+H]+ | | | Phenylpropanoids and polyketides |  |
| 421 | 0.618 | 203.05374 | | Fructose (Generic Ketohexose) | | [M+Na]+ | | | Organic oxygen compounds |  |
| 422 | 6.244 | 393.17712 | | Furaquinocin C | | [M+Na]+ | | | Organoheterocyclic compounds |  |
| 423 | 0.673 | 141.0545 | | Furfuryl acetate | | [M+H-2H2O]+ | | | Undefined |  |
| 424 | 7.42 | 288.15042 | | Galantamin | | [M+H]+ | | | Alkaloids and derivatives |  |
| 425 | 7.665 | 273.18201 | | Galaxolidone | | [M+H]+ | | | Organoheterocyclic compounds |  |
| 426 | 8.85 | 403.24323 | | Gamabufotalin | | [M+H]+ | | | Undefined |  |
| 427 | 7.421 | 373.19342 | | gamma-(Acetyloxy)-3-oxo-2-(2-penten-1-yl)-1-cyclopentene-1-octanoic acid | | [M+Na]+ | | | Lipids and lipid-like molecules |  |
| 428 | 4.519 | 411.19598 | | gamma-Tocotrienol | | [M+H]+ | | | Lipids and lipid-like molecules |  |
| 429 | 11.289 | 545.38251 | | Ganoderic acid Mi | | [M+H]+ | | | Undefined |  |
| 430 | 7.063 | 413.21051 | | Gardneramine | | [M+H]+ | | | Organoheterocyclic compounds |  |
| 431 | 0.665 | 411.12253 | | Geniposide | | [M+H]+ | | | Undefined |  |
| 432 | 3.927 | 397.11127 | | Geniposidic acid | | [M+Na]+ | | | Lipids and lipid-like molecules |  |
| 433 | 5.274 | 169.11955 | | Geranic acid | | [M+H]+ | | | Lipids and lipid-like molecules |  |
| 434 | 12.663 | 219.17336 | | Germacrone | | [M+H]+ | | | Undefined |  |
| 435 | 3.313 | 441.13196 | | Ginkgolide C | | [M+H]+ | | | Undefined |  |
| 436 | 13.697 | 789.47833 | | Ginsenoside Rk1 | | [M+H]+ | | | Undefined |  |
| 437 | 3.324 | 515.15118 | | Globularin | | [M+Na]+ | | | Organic oxygen compounds |  |
| 438 | 11.369 | 357.30069 | | Glyceryl monooleate | | [M+H]+ | | | Lipids and lipid-like molecules |  |
| 439 | 12.454 | 359.31369 | | Glyceryl monostearate | | [M+H]+ | | | Lipids and lipid-like molecules |  |
| 440 | 11.969 | 348.31073 | | Glyceryl palmitate | | [M+NH4]+ | | | Lipids and lipid-like molecules |  |
| 441 | 4.704 | 130.02541 | | Goitrin | | [M+H]+ | | | Organoheterocyclic compounds |  |
| 442 | 8.561 | 669.34491 | | Goshonoside F5 | | [M+Na]+ | | | Lipids and lipid-like molecules |  |
| 443 | 1.54 | 152.05634 | | Guanine | | [M+H]+ | | | Organoheterocyclic compounds |  |
| 444 | 9.714 | 307.22504 | | h_61_17_Epioxandrolone | | [M+H]+ | | | Lipids and lipid-like molecules |  |
| 445 | 7.337 | 291.19547 | | h_92_Oxabolone | | [M+H]+ | | | Lipids and lipid-like molecules |  |
| 446 | 4.567 | 523.1391 | | Haploperoside C | | [M+Na]+ | | | Phenylpropanoids and polyketides |  |
| 447 | 4.643 | 777.22021 | | Hedysarimcoumestan D | | [M+Na]+ | | | Phenylpropanoids and polyketides |  |
| 448 | 12.783 | 303.047 | | Herbacetin | | [M+H]+ | | | Phenylpropanoids and polyketides |  |
| 449 | 3.916 | 413.21115 | | Hexa(methoxymethyl)melamine | | [M+Na]+ | | | Organic nitrogen compounds |  |
| 450 | 4.356 | 513.19592 | | Hexacyclinic acid | | [M+Na]+ | | | Lipids and lipid-like molecules |  |
| 451 | 3.765 | 250.14151 | | Hexahomomethionine S-oxide | | [M+H]+ | | | Organic acids and derivatives |  |
| 452 | 10.6 | 346.27072 | | Himbacine | | [M+H]+ | | | Organoheterocyclic compounds |  |
| 453 | 8.761 | 349.13959 | | Histidine-betaxanthin | | [M+H]+ | | | Undefined |  |
| 454 | 3.644 | 169.04839 | | Homogentisic acid | | [M+H]+ | | | Benzenoids |  |
| 455 | 7.697 | 363.21231 | | Hydrocortisone | | [M+H]+ | | | Lipids and lipid-like molecules |  |
| 456 | 0.69 | 124.0383 | | Hydron;pyridine-3-carboxylate | | [M+H]+ | | | Organoheterocyclic compounds |  |
| 457 | 6.168 | 327.21109 | | Hydroquinidine | | [M+H]+ | | | Undefined |  |
| 458 | 4.84 | 111.04352 | | Hydroquinone | | [M+H]+ | | | Benzenoids |  |
| 459 | 3.88 | 529.14301 | | Hypocrellin B | | [M+H]+ | | | Undefined |  |
| 460 | 5.013 | 389.21292 | | Icariside B5 | | [M+H]+ | | | Lipids and lipid-like molecules |  |
| 461 | 8.898 | 425.13275 | | Icariside F2 | | [M+Na]+ | | | Organic oxygen compounds |  |
| 462 | 5.157 | 429.18866 | | Ilicicolin C | | [M+Na]+ | | | Organic oxygen compounds |  |
| 463 | 4.181 | 349.18182 | | Imidocarb | | [M+H]+ | | | Benzenoids |  |
| 464 | 2.882 | 146.05907 | | Indole-3-carboxaldehyde | | [M+H]+ | | | Organoheterocyclic compounds |  |
| 465 | 2.402 | 134.05891 | | Indoxyl | | [M+H-H2O]+ | | | Organoheterocyclic compounds |  |
| 466 | 2.608 | 134.05882 | | Indoxyl sulfate | | [M+H]+ | | | Organic acids and derivatives |  |
| 467 | 5.03 | 403.13608 | | Irigenin trimethyl ether | | [M+H]+ | | | Phenylpropanoids and polyketides |  |
| 468 | 4.081 | 148.03842 | | Isatin | | [M+H]+ | | | Organoheterocyclic compounds |  |
| 469 | 6.287 | 233.15179 | | Isoalantolactone | | [M+H]+ | | | Lipids and lipid-like molecules |  |
| 470 | 6.012 | 411.17273 | | Isoapetalic acid | | [M+Na]+ | | | Organoheterocyclic compounds |  |
| 471 | 14.037 | 838.62805 | | Isobutyryl CoA | | [M+H]+ | | | Lipids and lipid-like molecules |  |
| 472 | 3.458 | 195.06409 | | Isoferulic acid | | [M+H]+ | | | Phenylpropanoids and polyketides |  |
| 473 | 9.215 | 551.27869 | | Isogomphrenin I | | [M+H]+ | | | Organic oxygen compounds |  |
| 474 | 7.443 | 653.31195 | | Isohernandezine | | [M+H]+ | | | Phenylpropanoids and polyketides |  |
| 475 | 4.444 | 429.16782 | | Isolupalbigenin | | [M+Na]+ | | | Phenylpropanoids and polyketides |  |
| 476 | 3.983 | 377.08664 | | Isomarticin | | [M+H]+ | | | Phenylpropanoids and polyketides |  |
| 477 | 12.655 | 285.21677 | | Isonerylgeraniol-18-oic acid | | [M-2H2O+H]+ | | | Lipids and lipid-like molecules |  |
| 478 | 6.295 | 139.1109 | | Isophorone | | [M+H]+ | | | Organic oxygen compounds |  |
| 479 | 5.866 | 179.11598 | | Isoproturon-didemethyl | | [M+H]+ | | | Benzenoids |  |
| 480 | 5.757 | 479.11847 | | Isorhamnetin-3-O-glucoside | | [M+H]+ | | | Phenylpropanoids and polyketides |  |
| 481 | 5.508 | 443.13205 | | Isorhapontin | | [M+Na]+ | | | Phenylpropanoids and polyketides |  |
| 482 | 4.766 | 193.04803 | | Isoscopoletin | | [M+H]+ | | | Undefined |  |
| 483 | 6.15 | 359.20593 | | Itopride | | [M+H]+ | | | Benzenoids |  |
| 484 | 4.045 | 417.07483 | | Jamaicin | | [M+K]+ | | | Phenylpropanoids and polyketides |  |
| 485 | 4.881 | 619.16229 | | Jaslanceoside A | | [M+Na]+ | | | Lipids and lipid-like molecules |  |
| 486 | 6.194 | 181.12003 | | Jasmolone | | [M+H]+ | | | Undefined |  |
| 487 | 2.135 | 383.12827 | | Junipediol A 8-glucoside | | [M+Na]+ | | | Organic oxygen compounds |  |
| 488 | 5.362 | 381.11481 | | Junipediol B 8-O-glucoside | | [M+Na]+ | | | Organic oxygen compounds |  |
| 489 | 5.5 | 603.12543 | | Kaemoferol 5-Xyl(1,2)Glc | | [M+Na]+ | | | Phenylpropanoids and polyketides |  |
| 490 | 5.692 | 595.16516 | | Kaempferol 3-glucorhamnoside | | [M+H]+ | | | Undefined |  |
| 491 | 2.739 | 236.08841 | | Kainic Acid | | [M+Na]+ | | | Organic acids and derivatives |  |
| 492 | 11.962 | 722.45087 | | Khasianine | | [M+H]+ | | | Undefined |  |
| 493 | 3.49 | 409.1098 | | Khelloside | | [M+H]+ | | | Organoheterocyclic compounds |  |
| 494 | 0.632 | 439.14062 | | Kotanin | | [M+H]+ | | | Phenylpropanoids and polyketides |  |
| 495 | 6.354 | 783.28333 | | Kuwanone H | | [M+Na]+ | | | Phenylpropanoids and polyketides |  |
| 496 | 13.123 | 613.47717 | | L-Glutathione (oxidized form) | | [M+H]+ | | | Organic acids and derivatives |  |
| 497 | 0.803 | 132.09966 | | L-Norleucine | | [M+H]+ | | | Organic acids and derivatives |  |
| 498 | 0.61 | 130.04947 | | L-Pyroglutamic acid | | [M+H]+ | | | Organic acids and derivatives |  |
| 499 | 8.077 | 727.3421 | | Lampranthin II | | [M+H]+ | | | Alkaloids and derivatives |  |
| 500 | 5.009 | 412.22458 | | Lasiocarpine | | [M+H]+ | | | Alkaloids and derivatives |  |
| 501 | 9.436 | 343.29242 | | Lauramidopropylbetaine | | [M]+ | | | Organic acids and derivatives |  |
| 502 | 4.426 | 427.19254 | | Leupeptin | | [M+H]+ | | | Organic acids and derivatives |  |
| 503 | 14.107 | 191.10432 | | Ligustilide | | [M+H]+ | | | Organoheterocyclic compounds |  |
| 504 | 5.75 | 347.07419 | | Limocitrin | | [M+H]+ | | | Phenylpropanoids and polyketides |  |
| 505 | 12.515 | 307.26178 | | Linolenic acid ethyl ester | | [M+H]+ | | | Undefined |  |
| 506 | 0.877 | 330.11694 | | Lithosprmoside | | [M+H]+ | | | Undefined |  |
| 507 | 0.846 | 259.04276 | | Lofexidine | | [M+H]+ | | | Benzenoids |  |
| 508 | 6.244 | 197.11588 | | Loliolide | | [M+H]+ | | | Organoheterocyclic compounds |  |
| 509 | 12.662 | 551.42316 | | Lutein | | [M+H-H2O]+ | | | Lipids and lipid-like molecules |  |
| 510 | 4.342 | 449.10553 | | Luteolin-4'-o-glucoside | | [M+H]+ | | | Phenylpropanoids and polyketides |  |
| 511 | 5.023 | 513.17688 | | Lyalosidic acid | | [M+H]+ | | | Lipids and lipid-like molecules |  |
| 512 | 4.888 | 570.25391 | | Lyoniside | | [M+NH4]+ | | | Lignans, neolignans and related compounds |  |
| 513 | 2.601 | 116.04732 | | Maleamic acid | | [M+H]+ | | | Lipids and lipid-like molecules |  |
| 514 | 5.777 | 655.1853 | | Malvin | | [M]+ | | | Phenylpropanoids and polyketides |  |
| 515 | 3.025 | 296.11472 | | Mebendazole | | [M+H]+ | | | Benzenoids |  |
| 516 | 4.848 | 178.12172 | | Mephedrone | | [M+H]+ | | | Organic oxygen compounds |  |
| 517 | 7.78 | 234.18564 | | Meptazinol | | [M+H]+ | | | Organoheterocyclic compounds |  |
| 518 | 1.231 | 150.05389 | | Methionine | | [M+H]+ | | | Organic acids and derivatives |  |
| 519 | 3.655 | 443.15274 | | Methyl (1R,4aS,8S,8aS)-3-methoxy-1-methyl-8-[(2S,3R,4S,5S,6R)-3,4,5-trihydroxy-6-(hydroxymethyl)oxan-2-yl]oxy-1,3,4,4a,8,8a-hexahydropyrano[3,4-c]pyran-5-carboxylate | | [M+Na]+ | | | Organic oxygen compounds |  |
| 520 | 5.453 | 503.15344 | | Methyl (3R,4R,5R)-5-[(2R,3R,4S,5S,6R)-4,5-dihydroxy-6-(hydroxymethyl)-3-[(E)-3-phenylprop-2-enoyl]oxyoxan-2-yl]oxy-3,4-dihydroxycyclohexene-1-carboxylate | | [M+Na]+ | | | Lipids and lipid-like molecules |  |
| 521 | 4.446 | 605.17419 | | Methyl (4S,5Z,6S)-5-(2-acetyloxyethylidene)-4-[2-[2-(4-hydroxyphenyl)ethoxy]-2-oxoethyl]-6-[(2S,3R,4S,5S,6R)-3,4,5-trihydroxy-6-(hydroxymethyl)oxan-2-yl]oxy-4H-pyran-3-carboxylate | | [M+Na]+ | | | Lipids and lipid-like molecules |  |
| 522 | 4.222 | 563.17078 | | Methyl (4S,5Z,6S)-5-(2-hydroxyethylidene)-4-[2-[2-(4-hydroxyphenyl)ethoxy]-2-oxoethyl]-6-[(2S,3R,4S,5S,6R)-3,4,5-trihydroxy-6-(hydroxymethyl)oxan-2-yl]oxy-4H-pyran-3-carboxylate | | [M+Na]+ | | | Lipids and lipid-like molecules |  |
| 523 | 6.063 | 187.07329 | | Methyl 1-naphthoate | | [M+H]+ | | | Benzenoids |  |
| 524 | 13.233 | 551.49847 | | Methyl 18-(hexadecanoyloxy)octadec-9-enoate | | [M+H]+ | | | Undefined |  |
| 525 | 3.759 | 403.13531 | | Methyl 2-(4,5-dihydroxy-6-methoxy-3,6-dimethyl-2-oxocyclohex-3-en-1-yl)oxy-4-hydroxy-3,6-dimethylbenzoate | | [M+Na]+ | | | Benzenoids |  |
| 526 | 4.027 | 387.10632 | | Methyl 2-(9,10a-dihydroxy-4a-methoxy-1-methyl-5,10-dioxo-3,4-dihydro-1H-benzo[g]isochromen-3-yl)acetate | | [M+Na]+ | | | Organoheterocyclic compounds |  |
| 527 | 5.423 | 535.23962 | | Methyl 2-[(1R,5R,6R,13S,14S,16S)-14-acetyloxy-6-(furan-3-yl)-1,5,15,15-tetramethyl-8,17-dioxo-7-oxatetracyclo[11.3.1.02,11.05,10]heptadec-10-en-16-yl]acetate | | [M+Na]+ | | | Lipids and lipid-like molecules |  |
| 528 | 3.073 | 385.08472 | | Methyl 2-hydroxy-6-(4-hydroxy-2-methoxy-6-methoxycarbonylphenoxy)-4-methylbenzoate | | [M+Na]+ | | | Benzenoids |  |
| 529 | 1.179 | 179.06938 | | Methyl 4-hydroxycinnamate | | [M+H]+ | | | Undefined |  |
| 530 | 5.389 | 225.14842 | | Methyl jasmonate | | [M+H]+ | | | Lipids and lipid-like molecules |  |
| 531 | 0.614 | 266.15967 | | Mirtazapine | | [M+H]+ | | | Organoheterocyclic compounds |  |
| 532 | 8.534 | 693.40295 | | Monensin | | [M+Na]+ | | | Lipids and lipid-like molecules |  |
| 533 | 6.103 | 223.0957 | | Monoisobutylphthalic Acid | | [M+H]+ | | | Benzenoids |  |
| 534 | 3.266 | 421.1662 | | Morusin | | [M+H]+ | | | Undefined |  |
| 535 | 4.038 | 435.1814 | | Mundulone | | [M+H]+ | | | Phenylpropanoids and polyketides |  |
| 536 | 4.37 | 477.19315 | | Mundulone acetate | | [M+H]+ | | | Phenylpropanoids and polyketides |  |
| 537 | 2.559 | 289.12515 | | Myclobutanil | | [M+H]+ | | | Phenylpropanoids and polyketides |  |
| 538 | 4.844 | 319.04718 | | Myricetin | | [M+H]+ | | | Phenylpropanoids and polyketides |  |
| 539 | 3.776 | 193.08517 | | Myristicin | | [M+H]+ | | | Organoheterocyclic compounds |  |
| 540 | 11.285 | 356.35178 | | N-(2-Hydroxyethyl)icosanamide | | [M+H]+ | | | Organic nitrogen compounds |  |
| 541 | 5.101 | 379.17178 | | N-(Naphthalen-1-yl)-1-pentyl-1H-indole-3-carboxamide | | [M+Na]+ | | | Organoheterocyclic compounds |  |
| 542 | 8.152 | 278.08014 | | N-[1-(4-Methoxy-6-oxopyran-2-yl)-2-methylpropyl]acetamide | | [M+K]+ | | | Organoheterocyclic compounds |  |
| 543 | 7.855 | 264.19135 | | N-Desmethylvenlafaxine | | [M+H]+ | | | Benzenoids |  |
| 544 | 3.048 | 152.07027 | | N-Methylanthranilic acid | | [M+H]+ | | | Benzenoids |  |
| 545 | 9.179 | 200.23358 | | N-Methyldodecylamine | | [M+H]+ | | | Organic nitrogen compounds |  |
| 546 | 11.11 | 430.32852 | | N-Oleoyl phenylalanine | | [M+H]+ | | | Organic acids and derivatives |  |
| 547 | 9.082 | 214.25418 | | N,N-Dimethyldodecylamine | | [M+H]+ | | | Organic nitrogen compounds |  |
| 548 | 9.127 | 230.2486 | | N,N-Dimethyldodecylamine N-oxide | | [M+H]+ | | | Organic nitrogen compounds |  |
| 549 | 7.62 | 250.17645 | | N,O-Didesmethylvenlafaxine | | [M+H]+ | | | Organic oxygen compounds |  |
| 550 | 3.69 | 353.08472 | | N(4)-Acetylsulfadimethoxine | | [M+H]+ | | | Benzenoids |  |
| 551 | 4.376 | 321.09372 | | N(4)-Acetylsulfamethazine | | [M+H]+ | | | Benzenoids |  |
| 552 | 1.095 | 282.11804 | | N6-Methyladenosine | | [M+H]+ | | | Nucleosides, nucleotides, and analogues |  |
| 553 | 6.526 | 745.19891 | | Naadp | | [M]+ | | | Nucleosides, nucleotides, and analogues |  |
| 554 | 7.422 | 275.1973 | | Nandrolone | | [M+H]+ | | | Lipids and lipid-like molecules |  |
| 555 | 9.24 | 128.06091 | | Naphthalene | | [M]+ | | | Benzenoids |  |
| 556 | 3.945 | 447.15936 | | Neosolaniol | | [M+Na]+ | | | Lipids and lipid-like molecules |  |
| 557 | 4.538 | 123.07719 | | Nicotinamide | | [M+H]+ | | | Organoheterocyclic compounds |  |
| 558 | 3.532 | 431.12433 | | Nodakenin | | [M+Na]+ | | | Phenylpropanoids and polyketides |  |
| 559 | 4.711 | 437.2337 | | Nonaethylene glycol | | [M+Na]+ | | | Organic oxygen compounds |  |
| 560 | 1.533 | 335.12958 | | Nor-Dentatin | | [M+Na]+ | | | Phenylpropanoids and polyketides |  |
| 561 | 4.327 | 293.09827 | | Norcimifugin | | [M+H]+ | | | Undefined |  |
| 562 | 0.726 | 152.07013 | | Norepinephrine | | [M-H2O+H]+ | | | Benzenoids |  |
| 563 | 2.299 | 118.06395 | | Norvaline | | [M+H]+ | | | Organic acids and derivatives |  |
| 564 | 12.168 | 601.42267 | | Nostoxanthin | | [M+H]+ | | | Lipids and lipid-like molecules |  |
| 565 | 11.345 | 955.51666 | | Notoginsenoside R1 | | [M+Na]+ | | | Lipids and lipid-like molecules |  |
| 566 | 11.214 | 259.20322 | | Octadeca-2,4,9-trien-6-yne-1,18-diol | | [M+H-H2O]+ | | | Undefined |  |
| 567 | 12.407 | 284.29306 | | Octadecanamide | | [M+H]+ | | | Organic acids and derivatives |  |
| 568 | 11.555 | 439.354 | | Oleanolic acid | | [M+H-H2O]+ | | | Lipids and lipid-like molecules |  |
| 569 | 3.512 | 447.16522 | | Orientanol E | | [M+Na]+ | | | Phenylpropanoids and polyketides |  |
| 570 | 4.451 | 405.17029 | | Osajin | | [M+H]+ | | | Phenylpropanoids and polyketides |  |
| 571 | 6.381 | 245.11472 | | Osthole | | [M+H]+ | | | Phenylpropanoids and polyketides |  |
| 572 | 7.296 | 485.23508 | | p-Mentha-1-ene-8-yl 6-O-(alpha-L-rhamnopyranosyl)-beta-D-glucopyranoside | | [M+Na]+ | | | Organic oxygen compounds |  |
| 573 | 4.589 | 294.13025 | | Paclobutrazol | | [M+H]+ | | | Benzenoids |  |
| 574 | 1.59 | 483.14536 | | Paeonolide | | [M+H]+ | | | Undefined |  |
| 575 | 0.667 | 360.14865 | | Palatinose | | [M+NH4]+ | | | Organic oxygen compounds |  |
| 576 | 11.758 | 256.26242 | | Palmitic amide | | [M+H]+ | | | Undefined |  |
| 577 | 12.523 | 205.19295 | | Patchouli alcohol | | [M+H]+ | | | Organic oxygen compounds |  |
| 578 | 10.698 | 454.33725 | | Peimine | | [M+Na]+ | | | Lipids and lipid-like molecules |  |
| 579 | 3.095 | 463.12167 | | Peonidin-3-O-beta-galactoside | | [M+H]+ | | | Phenylpropanoids and polyketides |  |
| 580 | 13.125 | 625.3999 | | Peonidin-3,5-O-di-beta-glucopyranoside | | [M]+ | | | Phenylpropanoids and polyketides |  |
| 581 | 7.138 | 315.19266 | | Pergolide | | [M+H]+ | | | Organoheterocyclic compounds |  |
| 582 | 5.394 | 151.11089 | | Perillene | | [M+H]+ | | | Undefined |  |
| 583 | 13.401 | 933.56067 | | Petunidin-3-O-(6''-O-(4'''-O-E-coum)-alpha-rhamnopyranosyl-beta-glucopyranosyl)-5-O-beta-glucopyranoside | | [M+H]+ | | | Phenylpropanoids and polyketides |  |
| 584 | 4.954 | 439.15839 | | Phenethyl 2-O-(beta-D-xylopyranosyl)-beta-D-glucopyranoside | | [M+Na]+ | | | Organic oxygen compounds |  |
| 585 | 6.101 | 165.08832 | | Phenethyl acetate | | [M+H]+ | | | Benzenoids |  |
| 586 | 3.914 | 469.1669 | | Phenethyl sophoroside | | [M+Na]+ | | | Organic oxygen compounds |  |
| 587 | 2.94 | 411.12256 | | Phenyl 6-O-[(2R,3R,4R)-3,4-dihydroxy-4-(hydroxymethyl)tetrahydro-2-furanyl]-beta-D-glucopyranoside | | [M+Na]+ | | | Organic oxygen compounds |  |
| 588 | 3.294 | 137.05765 | | Phenyl acetate | | [M+H-H2O]+ | | | Undefined |  |
| 589 | 2.959 | 121.06448 | | Phenylacetaldehyde | | [M-C6H10O5+H]+ | | | Benzenoids |  |
| 590 | 14.537 | 184.073 | | Phosphocholine | | [M]+ | | | Organic nitrogen compounds |  |
| 591 | 14.079 | 184.07074 | | Phosphorylcholine | | [M+H]+ | | | Organic nitrogen compounds |  |
| 592 | 12.482 | 149.02144 | | Phthalic anhydride | | [M+H]+ | | | Undefined |  |
| 593 | 10.737 | 318.29977 | | Phytosphingosine | | [M+H]+ | | | Organic nitrogen compounds |  |
| 594 | 2.009 | 209.12251 | | Pilocarpine | | [M+H]+ | | | Alkaloids and derivatives |  |
| 595 | 4.113 | 361.18787 | | Piperonyl butoxide | | [M+Na]+ | | | Organoheterocyclic compounds |  |
| 596 | 3.106 | 306.09479 | | Pirimiphos-methyl | | [M+H]+ | | | Organic acids and derivatives |  |
| 597 | 1.938 | 453.13376 | | Polygalatenoside A | | [M+Na]+ | | | Organic oxygen compounds |  |
| 598 | 3.498 | 443.15283 | | Pomiferin | | [M+Na]+ | | | Phenylpropanoids and polyketides |  |
| 599 | 4.401 | 469.16724 | | prim-O-b-D-Glucosylcimifugin | | [M+H]+ | | | Organoheterocyclic compounds |  |
| 600 | 5.661 | 493.1297 | | Primulin | | [M]+ | | | Phenylpropanoids and polyketides |  |
| 601 | 0.695 | 239.13692 | | Primuliten | | [M+Na]+ | | | Phenylpropanoids and polyketides |  |
| 602 | 12.21 | 315.23004 | | Progesterone | | [M+H]+ | | | Lipids and lipid-like molecules |  |
| 603 | 6.269 | 249.14532 | | Pterosin A | | [M+H]+ | | | Benzenoids |  |
| 604 | 6.281 | 235.12753 | | Pterosin G | | [M+H]+ | | | Benzenoids |  |
| 605 | 6.114 | 543.30322 | | Puberanidine | | [M+H]+ | | | Lipids and lipid-like molecules |  |
| 606 | 5.008 | 447.16226 | | Purpurein | | [M+Na]+ | | | Phenylpropanoids and polyketides |  |
| 607 | 1.58 | 332.13245 | | Pyridoxine + O-Hex | | [M+H]+ | | | Organoheterocyclic compounds |  |
| 608 | 10.283 | 322.14304 | | Pyriproxyfen | | [M+H]+ | | | Benzenoids |  |
| 609 | 7.702 | 325.19559 | | Quinidine HCl | | [M+H]+ | | | Undefined |  |
| 610 | 6.072 | 130.06516 | | Quinoline | | [M+H]+ | | | Organoheterocyclic compounds |  |
| 611 | 10.984 | 419.27737 | | Radiclonic acid | | [M+Na]+ | | | Lipids and lipid-like molecules |  |
| 612 | 7.876 | 401.21024 | | Reserpic acid | | [M+H]+ | | | Alkaloids and derivatives |  |
| 613 | 5.475 | 435.09116 | | Reynoutrin | | [M+H]+ | | | Phenylpropanoids and polyketides |  |
| 614 | 4.491 | 377.14633 | | Riboflavin | | [M+H]+ | | | Organoheterocyclic compounds |  |
| 615 | 7.42 | 180.17113 | | Rimantadine | | [M+H]+ | | | Organic nitrogen compounds |  |
| 616 | 5.475 | 743.28839 | | Ritonavir | | [M+Na]+ | | | Organic acids and derivatives |  |
| 617 | 5.145 | 741.22821 | | Robinin | | [M+H]+ | | | Phenylpropanoids and polyketides |  |
| 618 | 3.561 | 299.17029 | | Roquefortine A | | [M+H]+ | | | Alkaloids and derivatives |  |
| 619 | 2.836 | 163.03719 | | Rosmarinic acid | | [M+H-C9H10O5]+ | | | Phenylpropanoids and polyketides |  |
| 620 | 11.739 | 693.41663 | | Rumensin | | [M+H]+ | | | Organic oxygen compounds |  |
| 621 | 11.293 | 803.45703 | | Saikosaponin D | | [M+H]+ | | | Undefined |  |
| 622 | 14.351 | 773.48492 | | Salinomycin | | [M+Na]+ | | | Lipids and lipid-like molecules |  |
| 623 | 12.657 | 271.20419 | | Salvirecognine | | [M+H]+ | | | Undefined |  |
| 624 | 6.136 | 267.15646 | | Sambucinol | | [M+H]+ | | | Organoheterocyclic compounds |  |
| 625 | 3.046 | 425.1012 | | Samidin | | [M+K]+ | | | Phenylpropanoids and polyketides |  |
| 626 | 6.216 | 247.1292 | | Santonin | | [M+H]+ | | | Lipids and lipid-like molecules |  |
| 627 | 6.146 | 543.2182 | | Satratoxin F | | [M+H]+ | | | Lipids and lipid-like molecules |  |
| 628 | 4.643 | 551.2041 | | Satratoxin H | | [M+Na]+ | | | Lipids and lipid-like molecules |  |
| 629 | 4.037 | 439.15552 | | Sayaendoside | | [M+Na]+ | | | Organic oxygen compounds |  |
| 630 | 10.009 | 361.23282 | | Scopolamine-N-butyl | | [M+H]+ | | | Organic acids and derivatives |  |
| 631 | 4.605 | 193.05006 | | Scopoletin | | [M+H]+ | | | Phenylpropanoids and polyketides |  |
| 632 | 13.662 | 203.1758 | | Selina-4(14),7(11)-diene-9-ol;[1S-(1alpha,4abeta,8aalpha)]-Decahydro-8a-methyl-5-methylene-3-(1-methylethylidene)-1-naphthalenol | | [M+H-H2O]+ | | | Undefined |  |
| 633 | 3.953 | 273.13129 | | Sempervirine | | [M+H]+ | | | Organoheterocyclic compounds |  |
| 634 | 5.947 | 336.17865 | | Senecionine | | [M+H]+ | | | Phenylpropanoids and polyketides |  |
| 635 | 5.545 | 334.16168 | | Seneciphylline | | [M+H]+ | | | Alkaloids and derivatives |  |
| 636 | 7.539 | 193.12006 | | Senkyunolide A | | [M+H]+ | | | Undefined |  |
| 637 | 0.697 | 429.13345 | | Shanzhiside methyl ester | | [M+H]+ | | | Lipids and lipid-like molecules |  |
| 638 | 8.933 | 277.17773 | | Shogaol | | [M+H]+ | | | Benzenoids |  |
| 639 | 4.992 | 207.13622 | | Sinapoyl malate | | [M+H]+ | | | Phenylpropanoids and polyketides |  |
| 640 | 4.377 | 211.09599 | | Sinapyl alcohol | | [M+H]+ | | | Benzenoids |  |
| 641 | 13.705 | 868.50244 | | Solamargine | | [M+H]+ | | | Lipids and lipid-like molecules |  |
| 642 | 11.777 | 706.45813 | | Solanidine base + O-Hex-dHex | | [M+H]+ | | | Lipids and lipid-like molecules |  |
| 643 | 6.481 | 419.16644 | | Solvent green 3 | | [M+H]+ | | | Benzenoids |  |
| 644 | 6.859 | 249.19415 | | Sophoridine | | [M+H]+ | | | Undefined |  |
| 645 | 10.674 | 300.28989 | | Sphingosine | | [M+H]+ | | | Organic nitrogen compounds |  |
| 646 | 3.077 | 391.06461 | | Spiro[11-oxatricyclo[4.4.1.01,6]undeca-3,8-diene-10,3'-2,4-dioxatricyclo[7.3.1.05,13]trideca-1(12),5,7,9(13),10-pentaene]-2,5,7-triol | | [M+K]+ | | | Benzenoids |  |
| 647 | 13.148 | 411.39755 | | Squalene | | [M+H]+ | | | Lipids and lipid-like molecules |  |
| 648 | 11.387 | 921.48083 | | Stachybocin C | | [M+Na]+ | | | Organoheterocyclic compounds |  |
| 649 | 13.414 | 411.35739 | | STIGMASTA-4,22-DIEN-3-ONE | | [M+H]+ | | | Undefined |  |
| 650 | 12.248 | 413.37573 | | Stigmasterol | | [M+H]+ | | | Lipids and lipid-like molecules |  |
| 651 | 7.079 | 335.18042 | | Strychnine sulfate | | [M+H]+ | | | Undefined |  |
| 652 | 4.782 | 365.09796 | | Sucrose | | [M+Na]+ | | | Organic oxygen compounds |  |
| 653 | 4.066 | 183.06348 | | Syringaldehyde | | [M+H]+ | | | Benzenoids |  |
| 654 | 5.745 | 509.12854 | | Syringetin-3-o-glucoside | | [M+H]+ | | | Phenylpropanoids and polyketides |  |
| 655 | 3.891 | 395.13031 | | Syringin | | [M+Na]+ | | | Organic oxygen compounds |  |
| 656 | 4.445 | 667.27649 | | Syrosingopine | | [M+H]+ | | | Alkaloids and derivatives |  |
| 657 | 9.318 | 372.23141 | | Tamoxifen | | [M+H]+ | | | Phenylpropanoids and polyketides |  |
| 658 | 12.806 | 427.38956 | | Taraxasterol | | [M+H]+ | | | Lipids and lipid-like molecules |  |
| 659 | 5.901 | 415.22742 | | Tentoxin | | [M+H]+ | | | Organic acids and derivatives |  |
| 660 | 4.911 | 445.16464 | | Tetracycline | | [M+H]+ | | | Phenylpropanoids and polyketides |  |
| 661 | 11.337 | 363.30878 | | Tetraethylene glycol monododecyl ether | | [M+H]+ | | | Organic oxygen compounds |  |
| 662 | 7.337 | 233.15405 | | Threonylleucine | | [M+H]+ | | | Organic acids and derivatives |  |
| 663 | 4.415 | 329.15381 | | Tiapride | | [M+H]+ | | | Benzenoids |  |
| 664 | 5.367 | 409.14322 | | Tinnevellin glucoside | | [M+H]+ | | | Undefined |  |
| 665 | 14.505 | 445.367 | | Topsentisterol A2 | | [M+H]+ | | | Undefined |  |
| 666 | 4.962 | 343.13388 | | Trehalose | | [M+H]+ | | | Organic oxygen compounds |  |
| 667 | 9.472 | 301.21536 | | Tretinoin | | [M+H]+ | | | Lipids and lipid-like molecules |  |
| 668 | 10.506 | 443.20526 | | Trichoverrol A | | [M+Na]+ | | | Lipids and lipid-like molecules |  |
| 669 | 10.548 | 369.12396 | | Tricresylphosphate | | [M+H]+ | | | Organic acids and derivatives |  |
| 670 | 0.637 | 138.05351 | | Trigonelline | | [M+H]+ | | | Alkaloids and derivatives |  |
| 671 | 9.722 | 267.16779 | | Triisobutyl phosphate | | [M+H]+ | | | Organic acids and derivatives |  |
| 672 | 4.538 | 161.13229 | | Trinoranastreptene | | [M+H]+ | | | Undefined |  |
| 673 | 9.141 | 327.07666 | | Triphenyl phosphate | | [M+H]+ | | | Organic acids and derivatives |  |
| 674 | 7.71 | 313.17767 | | Triptophenolide | | [M+H]+ | | | Lipids and lipid-like molecules |  |
| 675 | 7.95 | 327.008 | | Tris(1-chloro-2-propyl)phosphate | | [M+H]+ | | | Organic acids and derivatives |  |
| 676 | 9.039 | 428.88837 | | Tris(1,3-dichloro-2-propyl)phosphate | | [M+H]+ | | | Organic acids and derivatives |  |
| 677 | 5.405 | 144.07941 | | Tryptophol | | [M+H-H2O]+ | | | Organoheterocyclic compounds |  |
| 678 | 2.733 | 138.0881 | | Tyramine | | [M+H]+ | | | Benzenoids |  |
| 679 | 6.185 | 369.18597 | | Uncarine A | | [M+H]+ | | | Organoheterocyclic compounds |  |
| 680 | 5.448 | 369.18839 | | Uncarine E | | [M+H]+ | | | Undefined |  |
| 681 | 6.911 | 369.18842 | | Uncarine F | | [M+H]+ | | | Organoheterocyclic compounds |  |
| 682 | 10.533 | 773.43848 | | Unguisin E | | [M+H]+ | | | Organic acids and derivatives |  |
| 683 | 6.781 | 163.11044 | | Valerophenone | | [M+H]+ | | | Organic oxygen compounds |  |
| 684 | 3.319 | 153.05319 | | Vanillin | | [M+H]+ | | | Benzenoids |  |
| 685 | 11.82 | 455.29138 | | Verapamil | | [M+H]+ | | | Benzenoids |  |
| 686 | 3.175 | 507.18896 | | Verrucarin J | | [M+Na]+ | | | Lipids and lipid-like molecules |  |
| 687 | 6.042 | 457.20193 | | Vindoline | | [M+H]+ | | | Alkaloids and derivatives |  |
| 688 | 7.866 | 351.21442 | | Vinpocetine | | [M+H]+ | | | Alkaloids and derivatives |  |
| 689 | 5.362 | 238.08549 | | Viridicatin | | [M+H]+ | | | Organoheterocyclic compounds |  |
| 690 | 14.464 | 431.38586 | | Vitamin E | | [M+H]+ | | | Lipids and lipid-like molecules |  |
| 691 | 7.18 | 235.16826 | | Walleminone | | [M-H2O+H]+ | | | Lipids and lipid-like molecules |  |
| 692 | 9.61 | 377.13516 | | Xanthohumol | | [M+Na]+ | | | Phenylpropanoids and polyketides |  |
| 693 | 2.976 | 531.16602 | | Xolegel | | [M+H]+ | | | Organoheterocyclic compounds |  |
| 694 | 3.43 | 289.07309 | | (-)-Epicatechin | | [M-H]- | | | Phenylpropanoids and polyketides |  |
| 695 | 5.509 | 459.12717 | | (-)-Podophyllotoxin | | [M+FA-H]- | | | Undefined |  |
| 696 | 4.098 | 289.0712 | | (+)-Epicatechin | | [M-H]- | | | Phenylpropanoids and polyketides |  |
| 697 | 8.307 | 329.23206 | | (15Z)-9,12,13-Trihydroxy-15-octadecenoic acid | | [M-H]- | | | Lipids and lipid-like molecules |  |
| 698 | 11.735 | 455.35397 | | (1S,2R,4As,6aS,6bR,10S,12aR)-10-hydroxy-1,2,6a,6b,9,9,12a-heptamethyl-2,3,4,5,6,6a,7,8,8a,10,11,12,13,14b-tetradecahydro-1H-picene-4a-carboxylic acid | | [M-H]- | | | Lipids and lipid-like molecules |  |
| 699 | 4.02 | 537.16205 | | (1S,4aS,7S,7aS)-1-[(2S,3R,4S,5S,6R)-6-[[(E)-3-(3,4-dihydroxyphenyl)prop-2-enoyl]oxymethyl]-3,4,5-trihydroxyoxan-2-yl]oxy-7-hydroxy-7-methyl-4a,5,6,7a-tetrahydro-1H-cyclopenta[c]pyran-4-carboxylic acid | | [M-H]- | | | Lipids and lipid-like molecules |  |
| 700 | 11.221 | 564.32996 | | (2-Hydroxy-3-octadeca-9,12-dienoyloxypropyl) 2-(trimethylazaniumyl)ethyl phosphate | | [M+HCOO]- | | | Lipids and lipid-like molecules |  |
| 701 | 10.61 | 562.31476 | | (2-Hydroxy-3-octadeca-9,12,15-trienoyloxypropyl) 2-(trimethylazaniumyl)ethyl phosphate | | [M+HCOO]- | | | Lipids and lipid-like molecules |  |
| 702 | 7.87 | 659.32281 | | (2E,6E,10E)-14-{[2-O-(beta-D-Glucopyranosyl)-beta-D-glucopyranosyl]oxy}-4-hydroxy-2,6,10,14-tetramethyl-2,6,10,15-hexadecatetraenoic acid | | [M-H]- | | | Lipids and lipid-like molecules |  |
| 703 | 0.694 | 303.04959 | | (2R,3R)-2-(2,6-Dihydroxyphenyl)-3,5,7-trihydroxy-2,3-dihydrochromen-4-one | | [M-H]- | | | Phenylpropanoids and polyketides |  |
| 704 | 5.866 | 459.18417 | | (2R,3S,4S,5R,6R)-2-[[(2S,3R,4R)-3,4-dihydroxy-4-(hydroxymethyl)oxolan-2-yl]oxymethyl]-6-[4-(4-hydroxyphenyl)butan-2-yloxy]oxane-3,4,5-triol | | [M-H]- | | | Lipids and lipid-like molecules |  |
| 705 | 3.652 | 371.0972 | | (2S,3S,4S,5R,6R)-6-(3-benzoyloxy-2-hydroxypropoxy)-3,4,5-trihydroxyoxane-2-carboxylic acid | | [M-H]- | | | Organic oxygen compounds |  |
| 706 | 5.375 | 287.05484 | | (2S,3S)-3,4',5,7-Tetrahydroxyflavanone | | [M-H]- | | | Phenylpropanoids and polyketides |  |
| 707 | 6.873 | 329.06674 | | (2Z)-4,6-Dihydroxy-2-[(4-hydroxy-3,5-dimethoxyphenyl)methylidene]-1-benzofuran-3-one | | [M-H]- | | | Phenylpropanoids and polyketides |  |
| 708 | 10.906 | 323.25763 | | (3S)-5-[(1R,2R,8aS)-2-hydroxy-2,5,5,8a-tetramethyl-3,4,4a,6,7,8-hexahydro-1H-naphthalen-1-yl]-3-methylpentanoic acid | | [M-H]- | | | Lipids and lipid-like molecules |  |
| 709 | 10.238 | 311.22107 | | (9Z,15Z)-12,13-Dihydroxyoctadeca-9,15-dienoate | | [M-H]- | | | Lipids and lipid-like molecules |  |
| 710 | 6.599 | 269.13791 | | (E)-2-Decylpent-2-enedioic acid | | [M-H]- | | | Lipids and lipid-like molecules |  |
| 711 | 3.161 | 367.09845 | | (E)-3-(3,4-Dihydroxyphenyl)acrylic acid (1R,2R,3R)-2,3,5-trihydroxy-5-(methoxycarbonyl)cyclohexyl ester | | [M-H]- | | | Organic oxygen compounds |  |
| 712 | 10.962 | 299.25705 | | (R)-2-Hydroxystearic acid | | [M-H]- | | | Undefined |  |
| 713 | 0.724 | 147.02921 | | (S)-2-Hydroxy-2-methylsuccinic acid | | [M-H]- | | | Lipids and lipid-like molecules |  |
| 714 | 5.726 | 519.11548 | | [2-(3,4-Dihydroxyphenyl)-5-hydroxy-7-methoxy-4-oxo-3-[(2S,3R,4R,5R,6S)-3,4,5-trihydroxy-6-methyloxan-2-yl]oxychromen-8-yl] acetate | | [M-H]- | | | Phenylpropanoids and polyketides |  |
| 715 | 11.322 | 561.32574 | | [2-Hydroxy-3-[3,4,5-trihydroxy-6-(hydroxymethyl)oxan-2-yl]oxypropyl] octadeca-9,12-dienoate | | [M+HCOO]- | | | Lipids and lipid-like molecules |  |
| 716 | 10.627 | 675.36011 | | [2-Hydroxy-3-[3,4,5-trihydroxy-6-[[3,4,5-trihydroxy-6-(hydroxymethyl)oxan-2-yl]oxymethyl]oxan-2-yl]oxypropyl] octadeca-9,12,15-trienoate | | [M-H]- | | | Lipids and lipid-like molecules |  |
| 717 | 11.242 | 476.27887 | | [3-[2-Aminoethoxy(hydroxy)phosphoryl]oxy-2-hydroxypropyl] octadeca-9,12-dienoate | | [M-H]- | | | Lipids and lipid-like molecules |  |
| 718 | 3.891 | 481.13834 | | [3-Hydroxy-4-[(2S,3R,4S,5S,6R)-3,4,5-trihydroxy-6-(hydroxymethyl)oxan-2-yl]oxyphenyl]methyl 4-hydroxy-3,5-dimethoxybenzoate | | [M-H]- | | | Phenylpropanoids and polyketides |  |
| 719 | 3.86 | 797.21063 | | [3,4-Dihydroxy-6-[5-hydroxy-2-(4-hydroxyphenyl)-4-oxo-7-[3,4,5-trihydroxy-6-(hydroxymethyl)oxan-2-yl]oxychromen-3-yl]oxy-5-[(2S,3R,4R,5R,6S)-3,4,5-trihydroxy-6-methyloxan-2-yl]oxyoxan-2-yl]methyl acetate | | [M-H]- | | | Phenylpropanoids and polyketides |  |
| 720 | 6.424 | 593.12793 | | [6-[2-(3,4-Dihydroxyphenyl)-8-hydroxy-4-oxochromen-7-yl]oxy-3,4,5-trihydroxyoxan-2-yl]methyl (E)-3-(4-hydroxyphenyl)prop-2-enoate | | [M-H]- | | | Phenylpropanoids and polyketides |  |
| 721 | 6.582 | 679.35699 | | 1-O-[(2alpha,3beta,5xi,9xi,18xi)-2,3,19,24-Tetrahydroxy-24,28-dioxoolean-12-en-28-yl]-beta-D-glucopyranose | | [M-H]- | | | Lipids and lipid-like molecules |  |
| 722 | 11.209 | 809.43365 | | 1-O-[(3beta,5xi,9xi,18xi)-3-(beta-D-Glucopyranuronosyloxy)-23-hydroxy-28-oxoolean-12-en-28-yl]-beta-D-glucopyranose | | [M-H]- | | | Lipids and lipid-like molecules |  |
| 723 | 10.395 | 793.44232 | | 1-O-[(3beta,5xi,9xi,18xi)-3-(beta-D-Glucopyranuronosyloxy)-28-oxoolean-12-en-28-yl]-beta-D-glucopyranose | | [M-H]- | | | Lipids and lipid-like molecules |  |
| 724 | 11.536 | 540.32892 | | 1-Palmitoylphosphatidylcholine | | [M+HCOO]- | | | Lipids and lipid-like molecules |  |
| 725 | 7.887 | 333.20343 | | 1,10a-Dihydroxy-4,4,7,11b-tetramethyl-2,3,4,4a,5,6,6a,7,10a,11,11a,11b-dodecahydrophenanthro[3,2-b]furan-9(1H)-one | | [M-H]- | | | Organoheterocyclic compounds |  |
| 726 | 3.176 | 337.0918 | | 1,3,4-Trihydroxy-5-[[3-(4-hydroxyphenyl)-1-oxo-2-propenyl]oxy]-,(1r,3r,4s,5r)-cyclohexanecarboxylic acid | | [M-H]- | | | Organic oxygen compounds |  |
| 727 | 8.711 | 593.26147 | | 1,7-Bis(4-hydroxyphenyl)-3-heptanyl 6-O-[(2S,3R,4R)-3,4-dihydroxy-4-(hydroxymethyl)tetrahydro-2-furanyl]-beta-D-glucopyranoside | | [M-H]- | | | Phenylpropanoids and polyketides |  |
| 728 | 3.289 | 455.09976 | | 11-(2-Bromo-3,6-dimethoxyphenyl)-3,3-dimethyl-2,3,4-trihydro-5H,10H,11H-benzo[b]benzo[2,1-f]1,4-diazepin-1-one | | [M-H]- | | | Organoheterocyclic compounds |  |
| 729 | 12.109 | 309.2059 | | 12-Hydroperoxyoctadeca-9,13,15-trienoic acid | | [M-H]- | | | Lipids and lipid-like molecules |  |
| 730 | 9.415 | 313.23755 | | 12,13-Dihydroxyoctadec-9-enoic acid | | [M-H]- | | | Lipids and lipid-like molecules |  |
| 731 | 8.052 | 317.21136 | | 12S-Hepe | | [M-H]- | | | Lipids and lipid-like molecules |  |
| 732 | 10.81 | 295.22586 | | 13-HODE | | [M-H]- | | | Undefined |  |
| 733 | 9.158 | 293.21176 | | 13-HOTrE | | [M-H]- | | | Undefined |  |
| 734 | 12.674 | 311.22354 | | 13-HPODE | | [M-H]- | | | Undefined |  |
| 735 | 7.676 | 223.13477 | | 13-Oxotrideca-9,11-dienoic acid | | [M-H]- | | | Lipids and lipid-like molecules |  |
| 736 | 10.095 | 293.21426 | | 14-(3-Ethyloxiran-2-YL)tetradeca-9,12-dienoic acid | | [M-H]- | | | Lipids and lipid-like molecules |  |
| 737 | 9.149 | 463.19943 | | 16-Glucuronide-estriol | | [M-H]- | | | Lipids and lipid-like molecules |  |
| 738 | 12.011 | 633.37482 | | 16-Hydroxy-3-(beta-D-xylopyranosyloxy)-, (3beta,5xi,9xi,16alpha)-olean-12-ene-23,28-dioic acid | | [M-H]- | | | Lipids and lipid-like molecules |  |
| 739 | 8.068 | 307.19107 | | 16-Hydroxy-9-oxooctadeca-10,12,14-trienoic acid | | [M-H]- | | | Lipids and lipid-like molecules |  |
| 740 | 7.819 | 505.3382 | | 19-Hydroxy-18-[3,4,5-trihydroxy-6-(hydroxymethyl)oxan-2-yl]oxyicosanoic acid | | [M-H]- | | | Lipids and lipid-like molecules |  |
| 741 | 2.852 | 132.0443 | | 1H-Indazol-6-amine | | [M-H]- | | | Organoheterocyclic compounds |  |
| 742 | 5.115 | 595.12671 | | 2-(3,4-Dihydroxyphenyl)-5,7-dihydroxy-3-[(2S,3R,4S,5S,6R)-3,4,5-trihydroxy-6-[[(2S,3R,4S,5R)-3,4,5-trihydroxyoxan-2-yl]oxymethyl]oxan-2-yl]oxychromen-4-one | | [M-H]- | | | Phenylpropanoids and polyketides |  |
| 743 | 9.529 | 249.14964 | | 2-[(1S,2S,4aR,8aS)-1-Hydroxy-4a-methyl-8-methylenedecahydro-2-naphthalenyl]acrylic acid | | [M-H]- | | | Lipids and lipid-like molecules |  |
| 744 | 11.681 | 478.29111 | | 2-Azaniumylethyl (2-hydroxy-3-octadec-9-enoyloxypropyl) phosphate | | [M-H]- | | | Lipids and lipid-like molecules |  |
| 745 | 11.551 | 452.27557 | | 2-Azaniumylethyl (3-hexadecanoyloxy-2-hydroxypropyl) phosphate | | [M-H]- | | | Lipids and lipid-like molecules |  |
| 746 | 10.206 | 309.17343 | | 2-Dodecoxyethyl hydrogen sulfate | | [M-H]- | | | Organic acids and derivatives |  |
| 747 | 10.721 | 373.23788 | | 2-Hydroxy-4-methoxy-3,5-bis(3-methylbut-2-enyl)-6-pentylbenzoic acid | | [M-H]- | | | Benzenoids |  |
| 748 | 3.086 | 131.07109 | | 2-Hydroxy-4-methylvaleric acid | | [M-H]- | | | Lipids and lipid-like molecules |  |
| 749 | 9.474 | 341.21143 | | 2-Hydroxy-6-[(8Z,11Z)-pentadeca-8,11,14-trienyl]benzoic acid | | [M-H]- | | | Benzenoids |  |
| 750 | 13.018 | 355.32037 | | 2-Hydroxydocosanoic acid | | [M-H]- | | | Lipids and lipid-like molecules |  |
| 751 | 2.542 | 151.04144 | | 2-Hydroxyphenylacetic acid | | [M-H]- | | | Benzenoids |  |
| 752 | 2.37 | 175.06117 | | 2-Isopropylmalic acid | | [M-H]- | | | Lipids and lipid-like molecules |  |
| 753 | 10.72 | 297.2421 | | 2-Methyl-4-oxo-heptadecanoic acid | | [M-H]- | | | Lipids and lipid-like molecules |  |
| 754 | 1.516 | 277.09122 | | 2-Methylidene-4-[(2R,3R,4S,5S,6R)-3,4,5-trihydroxy-6-(hydroxymethyl)oxan-2-yl]oxybutanoic acid | | [M-H]- | | | Lipids and lipid-like molecules |  |
| 755 | 4.792 | 207.01237 | | 2-Naphthalenesulfonic acid | | [M-H]- | | | Benzenoids |  |
| 756 | 0.748 | 145.01434 | | 2-Oxoglutaric acid | | [M-H]- | | | Organic acids and derivatives |  |
| 757 | 0.655 | 245.04362 | | 2,2',4,4'-Tetrahydroxybenzophenone | | [M-H]- | | | Benzenoids |  |
| 758 | 2.305 | 355.06464 | | 2,3,4-Trihydroxy-5-[3-(4-hydroxyphenyl)prop-2-enoyloxy]hexanedioic acid | | [M-H]- | | | Organic oxygen compounds |  |
| 759 | 5.373 | 195.06747 | | 2,4-Dihydroxy-6-propylbenzoic acid | | [M-H]- | | | Benzenoids |  |
| 760 | 5.946 | 229.04984 | | 2,4,6-Trihydroxybenzophenone | | [M-H]- | | | Benzenoids |  |
| 761 | 11.015 | 491.30182 | | 2'-Deoxyinosine triphosphate | | [M-H]- | | | Nucleosides, nucleotides, and analogues |  |
| 762 | 3.77 | 357.11661 | | 3-(2-Glucosyloxy-4-methoxyphenyl)propanoic acid | | [M-H]- | | | Organic oxygen compounds |  |
| 763 | 4.47 | 193.04976 | | 3-(4-Hydroxy-3-methoxyphenyl)-2-propenoic acid | | [M-H]- | | | Phenylpropanoids and polyketides |  |
| 764 | 4.611 | 195.06575 | | 3-(4-Hydroxy-3-methoxyphenyl)propionic acid | | [M-H]- | | | Phenylpropanoids and polyketides |  |
| 765 | 3.274 | 163.04077 | | 3-(4-Hydroxyphenyl)-2-propenoic acid | | [M-H]- | | | Phenylpropanoids and polyketides |  |
| 766 | 11.302 | 559.30975 | | 3-(Hexopyranosyloxy)-2-hydroxypropyl ester, (9Z,12Z,15Z)-9,12,15-octadecatrienoic acid | | [M+FA-H]- | | | Lipids and lipid-like molecules |  |
| 767 | 5.073 | 915.25201 | | 3-[(2S,3R,4S,5S,6R)-4,5-Dihydroxy-3-[(2S,3R,4R,5R,6S)-3,4,5-trihydroxy-6-methyloxan-2-yl]oxy-6-[[(2S,3R,4R,5R,6S)-3,4,5-trihydroxy-6-methyloxan-2-yl]oxymethyl]oxan-2-yl]oxy-5-hydroxy-2-(4-hydroxyphenyl)-6-methoxy-7-[(2S,3R,4R,5R,6S)-3,4,5-trihydroxy-6-methyloxan-2-yl]oxychromen-4-one | | [M-H]- | | | Phenylpropanoids and polyketides |  |
| 768 | 4.543 | 421.16418 | | 3-[(7S,8S)-5-Hydroxy-2,2,7,8-tetramethyl-6-oxo-7,8-dihydropyrano[3,2-g]chromen-10-yl]-3-phenylpropanoic acid | | [M-H]- | | | Organoheterocyclic compounds |  |
| 769 | 4.593 | 343.10147 | | 3-[2-Hydroxy-3-[(2S,3R,4S,5S,6R)-3,4,5-trihydroxy-6-(hydroxymethyl)oxan-2-yl]oxyphenyl]propanoic acid | | [M-H]- | | | Organic oxygen compounds |  |
| 770 | 3.501 | 353.08926 | | 3-[3-(3,4-Dihydroxyphenyl)prop-2-enoyloxy]-1,4,5-trihydroxycyclohexane-1-carboxylic acid | | [M-H]- | | | Organic oxygen compounds |  |
| 771 | 4.85 | 755.20233 | | 3-[6-[[(2R,3R,4R,5S,6S)-3,5-Dihydroxy-6-methyl-4-[(2S,3R,4R,5R,6S)-3,4,5-trihydroxy-6-methyloxan-2-yl]oxyoxan-2-yl]oxymethyl]-3,4,5-trihydroxyoxan-2-yl]oxy-2-(3,4-dihydroxyphenyl)-5,7-dihydroxychromen-4-one | | [M-H]- | | | Phenylpropanoids and polyketides |  |
| 772 | 2.917 | 195.03133 | | 3-Formyl-2,4-dihydroxy-6-methylbenzoic acid | | [M-H]- | | | Benzenoids |  |
| 773 | 11.198 | 319.19046 | | 3-HYDROXY-4-(SUCCIN-2-YL)-CARYOLANE delta-LACTONE | | [M-H]- | | | Undefined |  |
| 774 | 10.927 | 893.48944 | | 3-Hydroxy-6-[(9-hydroxy-4,4,6a,6b,8a,11,11,14b-octamethyl-1,2,3,4a,5,6,7,8,9,10,12,14a-dodecahydropicen-3-yl)oxy]-4-(3,4,5-trihydroxy-6-methyloxan-2-yl)oxy-5-(3,4,5-trihydroxyoxan-2-yl)oxyoxane-2-carboxylic acid | | [M-H]- | | | Lipids and lipid-like molecules |  |
| 775 | 6.526 | 167.0351 | | 3-Hydroxymandelic acid | | [M-H]- | | | Benzenoids |  |
| 776 | 4.463 | 217.10834 | | 3-Hydroxysebacic acid | | [M-H]- | | | Organic acids and derivatives |  |
| 777 | 1.869 | 409.16986 | | 3-Isomangostin | | [M-H]- | | | Undefined |  |
| 778 | 3.256 | 137.02422 | | 3,4-Dihydroxybenzaldehyde | | [M-H]- | | | Organic oxygen compounds |  |
| 779 | 1.898 | 153.01892 | | 3,4-Dihydroxybenzoic acid | | [M-H]- | | | Benzenoids |  |
| 780 | 4.946 | 167.03444 | | 3,4-Dihydroxyphenylacetic acid | | [M-H]- | | | Benzenoids |  |
| 781 | 4.167 | 449.10788 | | 3,5-Dihydroxy-2-(4-hydroxyphenyl)-7-[3,4,5-trihydroxy-6-(hydroxymethyl)oxan-2-yl]oxy-2,3-dihydrochromen-4-one | | [M-H]- | | | Phenylpropanoids and polyketides |  |
| 782 | 5.967 | 497.16577 | | 3,5-Dihydroxy-4-[3,4,5-trihydroxy-6-[[4-(2-hydroxypropan-2-yl)cyclohexene-1-carbonyl]oxymethyl]oxan-2-yl]oxybenzoic acid | | [M-H]- | | | Lipids and lipid-like molecules |  |
| 783 | 9.71 | 297.15067 | | 4-(Decan-4-yl)benzenesulfonic acid | | [M-H]- | | | Benzenoids |  |
| 784 | 2.701 | 327.10629 | | 4-Acetyl-3-hydroxy-5-methylphenyl beta-D-glucopyranoside | | [M-H]- | | | Organic oxygen compounds |  |
| 785 | 3.076 | 353.08789 | | 4-Caffeoylquinic acid;4-O-Caffeoylquinic acid | | [M-H]- | | | Organic oxygen compounds |  |
| 786 | 1.13 | 121.02799 | | 4-Hydroxybenzaldehyde | | [M-H]- | | | Organic oxygen compounds |  |
| 787 | 1.59 | 148.03934 | | 4-Hydroxymandelonitrile | | [M-H]- | | | Benzenoids |  |
| 788 | 2.883 | 144.04478 | | 4-Hydroxyquinoline | | [M-H]- | | | Organoheterocyclic compounds |  |
| 789 | 5.423 | 165.05772 | | 4-Methoxyphenylacetic acid | | [M-H]- | | | Benzenoids |  |
| 790 | 14.552 | 167.03622 | | 4-Methoxysalicylic acid | | [M-H]- | | | Benzenoids |  |
| 791 | 14.114 | 138.0191 | | 4-Nitrophenol | | [M-H]- | | | Benzenoids |  |
| 792 | 1.74 | 182.04506 | | 4-Pyridoxic acid | | [M-H]- | | | Organoheterocyclic compounds |  |
| 793 | 4.109 | 119.04932 | | 4-Vinylphenol | | [M-H]- | | | Benzenoids |  |
| 794 | 7.36 | 291.12244 | | 4,5,6,7,8,9-Hexahydro-11,13-dihydroxy-4-methyl-, (S)-2H-3-benzoxacyclododecin-2,10(1H)-dione | | [M-H]- | | | Phenylpropanoids and polyketides |  |
| 795 | 10.307 | 311.16876 | | 5-(4-Sulfophenyl)undecane | | [M-H]- | | | Benzenoids |  |
| 796 | 10.542 | 383.21896 | | 5-[(Z)-12-(3,5-Dihydroxyphenyl)dodec-8-enyl]benzene-1,3-diol | | [M-H]- | | | Benzenoids |  |
| 797 | 8.958 | 331.19028 | | 5-[2-(Furan-3-yl)ethyl]-8-hydroxy-5,6,8a-trimethyl-3,4,4a,6,7,8-hexahydronaphthalene-1-carboxylic acid | | [M-H]- | | | Lipids and lipid-like molecules |  |
| 798 | 4.535 | 609.17657 | | 5-Hydroxy-2-(3-hydroxy-4-methoxyphenyl)-7-[(2S,3R,4S,5S,6R)-3,4,5-trihydroxy-6-[[(2R,3R,4R,5R,6S)-3,4,5-trihydroxy-6-methyloxan-2-yl]oxymethyl]oxan-2-yl]oxy-2,3-dihydrochromen-4-one | | [M-H]- | | | Phenylpropanoids and polyketides |  |
| 799 | 6.458 | 301.07089 | | 5,7-Dihydroxy-2-(4-hydroxy-3-methoxyphenyl)-2,3-dihydrochromen-4-one | | [M-H]- | | | Phenylpropanoids and polyketides |  |
| 800 | 5.604 | 623.15619 | | 5,7-Dihydroxy-2-(4-hydroxy-3-methoxyphenyl)-3-[3,4,5-trihydroxy-6-[[(2R,3R,4R,5R,6S)-3,4,5-trihydroxy-6-methyloxan-2-yl]oxymethyl]oxan-2-yl]oxychromen-4-one | | [M-H]- | | | Phenylpropanoids and polyketides |  |
| 801 | 5.648 | 507.11365 | | 5,7-Dihydroxy-2-(4-hydroxy-3,5-dimethoxyphenyl)-3-[(2S,3R,4S,5R,6R)-3,4,5-trihydroxy-6-(hydroxymethyl)oxan-2-yl]oxychromen-4-one | | [M-H]- | | | Phenylpropanoids and polyketides |  |
| 802 | 4.794 | 433.11411 | | 5,7-Dihydroxy-2-(4-hydroxyphenyl)-6-[3,4,5-trihydroxy-6-(hydroxymethyl)oxan-2-yl]-2,3-dihydrochromen-4-one | | [M-H]- | | | Phenylpropanoids and polyketides |  |
| 803 | 4.097 | 177.0197 | | 5,7-Dihydroxychromone | | [M-H]- | | | Undefined |  |
| 804 | 6.887 | 345.06174 | | 5,7,3',4'-Tetrahydroxy-6,8-dimethoxyflavone | | [M-H]- | | | Phenylpropanoids and polyketides |  |
| 805 | 6.427 | 461.10791 | | 5,8-Dihydroxy-2-(4-hydroxyphenyl)-7-methoxy-3-[(2S,3R,4R,5R,6S)-3,4,5-trihydroxy-6-methyloxan-2-yl]oxychromen-4-one | | [M-H]- | | | Phenylpropanoids and polyketides |  |
| 806 | 2.293 | 296.07925 | | 5'-Deoxy-5'-methylthioadenosine | | [M-H]- | | | Nucleosides, nucleotides, and analogues |  |
| 807 | 5.171 | 389.21841 | | 6-Hydroxy-3-[(1E)-3-hydroxy-1-buten-1-yl]-2,4,4-trimethylcyclohexyl beta-D-glucopyranoside | | [M-H]- | | | Lipids and lipid-like molecules |  |
| 808 | 0.693 | 285.03964 | | 6-Hydroxyapigenin | | [M-H]- | | | Phenylpropanoids and polyketides |  |
| 809 | 4.794 | 259.06216 | | 6-Methoxy-7-(3-methylbut-2-enoxy)chromen-2-one | | [M-H]- | | | Phenylpropanoids and polyketides |  |
| 810 | 11.211 | 825.4325 | | 6-O-beta-D-Glucopyranosyl-1-O-[(3beta,5xi,9xi)-3,19,24-trihydroxy-24,28-dioxours-12-en-28-yl]-beta-D-glucopyranose | | [M-H]- | | | Lipids and lipid-like molecules |  |
| 811 | 7.009 | 349.19885 | | 6,10a-Dihydroxy-4-(hydroxymethyl)-4,7,11b-trimethyl-1,2,3,4a,5,6,6a,7,11,11a-decahydronaphtho[2,1-f][1]benzofuran-9-one | | [M-H]- | | | Organoheterocyclic compounds |  |
| 812 | 5.515 | 579.13251 | | 6''-O-L-Arabinopyranosylastragalin | | [M-H]- | | | Phenylpropanoids and polyketides |  |
| 813 | 5.174 | 769.22186 | | 7-O-Methylquercetin-3-O-galactoside-6''-rhamnoside | | [M-H]- | | | Phenylpropanoids and polyketides |  |
| 814 | 8.24 | 291.19714 | | 8-(2-Oxo-5-pent-2-enylcyclopent-3-en-1-yl)octanoic acid | | [M-H]- | | | Lipids and lipid-like molecules |  |
| 815 | 2.578 | 379.15845 | | 8-Desoxygartanin | | [M-H]- | | | Undefined |  |
| 816 | 10.203 | 293.21082 | | 8-hydroxyoctadeca-9,12,15-trienoic acid | | [M-H]- | | | Lipids and lipid-like molecules |  |
| 817 | 0.798 | 339.12885 | | 8-Prenylnaringenin | | [M-H]- | | | Phenylpropanoids and polyketides |  |
| 818 | 4.441 | 337.14822 | | 9-((3,7-Dimethylocta-2,6-dien-1-yl)-oxy)-7H-furo[3,2-g]chromen-7-one | | [M-H]- | | | Lipids and lipid-like molecules |  |
| 819 | 11.796 | 253.21703 | | 9-Hexadecenoic acid | | [M-H]- | | | Lipids and lipid-like molecules |  |
| 820 | 9.834 | 293.21393 | | 9-HOTrE | | [M-H]1- | | | Lipids and lipid-like molecules |  |
| 821 | 8.918 | 311.22131 | | 9-Hydroperoxyoctadeca-10,12-dienoic acid | | [M-H]- | | | Lipids and lipid-like molecules |  |
| 822 | 10.664 | 295.22836 | | 9-Hydroxy-10E,12Z-octadecadienoic acid | | [M-H]- | | | Lipids and lipid-like molecules |  |
| 823 | 9.597 | 309.20551 | | 9-Oxo-11-(3-pentyloxiran-2-YL)undec-10-enoic acid | | [M-H]- | | | Lipids and lipid-like molecules |  |
| 824 | 8.654 | 329.23239 | | 9,12,13-Trihydroxyoctadec-10-enoic acid | | [M-H]- | | | Lipids and lipid-like molecules |  |
| 825 | 7.82 | 327.21625 | | 9,12,13-Trihydroxyoctadeca-10,15-dienoic acid | | [M-H]- | | | Lipids and lipid-like molecules |  |
| 826 | 11.916 | 301.21503 | | Abietic acid | | [M-H]- | | | Lipids and lipid-like molecules |  |
| 827 | 1.323 | 150.05579 | | Acetaminophen | | [M-H]- | | | Benzenoids |  |
| 828 | 2.893 | 172.09708 | | Acetylleucine | | [M-H]- | | | Organic acids and derivatives |  |
| 829 | 1.328 | 134.04677 | | Adenine | | [M-H]- | | | Organoheterocyclic compounds |  |
| 830 | 2.636 | 266.08759 | | Adenosine | | [M-H]- | | | Nucleosides, nucleotides, and analogues |  |
| 831 | 4.359 | 465.14447 | | Agnuside | | [M-H]- | | | Undefined |  |
| 832 | 11.66 | 749.4458 | | alpha-Hederin | | [M-H]- | | | Undefined |  |
| 833 | 7.938 | 715.35083 | | Amatain | | [M-H]- | | | Alkaloids and derivatives |  |
| 834 | 9.25 | 537.30591 | | Amentoflavone | | [M-H]- | | | Phenylpropanoids and polyketides |  |
| 835 | 4.041 | 327.10645 | | Androsin | | [M-H]- | | | Organic oxygen compounds |  |
| 836 | 6.853 | 269.04453 | | Apigenin | | [M-H]- | | | Phenylpropanoids and polyketides |  |
| 837 | 6.102 | 739.2049 | | Apigenin 7-O-(2G-rhamnosyl)gentiobioside | | [M-H]- | | | Undefined |  |
| 838 | 6.082 | 577.15442 | | Apigenin-7-O-neohesperidoside | | [M-H]- | | | Undefined |  |
| 839 | 11.892 | 303.23419 | | Arachidonic acid | | [M-H]- | | | Lipids and lipid-like molecules |  |
| 840 | 0.835 | 271.08139 | | Arbutin | | [M-H]- | | | Undefined |  |
| 841 | 4.227 | 517.15582 | | Arillatose B | | [M-H]- | | | Phenylpropanoids and polyketides |  |
| 842 | 9.432 | 487.33783 | | Asiatic acid | | [M-H]- | | | Undefined |  |
| 843 | 5.23 | 449.10693 | | Astilbin | | [M-H]- | | | Undefined |  |
| 844 | 7.268 | 247.13429 | | Atractylenolide III | | [M-H]- | | | Undefined |  |
| 845 | 6.303 | 491.11954 | | Aurantio-obtusin beta-D-glucoside | | [M-H]- | | | Benzenoids |  |
| 846 | 11.424 | 487.34357 | | Bayogenin | | [M-H]- | | | Undefined |  |
| 847 | 1.986 | 329.0871 | | Benzoic acid + 1O, 1MeO, O-Hex | | [M-H]- | | | Phenylpropanoids and polyketides |  |
| 848 | 0.717 | 315.06769 | | Benzoic acid + 2O, O-Hex | | [M-H]- | | | Organic oxygen compounds |  |
| 849 | 4.015 | 213.96384 | | Benzothiazole-2-sulfonic acid | | [M-H]- | | | Organoheterocyclic compounds |  |
| 850 | 3.026 | 151.03955 | | Benzoylformic acid | | [M+FA-H]- | | | Benzenoids |  |
| 851 | 13.052 | 455.3508 | | Betulinic acid | | [M-H]- | | | Lipids and lipid-like molecules |  |
| 852 | 5.516 | 407.19226 | | Bidwillon A | | [M-H]- | | | Phenylpropanoids and polyketides |  |
| 853 | 10.391 | 469.32974 | | Biosone | | [M-C6H10O5-H]- | | | Lipids and lipid-like molecules |  |
| 854 | 3.955 | 243.0887 | | Biotin | | [M-H]- | | | Organoheterocyclic compounds |  |
| 855 | 5.928 | 219.17763 | | Butylated hydroxytoluene | | [M-H]- | | | Benzenoids |  |
| 856 | 7.521 | 193.08644 | | Butylparaben | | [M-H]- | | | Benzenoids |  |
| 857 | 3.508 | 179.03519 | | Caffeic acid | | [M-H]- | | | Phenylpropanoids and polyketides |  |
| 858 | 3.1 | 341.08444 | | Caffeic acid hexoside | | [M-H]- | | | Phenylpropanoids and polyketides |  |
| 859 | 5.617 | 725.19757 | | Camelliaside B | | [M-H]- | | | Undefined |  |
| 860 | 10.319 | 339.19452 | | Canrenone | | [M-H]- | | | Lipids and lipid-like molecules |  |
| 861 | 5.108 | 298.07193 | | Chlordiazepoxide | | [M-H]- | | | Organoheterocyclic compounds |  |
| 862 | 2.532 | 353.08594 | | Chlorogenic acid | | [M-H]- | | | Organic oxygen compounds |  |
| 863 | 6.1 | 253.05054 | | Chrysin | | [M-H]- | | | Phenylpropanoids and polyketides |  |
| 864 | 3.331 | 289.07254 | | Cianidanol | | [M-H]- | | | Phenylpropanoids and polyketides |  |
| 865 | 8.132 | 357.14343 | | Cilastatin | | [M-H]- | | | Organic acids and derivatives |  |
| 866 | 5.618 | 193.04958 | | Cinnamic acid | | [M-H]- | | | Phenylpropanoids and polyketides |  |
| 867 | 0.744 | 173.00829 | | cis-Aconitic acid | | [M-H]- | | | Organic acids and derivatives |  |
| 868 | 3.71 | 353.08841 | | Clove-3 | | [M-H]- | | | Organic oxygen compounds |  |
| 869 | 6.744 | 327.2168 | | Corchorifatty acid F | | [M-H]- | | | Lipids and lipid-like molecules |  |
| 870 | 10.877 | 299.25952 | | Coronaric acid | | [M-H]1- | | | Lipids and lipid-like molecules |  |
| 871 | 12.461 | 471.34332 | | Corosolic acid | | [M-H]- | | | Undefined |  |
| 872 | 3.977 | 325.09067 | | Coumaric acid 4-O-glucoside | | [M-H]- | | | Organic oxygen compounds |  |
| 873 | 3.145 | 325.09271 | | Coumaric acid O-glucoside | | [M-H]- | | | Organic oxygen compounds |  |
| 874 | 3.136 | 515.13824 | | Coumarin + 1O + 1MeO, O-Hex-Hex | | [M-H]- | | | Phenylpropanoids and polyketides |  |
| 875 | 3.723 | 337.09125 | | Coumaroyl quinic acid | | [M-H]- | | | Organic oxygen compounds |  |
| 876 | 11.38 | 471.34464 | | Crategolic acid | | [M-H]- | | | Lipids and lipid-like molecules |  |
| 877 | 11.274 | 887.48853 | | Cy 3-coumSamb-5-Glc | | [M-H]- | | | Phenylpropanoids and polyketides |  |
| 878 | 4.809 | 285.03964 | | Cyanidin | | [M-2H]- | | | Phenylpropanoids and polyketides |  |
| 879 | 3.72 | 755.19971 | | Cyanidin 3-(2G-glucosylrutinoside) | | [M-2H]- | | | Undefined |  |
| 880 | 6.305 | 431.09839 | | Cyanidin-3-o-rhamnoside | | [M-2H]- | | | Phenylpropanoids and polyketides |  |
| 881 | 2.208 | 134.0582 | | Cyclic AMP | | [M-H]- | | | Nucleosides, nucleotides, and analogues |  |
| 882 | 10.131 | 373.15738 | | Cyproterone | | [M-H]- | | | Lipids and lipid-like molecules |  |
| 883 | 0.812 | 117.01988 | | D-Erythronolactone | | [M-H]- | | | Organoheterocyclic compounds |  |
| 884 | 0.688 | 193.07085 | | D-Pinitol | | [M-H]- | | | Organic oxygen compounds |  |
| 885 | 12.801 | 637.36511 | | Decahydrogambogic acid | | [M-H]- | | | Organoheterocyclic compounds |  |
| 886 | 11.217 | 299.20123 | | Dehydroabietic acid | | [M-H]- | | | Lipids and lipid-like molecules |  |
| 887 | 12.42 | 483.34683 | | Dehydrotumulosic acid | | [M-H]- | | | Undefined |  |
| 888 | 4.641 | 595.12781 | | Delphinidin 3-O-beta-D-sambubioside | | [M-2H]- | | | Phenylpropanoids and polyketides |  |
| 889 | 10.733 | 413.3017 | | delta(4)-Dafachronic acid | | [M-H]- | | | Lipids and lipid-like molecules |  |
| 890 | 5.23 | 299.01831 | | Demethylwedelolactone | | [M-H]- | | | Undefined |  |
| 891 | 6.172 | 503.17413 | | Deoxynivalenol 3-glucoside | | [M+HCOO]- | | | Lipids and lipid-like molecules |  |
| 892 | 6.594 | 403.13867 | | Desoxyrhaponticin | | [M-H]- | | | Undefined |  |
| 893 | 2.817 | 391.12024 | | Digoxigenin | | [M-H]- | | | Lipids and lipid-like molecules |  |
| 894 | 4.148 | 327.10648 | | Dihydrocoumaroyl Hexoside | | [M-H]- | | | Organic oxygen compounds |  |
| 895 | 3.795 | 611.19275 | | Dihydrohesperetin-7-O-neohesperidoside | | [M-H]- | | | Undefined |  |
| 896 | 5.375 | 435.12833 | | Dihydrostilbene base + 3O, 1carboxy, O-Hex | | [M-H]- | | | Phenylpropanoids and polyketides |  |
| 897 | 8.245 | 357.26239 | | Dinoprost | | [M-H]1- | | | Lipids and lipid-like molecules |  |
| 898 | 5.716 | 607.17096 | | Diosmetin-7-O-rutinoside | | [M-H]- | | | Phenylpropanoids and polyketides |  |
| 899 | 14.541 | 721.50214 | | Dipalmitoylphosphatidylglycerol | | [M-H]- | | | Lipids and lipid-like molecules |  |
| 900 | 14.02 | 339.32413 | | Docosanoic acid | | [M-H]- | | | Lipids and lipid-like molecules |  |
| 901 | 10.57 | 325.18448 | | Dodecylbenzenesulfonic acid | | [M-H]- | | | Benzenoids |  |
| 902 | 5.652 | 263.12875 | | Dormin | | [M-H]- | | | Lipids and lipid-like molecules |  |
| 903 | 6.884 | 555.29059 | | Dronedarone | | [M-H]- | | | Organic oxygen compounds |  |
| 904 | 11.9 | 471.34808 | | Echinocystic acid | | [M-H]- | | | Undefined |  |
| 905 | 4.841 | 787.26593 | | Eleutheroside E | | [M+FA-H]- | | | Undefined |  |
| 906 | 9.547 | 293.17709 | | Embelin | | [M-H]- | | | Organic oxygen compounds |  |
| 907 | 5.64 | 433.1153 | | Engeletin | | [M-H]- | | | Undefined |  |
| 908 | 9.123 | 469.32968 | | Enoxolone | | [M-H]- | | | Lipids and lipid-like molecules |  |
| 909 | 8.425 | 297.11731 | | Enterolactone | | [M-H]- | | | Undefined |  |
| 910 | 2.32 | 305.06662 | | Epigallocatechin | | [M-H]- | | | Phenylpropanoids and polyketides |  |
| 911 | 4.952 | 423.13196 | | Eprosartan | | [M-H]- | | | Benzenoids |  |
| 912 | 5.06 | 595.16949 | | Eriocitrin | | [M-H]- | | | Phenylpropanoids and polyketides |  |
| 913 | 5.94 | 287.05606 | | Eriodictyol | | [M-H]- | | | Phenylpropanoids and polyketides |  |
| 914 | 13.738 | 734.47626 | | Erythromycin | | [M-H]- | | | Organic oxygen compounds |  |
| 915 | 3.848 | 177.0192 | | Esculetin | | [M-H]- | | | Phenylpropanoids and polyketides |  |
| 916 | 3.73 | 339.06937 | | Esculin | | [M-H]- | | | Phenylpropanoids and polyketides |  |
| 917 | 10.509 | 271.17395 | | Estradiol | | [M-H]- | | | Lipids and lipid-like molecules |  |
| 918 | 9.635 | 269.15317 | | Estrona | | [M-H]- | | | Lipids and lipid-like molecules |  |
| 919 | 7.784 | 329.23526 | | FA 18:1;3O | | [M-H]- | | | Lipids and lipid-like molecules |  |
| 920 | 11.708 | 295.22568 | | FA 18:2;O | | [M-H]- | | | Lipids and lipid-like molecules |  |
| 921 | 7.395 | 325.20132 | | FA 18:3;3O | | [M-H]- | | | Lipids and lipid-like molecules |  |
| 922 | 12.395 | 275.20337 | | FA 18:4 | | [M-H]- | | | Lipids and lipid-like molecules |  |
| 923 | 10.727 | 291.1958 | | FA 18:4;O | | [M-H]- | | | Lipids and lipid-like molecules |  |
| 924 | 13.141 | 297.27917 | | FA 19:0 | | [M-H]- | | | Lipids and lipid-like molecules |  |
| 925 | 11.965 | 327.28793 | | FA 20:0;2O | | [M-H]- | | | Lipids and lipid-like molecules |  |
| 926 | 12.93 | 309.27676 | | FA 20:1 | | [M-H]- | | | Lipids and lipid-like molecules |  |
| 927 | 12.8 | 307.26242 | | FA 20:2 | | [M-H]- | | | Lipids and lipid-like molecules |  |
| 928 | 12.18 | 305.2475 | | FA 20:3 | | [M-H]- | | | Lipids and lipid-like molecules |  |
| 929 | 11.993 | 369.30203 | | FA 22:1;2O | | [M-H]- | | | Lipids and lipid-like molecules |  |
| 930 | 13.263 | 369.33496 | | FA 23:0;2O | | [M-H]- | | | Lipids and lipid-like molecules |  |
| 931 | 13.511 | 383.3512 | | FA 24:0;2O | | [M-H]- | | | Lipids and lipid-like molecules |  |
| 932 | 13.224 | 411.38086 | | FA 26:0;2O | | [M-H]- | | | Lipids and lipid-like molecules |  |
| 933 | 14.33 | 441.37097 | | FA 30:5 | | [M-H]- | | | Lipids and lipid-like molecules |  |
| 934 | 3.719 | 193.05145 | | Ferulic acid | | [M-H]- | | | Phenylpropanoids and polyketides |  |
| 935 | 3.178 | 281.06418 | | Feruloyl Lactate | | [M-H]- | | | Phenylpropanoids and polyketides |  |
| 936 | 0.645 | 313.11508 | | Flavokawain A | | [M-H]- | | | Undefined |  |
| 937 | 4.965 | 771.19574 | | Flavonol base + 4O, O-Hex-Hex, O-dHex | | [M-H]- | | | Phenylpropanoids and polyketides |  |
| 938 | 7.595 | 487.21207 | | Flemiphilippinin A | | [M-H]- | | | Undefined |  |
| 939 | 7.522 | 295.06949 | | Flunixin | | [M-H]- | | | Benzenoids |  |
| 940 | 5.627 | 267.0675 | | Formononetin | | [M-H]- | | | Phenylpropanoids and polyketides |  |
| 941 | 0.576 | 209.0302 | | Galactaric acid | | [M-H]- | | | Organic oxygen compounds |  |
| 942 | 4.328 | 755.18793 | | gamma-Aminobutyrate | | [M-H]- | | | Organic acids and derivatives |  |
| 943 | 10.464 | 277.21597 | | gamma-Linolenic acid | | [M-H]- | | | Lipids and lipid-like molecules |  |
| 944 | 8.133 | 293.17734 | | Gingerol | | [M-H]- | | | Benzenoids |  |
| 945 | 8.871 | 319.22681 | | Ginkgolic acid (C13:0) | | [M-H]- | | | Undefined |  |
| 946 | 12.563 | 829.4826 | | Ginsenoside F2 | | [M+FA-H]- | | | Undefined |  |
| 947 | 12.36 | 769.46161 | | Ginsenoside F3 | | [M-H]- | | | Undefined |  |
| 948 | 13.788 | 945.53986 | | Ginsenoside Re | | [M-H]- | | | Undefined |  |
| 949 | 14.097 | 765.46393 | | Ginsenoside Rg5 | | [M-H]- | | | Undefined |  |
| 950 | 12.772 | 683.42377 | | Ginsenoside Rh1 | | [M+FA-H]- | | | Undefined |  |
| 951 | 10.091 | 667.44476 | | Ginsenoside Rh2 (S-FORM) | | [M+FA-H]- | | | Undefined |  |
| 952 | 12.26 | 845.47333 | | Ginsenoside-Rg1 | | [M+FA-H]- | | | Undefined |  |
| 953 | 10.556 | 467.31354 | | Glabrolide | | [M-H]- | | | Undefined |  |
| 954 | 0.686 | 335.09326 | | Glabrone | | [M-H]- | | | Phenylpropanoids and polyketides |  |
| 955 | 0.615 | 195.0517 | | Gluconic acid | | [M-H]- | | | Organic acids and derivatives |  |
| 956 | 3.111 | 293.12433 | | Glutamylphenylalanine | | [M-H]- | | | Organic acids and derivatives |  |
| 957 | 6.613 | 517.19189 | | Gossypol | | [M-H]- | | | Lipids and lipid-like molecules |  |
| 958 | 5.473 | 433.07828 | | Guaijaverin | | [M-H]- | | | Phenylpropanoids and polyketides |  |
| 959 | 0.833 | 282.08289 | | Guanosine | | [M-H]- | | | Nucleosides, nucleotides, and analogues |  |
| 960 | 13.709 | 325.30939 | | Heneicosanoic acid | | [M-H]- | | | Lipids and lipid-like molecules |  |
| 961 | 13.062 | 269.24603 | | Heptadecanoic acid | | [M-H]- | | | Lipids and lipid-like molecules |  |
| 962 | 5.291 | 609.17816 | | Hesperetin-7-O-rutinoside | | [M-H]- | | | Undefined |  |
| 963 | 9.875 | 285.20798 | | Hexadecanedioic acid | | [M-H]- | | | Lipids and lipid-like molecules |  |
| 964 | 6.36 | 395.18927 | | Hexose + C10H19O2 | | [M+HCOO]- | | | Organic oxygen compounds |  |
| 965 | 6.027 | 415.19534 | | Hexose + C13H19O | | [M+HCOO]- | | | Lipids and lipid-like molecules |  |
| 966 | 7.537 | 537.08026 | | Hinokiflavone | | [M-H]- | | | Phenylpropanoids and polyketides |  |
| 967 | 6.066 | 181.05205 | | Homovanillic acid | | [M-H]- | | | Benzenoids |  |
| 968 | 4.748 | 461.16684 | | Hydrangeifolin I | | [M+FA-H]- | | | Organic oxygen compounds |  |
| 969 | 0.741 | 191.01981 | | Hydron;2-hydroxypropane-1,2,3-tricarboxylate | | [M-H]- | | | Organic acids and derivatives |  |
| 970 | 0.72 | 129.01935 | | Hydron;2-methylidenebutanedioate | | [M-H]- | | | Lipids and lipid-like molecules |  |
| 971 | 1.467 | 121.02879 | | Hydron;benzoate | | [M-H]- | | | Benzenoids |  |
| 972 | 10.733 | 293.17868 | | Hydron;tetradecyl sulfate | | [M-H]- | | | Organic acids and derivatives |  |
| 973 | 6.905 | 299.05453 | | Hydroxygenkwanin | | [M-H]- | | | Undefined |  |
| 974 | 10.373 | 391.28409 | | Hyodeoxycholic acid | | [M-H]- | | | Lipids and lipid-like molecules |  |
| 975 | 4.151 | 503.13904 | | Hypericin | | [2M+Hac-H]- | | | Benzenoids |  |
| 976 | 4.801 | 463.08472 | | Hyperoside | | [M-H]- | | | Phenylpropanoids and polyketides |  |
| 977 | 4.77 | 174.05487 | | Indole-3-acetic acid | | [M-H]- | | | Organoheterocyclic compounds |  |
| 978 | 0.64 | 179.05595 | | Inositol | | [M-H]- | | | Organic oxygen compounds |  |
| 979 | 7.356 | 427.22836 | | Irbesartan | | [M-H]- | | | Benzenoids |  |
| 980 | 3.647 | 435.09299 | | Irisxanthone | | [M-H]- | | | Organoheterocyclic compounds |  |
| 981 | 7.316 | 367.11646 | | Isoanhydroicaritin | | [M-H]- | | | Undefined |  |
| 982 | 2.794 | 323.1358 | | Isobavachalcone | | [M-H]- | | | Phenylpropanoids and polyketides |  |
| 983 | 6.465 | 565.11346 | | Isoginkgetin | | [M-H]- | | | Undefined |  |
| 984 | 5.04 | 463.08719 | | Isoquercitrin | | [M-H]- | | | Phenylpropanoids and polyketides |  |
| 985 | 5.078 | 607.26013 | | Isoreserpin | | [M-H]- | | | Alkaloids and derivatives |  |
| 986 | 5.731 | 315.04834 | | Isorhamnetin | | [M-H]- | | | Phenylpropanoids and polyketides |  |
| 987 | 5.76 | 477.09988 | | Isorhamnetin 3-galactoside | | [M-H]- | | | Phenylpropanoids and polyketides |  |
| 988 | 5.76 | 639.15839 | | Isorhamnetin 3,4'-diglucoside | | [M-H]- | | | Phenylpropanoids and polyketides |  |
| 989 | 4.348 | 639.15363 | | isorhamnetin-3-glucoside-4'-glucoside (Isorhamnetin 3,4'-diglucoside) | | [M-H]- | | | Undefined |  |
| 990 | 5.089 | 623.16241 | | Isorhamnetin-3-O-galactoside-6''-rhamnoside | | [M-H]- | | | Undefined |  |
| 991 | 5.69 | 593.1842 | | isosakuranetin-7-O-rutinoside | | [M-H]- | | | Undefined |  |
| 992 | 7.25 | 209.11787 | | Jasmonic acid | | [M-H]- | | | Lipids and lipid-like molecules |  |
| 993 | 6.771 | 285.03986 | | Kaempferol | | [M-H]- | | | Phenylpropanoids and polyketides |  |
| 994 | 3.728 | 739.20337 | | Kaempferol 3-O-(2,6-di-O-alpha-L-rhamnopyranosyl)-beta-D-galactopyranoside | | [M-H]- | | | Phenylpropanoids and polyketides |  |
| 995 | 5.853 | 417.08072 | | Kaempferol 3-O-beta-D-xyloside | | [M-H]- | | | Phenylpropanoids and polyketides |  |
| 996 | 3.53 | 609.14777 | | Kaempferol 3-O-sophoroside | | [M-H]- | | | Undefined |  |
| 997 | 8.132 | 609.34033 | | Kaempferol 3,7-diglucoside | | [M-H]- | | | Phenylpropanoids and polyketides |  |
| 998 | 5.566 | 593.15295 | | Kaempferol 7-neohesperidoside | | [M-H]- | | | Phenylpropanoids and polyketides |  |
| 999 | 5.263 | 593.14557 | | Kaempferol-3-Glucoside-3''-Rhamnoside | | [M-H]- | | | Phenylpropanoids and polyketides |  |
| 1000 | 5.701 | 447.09186 | | Kaempferol-3-O-glucoside | | [M-H]- | | | Undefined |  |
| 1001 | 2.925 | 593.12927 | | Kaempferol-3-O-glucoside-6''-p-coumaroyl | | [M-H]- | | | Undefined |  |
| 1002 | 5.692 | 593.15381 | | Kaempferol-3-O-rutinoside | | [M-H]- | | | Undefined |  |
| 1003 | 8.757 | 529.29785 | | Kukoamine A | | [M-H]- | | | Undefined |  |
| 1004 | 9.934 | 529.30194 | | Kukoamine B | | [M-H]- | | | Undefined |  |
| 1005 | 3.059 | 188.033 | | Kynurenic acid | | [M-H]- | | | Organoheterocyclic compounds |  |
| 1006 | 3.454 | 165.05618 | | L-(-)-3-Phenyllactic acid | | [M-H]- | | | Phenylpropanoids and polyketides |  |
| 1007 | 0.63 | 133.01343 | | L-Malic acid | | [M-H]- | | | Organic acids and derivatives |  |
| 1008 | 0.668 | 341.10745 | | Lactulose | | [M-H]- | | | Organic oxygen compounds |  |
| 1009 | 8.265 | 265.14294 | | Lauryl sulfate | | [M-H]- | | | Organic acids and derivatives |  |
| 1010 | 5.181 | 461.11349 | | Leucanthoside | | [M-H]- | | | Phenylpropanoids and polyketides |  |
| 1011 | 0.815 | 130.08678 | | Leucine | | [M-H]- | | | Organic acids and derivatives |  |
| 1012 | 7.043 | 335.22305 | | Leukotriene B4 | | [M-H]1- | | | Lipids and lipid-like molecules |  |
| 1013 | 5.403 | 387.14255 | | Levocetirizine | | [M-H]- | | | Benzenoids |  |
| 1014 | 2.761 | 337.1506 | | Licochalcone A | | [M-H]- | | | Undefined |  |
| 1015 | 10.499 | 821.3985 | | Licoricesaponin H2 | | [M-H]- | | | Undefined |  |
| 1016 | 5.093 | 723.21118 | | Ligustroflavone | | [M-H]- | | | Undefined |  |
| 1017 | 13.027 | 279.23111 | | Linoleic acid | | [M-H]- | | | Lipids and lipid-like molecules |  |
| 1018 | 12.825 | 277.21719 | | Linolenic acid | | [M-H]- | | | Lipids and lipid-like molecules |  |
| 1019 | 6.391 | 417.11752 | | Liquiritin | | [M-H]- | | | Undefined |  |
| 1020 | 7.266 | 315.12531 | | Loureirin B | | [M-H]- | | | Undefined |  |
| 1021 | 6.286 | 257.08173 | | Lunularic acid | | [M-H]- | | | Phenylpropanoids and polyketides |  |
| 1022 | 6.422 | 285.03983 | | Luteolin | | [M-H]- | | | Phenylpropanoids and polyketides |  |
| 1023 | 5.015 | 593.15369 | | Luteolin 7-O-rutinoside | | [M-H]- | | | Phenylpropanoids and polyketides |  |
| 1024 | 13.098 | 503.33548 | | Madecassic acid | | [M-H]- | | | Undefined |  |
| 1025 | 0.69 | 287.0538 | | Maesopsin | | [M-H]- | | | Phenylpropanoids and polyketides |  |
| 1026 | 6.042 | 491.11658 | | Malvidin 3-O-glucoside | | [M-2H]- | | | Phenylpropanoids and polyketides |  |
| 1027 | 3.732 | 421.07925 | | Mangiferin | | [M-H]- | | | Organoheterocyclic compounds |  |
| 1028 | 0.615 | 259.0123 | | Mannose 6-phosphate | | [M-H]- | | | Organic oxygen compounds |  |
| 1029 | 10.285 | 501.3219 | | Medicagenic acid | | [M-H]- | | | Lipids and lipid-like molecules |  |
| 1030 | 0.721 | 161.04482 | | Meglutol | | [M-H]- | | | Lipids and lipid-like molecules |  |
| 1031 | 0.673 | 503.15787 | | Melezitose | | [M-H]- | | | Organic oxygen compounds |  |
| 1032 | 5.365 | 493.09991 | | Methoxy-myricetin-3-O-hexoside | | [M-H]- | | | Phenylpropanoids and polyketides |  |
| 1033 | 3.992 | 367.10294 | | Methyl chlorogenate | | [M-H]- | | | Organic oxygen compounds |  |
| 1034 | 12.678 | 183.00851 | | Methyl gallate | | [M-H]- | | | Benzenoids |  |
| 1035 | 10.027 | 315.2518 | | Methyl hexadecanoate | | [M-H]- | | | Undefined |  |
| 1036 | 5.158 | 341.10541 | | Methylophiopogonanone A | | [M-H]- | | | Undefined |  |
| 1037 | 8.098 | 443.22479 | | Mitragynine | | [M+FA-H]- | | | Undefined |  |
| 1038 | 5.933 | 301.03421 | | Morin | | [M-H]- | | | Phenylpropanoids and polyketides |  |
| 1039 | 4.853 | 479.0827 | | Myricetin-3-Galactoside | | [M-H]- | | | Phenylpropanoids and polyketides |  |
| 1040 | 5.909 | 479.07971 | | Myricetin-3-O-galactoside | | [M-H]- | | | Phenylpropanoids and polyketides |  |
| 1041 | 3.277 | 449.07556 | | Myricetin-3-O-xyloside | | [M-H]- | | | Undefined |  |
| 1042 | 11.468 | 227.20007 | | Myristic acid | | [M-H]- | | | Lipids and lipid-like molecules |  |
| 1043 | 3.051 | 206.08208 | | N-Acetyl-L-phenylalanine | | [M-H]- | | | Organic acids and derivatives |  |
| 1044 | 1.007 | 176.03896 | | N-Formylmethionine | | [M-H]- | | | Organic acids and derivatives |  |
| 1045 | 3.248 | 250.07082 | | N-Phenylacetylaspartic acid | | [M-H]- | | | Organic acids and derivatives |  |
| 1046 | 5.382 | 823.21057 | | n-Propionyl coenzyme A | | [M]- | | | Lipids and lipid-like molecules |  |
| 1047 | 4.43 | 623.15839 | | Narcissin | | [M-H]- | | | Phenylpropanoids and polyketides |  |
| 1048 | 6.46 | 271.06076 | | Naringenin | | [M-H]- | | | Phenylpropanoids and polyketides |  |
| 1049 | 4.45 | 433.11584 | | Naringenin-7-O-glucoside | | [M-H]- | | | Undefined |  |
| 1050 | 5.054 | 579.16937 | | Naringenin-7-O-rutinoside | | [M-H]- | | | Undefined |  |
| 1051 | 4.812 | 579.16943 | | Naringin | | [M-H]- | | | Undefined |  |
| 1052 | 5.955 | 581.18927 | | Naringin dihydrochalcone | | [M-H]- | | | Undefined |  |
| 1053 | 3.277 | 353.08603 | | Neochlorogenic acid | | [M-H]- | | | Organic oxygen compounds |  |
| 1054 | 5.505 | 609.17407 | | Neohesperidin | | [M-H]- | | | Undefined |  |
| 1055 | 7.003 | 215.06993 | | Nepodin | | [M-H]- | | | Undefined |  |
| 1056 | 14.087 | 365.33841 | | Nervonic acid | | [M-H]- | | | Lipids and lipid-like molecules |  |
| 1057 | 6.56 | 269.04449 | | Nordazepam | | [M-H]- | | | Organoheterocyclic compounds |  |
| 1058 | 6.9 | 191.03468 | | Noreugenin | | [M-H]- | | | Undefined |  |
| 1059 | 6.071 | 563.13678 | | NP-000062(6) | | [M-H]- | | | Undefined |  |
| 1060 | 10.72 | 313.23676 | | Octadecanedioic acid | | [M-H]- | | | Lipids and lipid-like molecules |  |
| 1061 | 10.33 | 453.33475 | | Oleanonic acid | | [M-H]- | | | Undefined |  |
| 1062 | 11.385 | 281.24664 | | Oleic acid | | [M-H]- | | | Lipids and lipid-like molecules |  |
| 1063 | 5.423 | 123.04506 | | Orcinol | | [M-H]- | | | Benzenoids |  |
| 1064 | 4.769 | 447.09235 | | Orientin | | [M-H]- | | | Phenylpropanoids and polyketides |  |
| 1065 | 3.075 | 167.03494 | | Orsellinic acid | | [M-H]- | | | Benzenoids |  |
| 1066 | 3.192 | 345.08578 | | Oryzalin | | [M-H]- | | | Benzenoids |  |
| 1067 | 14.115 | 166.05067 | | Oxfenicine | | [M-H]- | | | Organic acids and derivatives |  |
| 1068 | 3.128 | 147.04436 | | p-Coumaraldehyde | | [M-H]- | | | Phenylpropanoids and polyketides |  |
| 1069 | 3.195 | 171.0125 | | P-Toluenesulfonic acid | | [M-H]- | | | Benzenoids |  |
| 1070 | 11.643 | 255.23106 | | Palmitic acid | | [M-H]- | | | Lipids and lipid-like molecules |  |
| 1071 | 11.425 | 253.21712 | | Palmitoleic acid | | [M-H]- | | | Lipids and lipid-like molecules |  |
| 1072 | 2.453 | 218.10271 | | Pantothenic acid | | [M-H]- | | | Organic acids and derivatives |  |
| 1073 | 13.891 | 716.52972 | | PE 34:1 | | [M-H]- | | | Undefined |  |
| 1074 | 13.674 | 714.49957 | | PE 34:2 | | [M-H]- | | | Undefined |  |
| 1075 | 12.951 | 742.54279 | | PE 36:2 | | [M-H]- | | | Undefined |  |
| 1076 | 13.651 | 740.52277 | | PE 36:3 | | [M-H]- | | | Undefined |  |
| 1077 | 11.292 | 738.50616 | | PE 36:4 | | [M-H]- | | | Undefined |  |
| 1078 | 13.196 | 381.37405 | | Pentacosanoic acid | | [M-H]- | | | Lipids and lipid-like molecules |  |
| 1079 | 11.932 | 241.21692 | | Pentadecanoic acid | | [M-H]- | | | Lipids and lipid-like molecules |  |
| 1080 | 6.972 | 493.22946 | | Pentose-Hexose + C10H17 | | [M+HCOO]- | | | Lipids and lipid-like molecules |  |
| 1081 | 2.919 | 477.10648 | | Petunidin 3-galactoside | | [M-2H]- | | | Phenylpropanoids and polyketides |  |
| 1082 | 13.861 | 693.47473 | | PG 30:0 | | [M-H]- | | | Undefined |  |
| 1083 | 14.217 | 719.49231 | | PG 32:1 | | [M-H]- | | | Undefined |  |
| 1084 | 11.764 | 747.51599 | | PG 34:1 | | [M-H]- | | | Undefined |  |
| 1085 | 14.097 | 745.50421 | | PG 34:2 | | [M-H]- | | | Undefined |  |
| 1086 | 13.861 | 743.48792 | | PG 34:3 | | [M-H]- | | | Undefined |  |
| 1087 | 12.57 | 773.53339 | | PG 36:2 | | [M-H]- | | | Undefined |  |
| 1088 | 14.186 | 771.51971 | | PG 36:3 | | [M-H]- | | | Undefined |  |
| 1089 | 2.915 | 93.03496 | | Phenol | | [M-C6H10O5-H]- | | | Benzenoids |  |
| 1090 | 3.104 | 164.07059 | | Phenylalanine | | [M-H]- | | | Organic acids and derivatives |  |
| 1091 | 4.108 | 163.03957 | | Phenylpyruvic acid | | [M-H]- | | | Benzenoids |  |
| 1092 | 6.525 | 273.07913 | | Phloretin | | [M-H]- | | | Phenylpropanoids and polyketides |  |
| 1093 | 5.639 | 435.12885 | | Phlorizin | | [M-H]- | | | Phenylpropanoids and polyketides |  |
| 1094 | 11.669 | 506.32184 | | Phosphatidylcholine lyso 18:1 | | [M-CH3]- | | | Lipids and lipid-like molecules |  |
| 1095 | 11.222 | 504.30807 | | Phosphatidylcholine lyso 18:2 | | [M-CH3]- | | | Lipids and lipid-like molecules |  |
| 1096 | 10.611 | 502.293 | | Phosphatidylcholine lyso 18:3 | | [M-CH3]- | | | Lipids and lipid-like molecules |  |
| 1097 | 14.529 | 835.53656 | | PI 34:1 | | [M-H]- | | | Undefined |  |
| 1098 | 14.014 | 833.52209 | | PI 34:2 | | [M-H]- | | | Undefined |  |
| 1099 | 12.8 | 847.51831 | | PI 35:2 | | [M-H]- | | | Undefined |  |
| 1100 | 12.382 | 861.55505 | | PI 36:2 | | [M-H]- | | | Undefined |  |
| 1101 | 12.328 | 855.49017 | | PI 36:5 | | [M-H]- | | | Undefined |  |
| 1102 | 10.407 | 487.30554 | | Polygalic acid | | [M-H]- | | | Undefined |  |
| 1103 | 11.425 | 497.32458 | | Poricoic acid A | | [M-H]- | | | Undefined |  |
| 1104 | 8.43 | 423.23987 | | Pravastatin | | [M-H]- | | | Organic acids and derivatives |  |
| 1105 | 4.696 | 577.13086 | | Procyanidin B1 | | [M-H]- | | | Phenylpropanoids and polyketides |  |
| 1106 | 3.595 | 577.13135 | | Procyanidin B2 | | [M-H]- | | | Phenylpropanoids and polyketides |  |
| 1107 | 3.885 | 865.19696 | | Procyanidin C1 | | [M-H]- | | | Phenylpropanoids and polyketides |  |
| 1108 | 0.79 | 335.09003 | | Psoralidin | | [M-H]- | | | Phenylpropanoids and polyketides |  |
| 1109 | 6.182 | 415.10068 | | Puerarin | | [M-H]- | | | Phenylpropanoids and polyketides |  |
| 1110 | 1.341 | 166.05119 | | Pyridoxal | | [M-H]- | | | Organoheterocyclic compounds |  |
| 1111 | 1.323 | 168.06662 | | Pyridoxine | | [M-H]- | | | Organoheterocyclic compounds |  |
| 1112 | 6.036 | 301.03296 | | Quercetin | | [M-H]- | | | Phenylpropanoids and polyketides |  |
| 1113 | 5.66 | 505.09604 | | Quercetin 3-O-(6''-acetyl-glucoside) | | [M-H]- | | | Phenylpropanoids and polyketides |  |
| 1114 | 5.974 | 755.18433 | | Quercetin 3-O-[2''-O-(6'''-O-p-coumaroyl)-b-D-glucopyranosyl]-a-L-rhamnopyranoside | | [M-H]- | | | Undefined |  |
| 1115 | 5.221 | 549.08685 | | Quercetin 3-O-malonylglucoside | | [M-H]- | | | Phenylpropanoids and polyketides |  |
| 1116 | 5.29 | 625.13947 | | Quercetin 3-O-sophoroside | | [M-H]- | | | Phenylpropanoids and polyketides |  |
| 1117 | 5.187 | 771.19818 | | Quercetin 3-rutinoside 7-galactoside | | [M-H]- | | | Phenylpropanoids and polyketides |  |
| 1118 | 5.219 | 505.0993 | | Quercetin-3-O-glucosyl-6''-acetate | | [M-H]- | | | Undefined |  |
| 1119 | 3.807 | 625.13641 | | Quercetin-3,4'-O-di-beta-glucoside | | [M-H]- | | | Undefined |  |
| 1120 | 5.214 | 445.07654 | | Quercitrin | | [M-C6H10O4-H]- | | | Phenylpropanoids and polyketides |  |
| 1121 | 9.244 | 485.32709 | | Quillaic acid | | [M-H]- | | | Lipids and lipid-like molecules |  |
| 1122 | 0.621 | 405.10037 | | QUINATE | | [2M-2H+Na]- | | | Undefined |  |
| 1123 | 0.619 | 191.05756 | | Quinic acid | | [M-H]- | | | Organic oxygen compounds |  |
| 1124 | 5.159 | 151.04057 | | Resorcinol monoacetate | | [M-H]- | | | Benzenoids |  |
| 1125 | 4.19 | 639.15576 | | Rhamnetin 3-sophoroside | | [M-H]- | | | Phenylpropanoids and polyketides |  |
| 1126 | 5.651 | 577.1543 | | Rhoifolin | | [M-H]- | | | Undefined |  |
| 1127 | 13.942 | 439.35303 | | Roburic acid | | [M-H]- | | | Undefined |  |
| 1128 | 7.959 | 253.05151 | | Rubiadin | | [M-H]- | | | Benzenoids |  |
| 1129 | 6.266 | 493.20993 | | Rubranoside A | | [M-H]- | | | Phenylpropanoids and polyketides |  |
| 1130 | 4.948 | 609.14569 | | Rutin | | [M-H]- | | | Phenylpropanoids and polyketides |  |
| 1131 | 1.914 | 383.11139 | | S-Adenosyl-L-homocysteine | | [M-H]- | | | Nucleosides, nucleotides, and analogues |  |
| 1132 | 8.132 | 925.50616 | | Saikosaponin C | | [M-H]- | | | Undefined |  |
| 1133 | 3.655 | 137.02344 | | Salicylic acid | | [M-H]- | | | Benzenoids |  |
| 1134 | 2.636 | 299.07529 | | Salicylic acid glucoside | | [M-H]- | | | Organic oxygen compounds |  |
| 1135 | 2.962 | 299.11545 | | Salidroside | | [M-H]- | | | Organic oxygen compounds |  |
| 1136 | 4.359 | 593.14923 | | Scolymoside | | [M-H]- | | | Phenylpropanoids and polyketides |  |
| 1137 | 5.668 | 361.16394 | | Secoisolariciresinol | | [M-H]- | | | Lignans, neolignans and related compounds |  |
| 1138 | 3.527 | 223.05988 | | Sinapic acid | | [M-H]- | | | Phenylpropanoids and polyketides |  |
| 1139 | 11.085 | 328.15417 | | Sinomenine | | [M-H]- | | | Undefined |  |
| 1140 | 12.321 | 911.50092 | | Soyasapogenol B base + O-HexA-Pen-dHex | | [M-H]- | | | Lipids and lipid-like molecules |  |
| 1141 | 13.23 | 957.49939 | | Soyasaponin Ba | | [M-H]- | | | Lipids and lipid-like molecules |  |
| 1142 | 14.266 | 941.51263 | | Soyasaponin Bb | | [M-H]- | | | Undefined |  |
| 1143 | 11.052 | 201.11211 | | Spermine | | [M-H]- | | | Organic nitrogen compounds |  |
| 1144 | 11.946 | 283.26181 | | Stearic acid | | [M-H]- | | | Lipids and lipid-like molecules |  |
| 1145 | 7.233 | 641.3125 | | Steviolbioside | | [M-H]- | | | Undefined |  |
| 1146 | 0.73 | 117.02001 | | Succinic acid | | [M-H]- | | | Organic acids and derivatives |  |
| 1147 | 6.149 | 507.11151 | | Syringetin-3-O-galactoside | | [M-H]- | | | Phenylpropanoids and polyketides |  |
| 1148 | 4.811 | 303.05197 | | Taxifolin | | [M-H]- | | | Phenylpropanoids and polyketides |  |
| 1149 | 5.859 | 513.23224 | | Telmisartan | | [M-H]- | | | Benzenoids |  |
| 1150 | 5.191 | 535.27301 | | Tenuigenin | | [M-H]- | | | Undefined |  |
| 1151 | 11.894 | 367.35446 | | Tetracosanoic acid | | [M-H]- | | | Lipids and lipid-like molecules |  |
| 1152 | 10.381 | 621.2677 | | Thalsimidine | | [M-H]- | | | Undefined |  |
| 1153 | 0.612 | 135.02957 | | Threonic acid | | [M-H]- | | | Organic oxygen compounds |  |
| 1154 | 5.689 | 593.12811 | | Tiliroside | | [M-H]- | | | Phenylpropanoids and polyketides |  |
| 1155 | 3.455 | 147.04512 | | trans-Cinnamic acid | | [M-H]- | | | Phenylpropanoids and polyketides |  |
| 1156 | 12.371 | 281.24884 | | trans-Vaccenic acid | | [M-H]- | | | Lipids and lipid-like molecules |  |
| 1157 | 3.018 | 421.07745 | | Trehalose-6-phosphate | | [M-H]- | | | Organic oxygen compounds |  |
| 1158 | 10.712 | 393.16858 | | Triamcinolone | | [M-H]- | | | Lipids and lipid-like molecules |  |
| 1159 | 5.217 | 301.03333 | | Tricetin | | [M-H]- | | | Phenylpropanoids and polyketides |  |
| 1160 | 14.419 | 353.34433 | | Tricosanoic acid | | [M-H]- | | | Lipids and lipid-like molecules |  |
| 1161 | 3.32 | 165.05621 | | Tropic acid | | [M-H]- | | | Organic acids and derivatives |  |
| 1162 | 2.881 | 203.08247 | | Tryptophan | | [M-H]- | | | Organoheterocyclic compounds |  |
| 1163 | 5.287 | 609.14722 | | Tulipanin | | [M-2H]- | | | Phenylpropanoids and polyketides |  |
| 1164 | 1.119 | 243.06068 | | Uridine | | [M-H]- | | | Nucleosides, nucleotides, and analogues |  |
| 1165 | 13.834 | 579.43646 | | Uridine diphosphate glucuronic acid | | [M-H]- | | | Nucleosides, nucleotides, and analogues |  |
| 1166 | 10.458 | 455.35162 | | Ursolic acid | | [M-H]- | | | Lipids and lipid-like molecules |  |
| 1167 | 11.357 | 453.33398 | | Ursonic acid | | [M-H]- | | | Undefined |  |
| 1168 | 3.474 | 167.0349 | | Vanillic acid | | [M-H]- | | | Benzenoids |  |
| 1169 | 5.032 | 431.09937 | | Vitexin | | [M-H]- | | | Phenylpropanoids and polyketides |  |
| 1170 | 10.762 | 703.38208 | | Voacamine | | [M-H]- | | | Alkaloids and derivatives |  |
| 1171 | 6.5 | 313.03342 | | Wedelolactone | | [M-H]- | | | Phenylpropanoids and polyketides |  |
| 1172 | 6.829 | 469.2254 | | Xyl(b1-6)Glc(b)-O-octyl | | [M+FA-H]- | | | Lipids and lipid-like molecules |  |
| 1173 | 8.286 | 319.15579 | | Zearalenol | | [M-H]- | | | Phenylpropanoids and polyketides |  |
| 1174 | 8.668 | 321.16815 | | Zeranol | | [M-H]- | | | Phenylpropanoids and polyketides |  |

**Supplementary Table 2.** The list of a total of 528 identified metabolites in kandelia soil samples.

| **No.** | **rt(min)** | | **m/z** | | **Name** | | **Adduct** | **Superclass** | | |
| --- | --- | --- | --- | --- | --- | --- | --- | --- | --- | --- |
| 1 | 328.23035 | 59.4560 | | .beta.-d-glucopyranosiduronic acid, 5-[3-[(2,2,3,3-tetramethylcyclopropyl)carbonyl]-1h-indol-1-yl]pentyl | | [M+H-C6H8O6]+ | | | Lipids and lipid-like molecules |  |
| 2 | 427.27765 | 156.8520 | | .beta.-ecdysone | | [M+H-3H2O]+ | | | Lipids and lipid-like molecules |  |
| 3 | 130.08455 | 101.6540 | | .beta.-homoproline | | [M+H]+ | | | Undefined |  |
| 4 | 397.38043 | 28.8795 | | .beta.-sitosterol | | [M+H-H2O]+ | | | Lipids and lipid-like molecules |  |
| 5 | 86.05878 | 58.6850 | | .gamma.-aminobutyric acid | | [M+H-H2O]+ | | | Organic acids and derivatives |  |
| 6 | 261.21949 | 38.5720 | | .gamma.-linolenoyl ethanolamide | | [M+H-C2H7NO]+ | | | Organic nitrogen compounds |  |
| 7 | 362.32385 | 42.5850 | | (1s,2r-d-erythro-2-n-myristoylamino)-1-phenyl-1-propanol | | [M+H]+ | | | Benzenoids |  |
| 8 | 439.13808 | 442.5750 | | (2r,3s,4s,5r,6r)-5-[(2s,3r,4r)-3,4-dihydroxy-4-(hydroxymethyl)oxolan-2-yl]oxy-2-(hydroxymethyl)-6-(2-phenylethoxy)oxane-3,4-diol | | [M+Na]+ | | | Organic oxygen compounds |  |
| 9 | 362.32360 | 137.1570 | | (r)-(+)-arachidonyl-1'-hydroxy-2'-propylamide | | [M+H]+ | | | Lipids and lipid-like molecules |  |
| 10 | 321.25460 | 164.7370 | | (r)-aminocarnitine | | [2M+H]+ | | | Organic acids and derivatives |  |
| 11 | 480.34131 | 208.3160 | | 1-(1z-hexadecenyl)-sn-glycero-3-phosphocholine | | [M+H]+ | | | Lipids and lipid-like molecules |  |
| 12 | 776.56837 | 56.8360 | | 1-(1z-octadecenyl)-2-(4z,7z,10z,13z,16z,19z-docosahexaenoyl)-sn-glycero-3-phosphoethanolamine | | [M+H]+ | | | Undefined |  |
| 13 | 794.59663 | 146.5505 | | 1-(1z-octadecenyl)-2-(5z,8z,11z,14z-eicosatetraenoyl)-sn-glycero-3-phosphocholine | | [M+H]+ | | | Lipids and lipid-like molecules |  |
| 14 | 772.60831 | 40.8340 | | 1-(1z-octadecenyl)-2-(9z-octadecenoyl)-sn-glycero-3-phosphocholine | | [M+H]+ | | | Lipids and lipid-like molecules |  |
| 15 | 508.37208 | 204.1640 | | 1-(1z-octadecenyl)-sn-glycero-3-phosphocholine | | [M+H]+ | | | Lipids and lipid-like molecules |  |
| 16 | 236.14719 | 389.6250 | | 1-(benzo[d][1,3]dioxol-4-yl)-2-(methylamino)pentan-1-one | | [M+H]+ | | | Benzenoids |  |
| 17 | 534.37558 | 186.2400 | | 1-arachidoyl-2-hydroxy-sn-glycero-3-phosphocholine | | [M+H-H2O]+ | | | Lipids and lipid-like molecules |  |
| 18 | 780.54701 | 40.1660 | | 1-hexadecanoyl-2-octadecadienoyl-sn-glycero-3-phosphocholine | | [M+Na]+ | | | Lipids and lipid-like molecules |  |
| 19 | 768.58175 | 148.1120 | | 1-hexadecyl-2-(5z,8z,11z,14z-eicosatetraenoyl)-sn-glycero-3-phosphocholine | | [M+H]+ | | | Lipids and lipid-like molecules |  |
| 20 | 746.58560 | 138.6795 | | 1-hexadecyl-2-(9z-octadecenoyl)-sn-glycero-3-phosphocholine | | [M+H]+ | | | Lipids and lipid-like molecules |  |
| 21 | 482.35613 | 207.1680 | | 1-hexadecyl-sn-glycero-3-phosphocholine | | [M+H]+ | | | Lipids and lipid-like molecules |  |
| 22 | 242.28335 | 157.4760 | | 1-hexadecylamine | | [M+H]+ | | | Organic nitrogen compounds |  |
| 23 | 263.23527 | 38.1770 | | 1-monolinoleoyl-rac-glycerol | | [M+H-C3H8O3]+ | | | Lipids and lipid-like molecules |  |
| 24 | 468.30537 | 207.9140 | | 1-myristoyl-sn-glycero-3-phosphocholine | | [M+H]+ | | | Lipids and lipid-like molecules |  |
| 25 | 496.37203 | 206.1890 | | 1-o-hexadecyl-2-c-methyl-3-phosphatidylcholine | | [M+H]+ | | | Undefined |  |
| 26 | 546.37445 | 186.0480 | | 1-o-octadecyl-2-o-methyl-sn-glyceryl-3-phosphorylcholine | | [M+Na]+ | | | Lipids and lipid-like molecules |  |
| 27 | 510.38766 | 203.4915 | | 1-o-octadecyl-sn-glyceryl-3-phosphorylcholine | | [M+H]+ | | | Lipids and lipid-like molecules |  |
| 28 | 552.39836 | 194.7360 | | 1-octadecyl-2-acetyl-sn-glycero-3-phosphocholine | | [M+H]+ | | | Lipids and lipid-like molecules |  |
| 29 | 522.35129 | 200.2590 | | 1-oleoyl-sn-glycero-3-phosphocholine | | [M+H]+ | | | Lipids and lipid-like molecules |  |
| 30 | 806.56436 | 147.5190 | | 1-palmitoyl-2-docosahexaenoyl-sn-glycero-3-phosphocholine | | [M+H]+ | | | Lipids and lipid-like molecules |  |
| 31 | 577.51348 | 80.1220 | | 1-palmitoyl-2-oleoyl-sn-glycerol | | [M+H-H2O]+ | | | Lipids and lipid-like molecules |  |
| 32 | 496.33587 | 203.0715 | | 1-palmitoyl-sn-glycero-3-phosphocholine | | [M+H]+ | | | Lipids and lipid-like molecules |  |
| 33 | 313.27140 | 74.9045 | | 1-Palmitoylglycerol | | (M+H-H2O)+ | | | Undefined |  |
| 34 | 482.32028 | 205.1070 | | 1-pentadecanoyl-sn-glycero-3-phosphocholine | | [M+H]+ | | | Lipids and lipid-like molecules |  |
| 35 | 363.13702 | 459.4440 | | 1-piperidinecarboxamide, n-3-pyridinyl-4-[[3-[[5-(trifluoromethyl)-2-pyridinyl]oxy]phenyl]methyl]- | | [M+H-C5H6N2]+ | | | Organoheterocyclic compounds |  |
| 36 | 887.56095 | 34.1920 | | 1-stearoyl-2-arachidonoyl-sn-glycero-3-phospho-(1'-myo-inositol) | | [M+H]+ | | | Lipids and lipid-like molecules |  |
| 37 | 524.37303 | 133.9460 | | 1-Stearoyl-2-hydroxy-sn-glycero-3-phosphocholine | | (M+H)+ | | | Undefined |  |
| 38 | 744.54802 | 153.8345 | | 1-stearoyl-2-linoleoyl-sn-glycero-3-phosphoethanolamine | | [M+H]+ | | | Lipids and lipid-like molecules |  |
| 39 | 746.56341 | 153.1960 | | 1-stearoyl-2-oleoyl-sn-glycero-3-phosphoethanolamine | | [M+H]+ | | | Lipids and lipid-like molecules |  |
| 40 | 788.60374 | 40.7980 | | 1-Stearoyl-2-oleoyl-sn-glycerol 3-phosphocholine (SOPC) | | [M+H]+ | | | Lipids and lipid-like molecules |  |
| 41 | 341.30325 | 35.3820 | | 1-stearoyl-rac-glycerol | | [M+H-H2O]+ | | | Lipids and lipid-like molecules |  |
| 42 | 568.33551 | 196.8400 | | 1-Stearoyl-sn-glycerol 3-phosphocholine | | (M-H+2Na)+ | | | Undefined |  |
| 43 | 524.38956 | 187.3880 | | 1-Stearoyl-sn-glycerol 3-phosphocholine(LPC(18:0)) | | [M+H]+ | | | Lipids and lipid-like molecules |  |
| 44 | 778.53273 | 149.8555 | | 1,2-di-(9z,12z,15z-octadecatrienoyl)-sn-glycero-3-phosphocholine | | [M+H]+ | | | Lipids and lipid-like molecules |  |
| 45 | 878.56413 | 142.3740 | | 1,2-didocosahexaenoyl-sn-glycero-3-phosphocholine | | [M+H]+ | | | Lipids and lipid-like molecules |  |
| 46 | 756.57010 | 139.4120 | | 1,2-dihexadecanoyl-sn-glycero-3-phosphocholine | | [M+Na]+ | | | Lipids and lipid-like molecules |  |
| 47 | 551.50111 | 34.3770 | | 1,2-dihexadecanoyl-sn-glycerol | | [M+H-H2O]+ | | | Lipids and lipid-like molecules |  |
| 48 | 636.45374 | 160.5100 | | 1,2-dimyristoyl-sn-glycero-3-phosphoethanolamine | | [M+H]+ | | | Lipids and lipid-like molecules |  |
| 49 | 786.59716 | 41.9350 | | 1,2-dioleoyl-sn-glycero-3-phosphatidylcholine | | (M+H)+ | | | Undefined |  |
| 50 | 766.52665 | 40.7770 | | 1,2-dioleoyl-sn-glycero-3-phosphoethanolamine | | [M+Na]+ | | | Lipids and lipid-like molecules |  |
| 51 | 810.55256 | 56.3810 | | 1,2-dioleoyl-sn-glycero-3-phosphoethanolamine-n,n-dimethyl | | [M+K]+ | | | Lipids and lipid-like molecules |  |
| 52 | 603.53036 | 77.5120 | | 1,2-dioleoyl-sn-glycerol | | [M+H-H2O]+ | | | Lipids and lipid-like molecules |  |
| 53 | 752.51430 | 38.8120 | | 1,2-dipalmitoleoyl-sn-glycero-3-phosphocholine | | [M+Na]+ | | | Lipids and lipid-like molecules |  |
| 54 | 664.48544 | 159.2595 | | 1,2-dipentadecanoyl-sn-glycero-3-phosphoethanolamine | | [M+H]+ | | | Lipids and lipid-like molecules |  |
| 55 | 259.14958 | 44.4960 | | 1,3-benzenediol, 5-methyl-4-[(1r,6r)-3-methyl-6-(1-methylethenyl)-2-cyclohexen-1-yl]- | | [M+H]+ | | | Lipids and lipid-like molecules |  |
| 56 | 706.39753 | 60.6350 | | 1',4-sophorolactone 6',6-diacetate | | [M+NH4]+ | | | Undefined |  |
| 57 | 339.26558 | 165.2610 | | 11,12-dihydroxy-5z,8z,14z-eicosatrienoic acid | | [M+H]+ | | | Lipids and lipid-like molecules |  |
| 58 | 507.26666 | 36.9380 | | 11.alpha.-hydroxyprogesterone .beta.-d-glucuronide | | [M+H]+ | | | Lipids and lipid-like molecules |  |
| 59 | 284.32889 | 137.7260 | | 15-deoxy-goyazensolide | | [M+K]+ | | | Organoheterocyclic compounds |  |
| 60 | 378.31850 | 168.3550 | | 15(s)-15-methylprostaglandin f2.alpha. ethylamide | | [M+H-H2O]+ | | | Undefined |  |
| 61 | 272.22000 | 111.6290 | | 16-Hydroxypalmitic acid | | [M+H]+ | | | Lipids and lipid-like molecules |  |
| 62 | 485.35564 | 142.7600 | | 18.beta.-glycyrrhetic acid methyl ester | | [M+H]+ | | | Lipids and lipid-like molecules |  |
| 63 | 567.28005 | 206.4400 | | 1h-indole-3-acetamide, 1-(2,2-diethoxyethyl)-2,3-dihydro-n-(4-methylphenyl)-3-[[[(4-methylphenyl)amino]carbonyl]amino]-2-oxo-, (+)- | | [M+Na]+ | | | Benzenoids |  |
| 64 | 558.41072 | 122.3600 | | 2-(21-amino-3,20-dihydroxydocosan-2-yl)oxy-6-(hydroxymethyl)oxane-3,4,5-triol | | [M+Na]+ | | | Lipids and lipid-like molecules |  |
| 65 | 622.29421 | 124.3110 | | 2-(cyclohexylamino)ethanesulfonic acid | | [3M+H]+ | | | Organic nitrogen compounds |  |
| 66 | 215.97599 | 30.0145 | | 2-benzothiazolsulfonic acid | | [M+H]+ | | | Undefined |  |
| 67 | 187.06936 | 397.6815 | | 2-deoxy-d-glucose | | [M+Na]+ | | | Lipids and lipid-like molecules |  |
| 68 | 764.51630 | 151.2920 | | 2-docosahexaenoyl-1-palmitoyl-sn-glycero-3-phosphoethanolamine | | [M+H]+ | | | Lipids and lipid-like molecules |  |
| 69 | 792.54819 | 148.7880 | | 2-docosahexaenoyl-1-stearoyl-sn-glycero-3-phosphoethanolamine | | [M+H]+ | | | Lipids and lipid-like molecules |  |
| 70 | 725.33042 | 64.2420 | | 2-ethylhexyl diphenyl phosphate | | [2M+H]+ | | | Organic acids and derivatives |  |
| 71 | 133.09927 | 36.9010 | | 2-methylamino-1-phenylbutane | | [M+H-CH5N]+ | | | Benzenoids |  |
| 72 | 579.55737 | 167.4155 | | 2-mohsa [dmed-fahfa] | | [M+H]+ | | | Undefined |  |
| 73 | 579.53071 | 34.0485 | | 2-oahma [dmed-fahfa] | | [M+H]+ | | | Undefined |  |
| 74 | 790.53742 | 149.3820 | | 2-oleoyl-1-stearoyl-sn-glycero-3-phosphoserine | | [M+H]+ | | | Lipids and lipid-like molecules |  |
| 75 | 313.27182 | 35.4070 | | 2-palmitoyl-rac-glycerol | | [M+H-H2O]+ | | | Lipids and lipid-like molecules |  |
| 76 | 100.07439 | 50.4840 | | 2-pyrrolidinone, 1-methyl- | | [M+H]+ | | | Organoheterocyclic compounds |  |
| 77 | 246.24098 | 88.3830 | | 2,4,6-tri-tert-butylaniline | | [M+H-CH4]+ | | | Benzenoids |  |
| 78 | 219.17244 | 34.0870 | | 2,6-di-tert-butyl-4-hydroxymethylphenol | | [M+H-H2O]+ | | | Benzenoids |  |
| 79 | 135.07873 | 79.4940 | | 2'-Deoxy-D-ribose | | [M+H]+ | | | Organic oxygen compounds |  |
| 80 | 144.07894 | 51.1440 | | 3-(2-hydroxyethyl)indole | | [M+H-H2O]+ | | | Organoheterocyclic compounds |  |
| 81 | 443.20134 | 29.8225 | | 3-[1-[3-(dimethylamino)propyl]-5-methoxy-1h-indol-3-yl]-4-(1h-indol-3-yl)-1h-pyrrole-2,5-dione | | [M+H]+ | | | Organoheterocyclic compounds |  |
| 82 | 104.06935 | 352.4240 | | 3-aminobutanoic acid | | [M+H]+ | | | Organic acids and derivatives |  |
| 83 | 427.38612 | 162.7300 | | 3-epilupeol | | [M+H]+ | | | Lipids and lipid-like molecules |  |
| 84 | 258.20446 | 119.7600 | | 3-Hydroxydodecanoic acid | | (M+CH3CN+H)+ | | | Organic acids and derivatives |  |
| 85 | 105.06855 | 40.7620 | | 3-methylbenzyl alcohol | | [M+H-H2O]+ | | | Benzenoids |  |
| 86 | 135.07864 | 39.4930 | | 3,4-dimethylbenzaldehyde | | [M+H]+ | | | Benzenoids |  |
| 87 | 363.30823 | 30.0940 | | 3,6,9,12-tetraoxatetracosan-1-ol | | [M+H]+ | | | Organic oxygen compounds |  |
| 88 | 137.06909 | 269.8795 | | 4-aminobenzamide | | [M+H]+ | | | Benzenoids |  |
| 89 | 121.06316 | 41.2190 | | 4-hydroxyphenethyl alcohol | | [M+H-H2O]+ | | | Benzenoids |  |
| 90 | 127.04876 | 286.4870 | | 4-imidazoleacetic acid | | [M+H]+ | | | Organoheterocyclic compounds |  |
| 91 | 139.04857 | 302.3230 | | 4-imidazoleacrylic acid | | [M+H]+ | | | Organoheterocyclic compounds |  |
| 92 | 353.07736 | 143.7060 | | 4-methylumbelliferyl .beta.-d-glucuronide | | [M+H]+ | | | Phenylpropanoids and polyketides |  |
| 93 | 210.07737 | 205.6110 | | 4-morpholinopropanesulfonic acid | | [M+H]+ | | | Organoheterocyclic compounds |  |
| 94 | 538.36983 | 55.9230 | | 4-quinazolinamine, 7-[2-[2-(dimethylamino)ethoxy]ethoxy]-2-(hexahydro-4-methyl-1h-1,4-diazepin-1-yl)-6-methoxy-n-(1-methyl-4-piperidinyl)- | | [M+Na]+ | | | Organoheterocyclic compounds |  |
| 95 | 486.35398 | 125.5450 | | 4-quinazolinamine, 7-[3-(dimethylamino)propoxy]-2-(hexahydro-4-methyl-1h-1,4-diazepin-1-yl)-6-methoxy-n-(1-methyl-4-piperidinyl)- | | [M+H]+ | | | Organoheterocyclic compounds |  |
| 96 | 353.02375 | 455.7050 | | 4,5-dihydro-4,5-dioxo-1h-pyrrolo[2,3-f]quinoline-2,7,9-tricarboxylic acid | | [M+Na]+ | | | Organoheterocyclic compounds |  |
| 97 | 453.16363 | 40.6210 | | 4'-o-.beta.-d-glucosyl-5-o-methylvisamminol | | [M+H]+ | | | Organoheterocyclic compounds |  |
| 98 | 129.10061 | 423.8740 | | 6-methyl-5-hepten-2-ol | | [M+H]+ | | | Lipids and lipid-like molecules |  |
| 99 | 429.21380 | 138.4860 | | 6.alpha.-hydroxybudesonide | | [M+H-H2O]+ | | | Lipids and lipid-like molecules |  |
| 100 | 415.20067 | 150.0120 | | 7-hydroxymitragynine | | [M+H]+ | | | Undefined |  |
| 101 | 293.20895 | 40.8795 | | 9-hydroperoxy-10e,12z,15z-octadecatrienoic acid | | [M+H-H2O]+ | | | Lipids and lipid-like molecules |  |
| 102 | 361.24117 | 134.8735 | | 9s,15s-dihydroxy-5z,13e-prostadienoic acid | | [M+Na]+ | | | Lipids and lipid-like molecules |  |
| 103 | 347.09212 | 415.0190 | | Acetohexamide | | [M+Na]+ | | | Organic oxygen compounds |  |
| 104 | 204.12213 | 323.6740 | | Acetylcarnitine | | [M+H]+ | | | Lipids and lipid-like molecules |  |
| 105 | 136.06015 | 174.9300 | | Adenine | | [M+H]+ | | | Organoheterocyclic compounds |  |
| 106 | 268.10186 | 185.7470 | | Adenosine | | [M+H]+ | | | Nucleosides, nucleotides, and analogues |  |
| 107 | 422.32471 | 158.8280 | | Agelasinee/f | | [M]+ | | | Lipids and lipid-like molecules |  |
| 108 | 132.10015 | 371.7350 | | Alanine betaine | | [M+H]+ | | | Organic acids and derivatives |  |
| 109 | 296.25713 | 39.4270 | | alpha-Linolenic acid | | (M+NH4)+ | | | Lipids and lipid-like molecules |  |
| 110 | 278.18821 | 81.3720 | | Amitriptyline | | (M+H)+ | | | Undefined |  |
| 111 | 837.46852 | 72.9395 | | Anabaenopeptin b | | [M+H]+ | | | Organic acids and derivatives |  |
| 112 | 584.42634 | 121.4155 | | Antheraxanthin | | [M]+ | | | Lipids and lipid-like molecules |  |
| 113 | 428.26162 | 194.2990 | | Arachidonoyl ethanolamide phosphate | | [M+H]+ | | | Organic acids and derivatives |  |
| 114 | 350.28171 | 137.0050 | | Arachidonoyl-2'-fluoroethylamide | | [M+H]+ | | | Lipids and lipid-like molecules |  |
| 115 | 332.27772 | 138.1510 | | Arachidonoyl-n,n-dimethylamide | | [M+H]+ | | | Lipids and lipid-like molecules |  |
| 116 | 784.57822 | 150.1980 | | Arachidonoylthiophosphorylcholine | | [M+H]+ | | | Organic nitrogen compounds |  |
| 117 | 356.35054 | 63.7610 | | Arachidoyl ethanolamide | | [M+H]+ | | | Organic nitrogen compounds |  |
| 118 | 557.21568 | 121.0600 | | Arctiin | | [M+Na]+ | | | Undefined |  |
| 119 | 210.13177 | 259.3440 | | Arg-Ala | | (M+H-2H2O)+ | | | Undefined |  |
| 120 | 175.11693 | 444.4775 | | Arginine | | [M+H]+ | | | Organic acids and derivatives |  |
| 121 | 189.08542 | 512.0270 | | Asp-Asp-Lys | | [M+2H]2+ | | | Organic acids and derivatives |  |
| 122 | 235.09091 | 514.6610 | | Asp-Thr | | [M+H]+ | | | Organic acids and derivatives |  |
| 123 | 587.22416 | 149.3525 | | Baccatin iii | | [M+H]+ | | | Lipids and lipid-like molecules |  |
| 124 | 409.15930 | 41.3610 | | Beclomethasone | | [M+H]+ | | | Lipids and lipid-like molecules |  |
| 125 | 503.22228 | 197.1300 | | Beclomethasone dipropionate | | [M+H-H2O]+ | | | Lipids and lipid-like molecules |  |
| 126 | 329.09346 | 398.1860 | | Benz[a]anthracene-1,7,12(2h)-trione, 3,4-dihydro-8-hydroxy-3-methyl-, (3s)- | | [M+Na]+ | | | Undefined |  |
| 127 | 304.29772 | 132.4900 | | Benzalkonium chloride (c12) | | [M]+ | | | Benzenoids |  |
| 128 | 815.32492 | 104.1210 | | Benzoic acid, 2-(diphenylphosphino)-4-[21-[(3as,4s,6ar)-hexahydro-2-oxo-1h-thieno[3,4-d]imidazol-4-yl]-1,17-dioxo-6,9,12-trioxa-2,16-diazaheneicos-1-yl]-, methyl ester | | [M+Na]+ | | | Benzenoids |  |
| 129 | 335.11725 | 223.5780 | | Benzyl butyl phthalate | | [M+Na]+ | | | Benzenoids |  |
| 130 | 118.08581 | 292.3270 | | Betaine | | [M+H]+ | | | Organic acids and derivatives |  |
| 131 | 348.21210 | 124.9605 | | Bisoprolol | | [M+Na]+ | | | Benzenoids |  |
| 132 | 381.34595 | 28.7720 | | Brassicasterol | | [M+H-H2O]+ | | | Lipids and lipid-like molecules |  |
| 133 | 288.28793 | 74.9190 | | C17-sphinganine | | [M+H]+ | | | Organic nitrogen compounds |  |
| 134 | 381.27547 | 33.6995 | | Cabergoline | | [M+H-C3H5NO]+ | | | Alkaloids and derivatives |  |
| 135 | 195.08598 | 51.5030 | | Caffeine | | [M+H]+ | | | Organoheterocyclic compounds |  |
| 136 | 600.42120 | 151.3080 | | Capsorubin | | [M]+ | | | Lipids and lipid-like molecules |  |
| 137 | 116.06910 | 337.7950 | | Captopril | | [M+H-C4H6SO]+ | | | Organic acids and derivatives |  |
| 138 | 615.34654 | 174.6210 | | Carbenoxolone | | [M-H+2Na]+ | | | Lipids and lipid-like molecules |  |
| 139 | 565.51405 | 81.3800 | | Cer 16:1-d7 (d18:1-d7/16:1) | | [M+Na]+ | | | Undefined |  |
| 140 | 505.22064 | 197.0360 | | Chlorhexidine | | [M+H]+ | | | Benzenoids |  |
| 141 | 361.01670 | 392.7770 | | Chloropropylate | | [M+Na]+ | | | Benzenoids |  |
| 142 | 369.34890 | 28.2905 | | Cholesterol | | [M+H-H2O]+ | | | Lipids and lipid-like molecules |  |
| 143 | 104.10560 | 403.1570 | | Choline | | [M]+ | | | Organic nitrogen compounds |  |
| 144 | 617.34470 | 175.4360 | | Cimicifugoside h 2 | | [M+H-H2O]+ | | | Lipids and lipid-like molecules |  |
| 145 | 307.11294 | 85.7570 | | Cimifugin | | [M+H]+ | | | Organoheterocyclic compounds |  |
| 146 | 443.22958 | 136.5360 | | Cinobufagin | | [M+H]+ | | | Lipids and lipid-like molecules |  |
| 147 | 187.09206 | 59.0470 | | Coenzyme q1 | | [M+H-C2H8O2]+ | | | Lipids and lipid-like molecules |  |
| 148 | 589.23999 | 118.2530 | | Convallatoxin | | [M+K]+ | | | Lipids and lipid-like molecules |  |
| 149 | 347.21594 | 56.8000 | | Cortexolone | | [M+H]+ | | | Lipids and lipid-like molecules |  |
| 150 | 293.13397 | 111.5820 | | Coumatetralyl | | [M+H]+ | | | Phenylpropanoids and polyketides |  |
| 151 | 599.40458 | 36.9360 | | Crassostreaxanthin a | | [M+H]+ | | | Lipids and lipid-like molecules |  |
| 152 | 621.40896 | 28.9855 | | Crassostreaxanthin b | | [M+Na]+ | | | Lipids and lipid-like molecules |  |
| 153 | 132.07517 | 364.6190 | | Creatine | | [M+H]+ | | | Organic acids and derivatives |  |
| 154 | 114.06465 | 182.4280 | | Creatinine | | [M+H]+ | | | Organic acids and derivatives |  |
| 155 | 605.23425 | 46.3305 | | Cumyluron | | [2M+H]+ | | | Benzenoids |  |
| 156 | 705.03626 | 456.1830 | | Cyazofamid | | [M+Na]+ | | | Organoheterocyclic compounds |  |
| 157 | 549.30539 | 167.0035 | | Cymarin | | [M+H]+ | | | Lipids and lipid-like molecules |  |
| 158 | 112.04906 | 222.6115 | | Cytosine | | [M+H]+ | | | Organoheterocyclic compounds |  |
| 159 | 130.04824 | 311.1320 | | D-pyroglutamic acid | | [M+H]+ | | | Organic acids and derivatives |  |
| 160 | 316.29762 | 133.3130 | | Dehydrophytosphingosine (not validated, isomer of 1677) | | [M+H]+ | | | Organic nitrogen compounds |  |
| 161 | 400.37499 | 65.9130 | | Demissidine | | [M+H]+ | | | Undefined |  |
| 162 | 146.11573 | 396.0520 | | Deoxycarnitine | | [M+H]+ | | | Lipids and lipid-like molecules |  |
| 163 | 279.15724 | 32.8570 | | Dibutyl phthalate | | [M+H]+ | | | Benzenoids |  |
| 164 | 182.18860 | 176.0540 | | Dicyclohexylamine | | [M+H]+ | | | Organic nitrogen compounds |  |
| 165 | 515.38852 | 62.2460 | | Didodecyl 3,3'-thiodipropionate | | [M+H]+ | | | Organic acids and derivatives |  |
| 166 | 399.36981 | 131.9760 | | Dihydrotachysterol | | [M+H]+ | | | Lipids and lipid-like molecules |  |
| 167 | 613.47775 | 163.9460 | | Dilinolenin (9c,12c,15c) | | [M+H]+ | | | Lipids and lipid-like molecules |  |
| 168 | 391.28113 | 33.6570 | | Dioctyl phthalate | | [M+H]+ | | | Benzenoids |  |
| 169 | 149.02168 | 32.3940 | | Dl-4-hydroxy-3-methoxymandelic acid | | [M+H-CH6O2]+ | | | Benzenoids |  |
| 170 | 148.05864 | 417.4040 | | DL-Glutamic acid | | [M+H]+ | | | Organic acids and derivatives |  |
| 171 | 146.11581 | 288.2700 | | Dl-norleucine methyl ester | | [M+H]+ | | | Organic acids and derivatives |  |
| 172 | 372.28725 | 92.5970 | | Docosahexaenoyl ethanolamide | | [M+H]+ | | | Organic nitrogen compounds |  |
| 173 | 340.35426 | 65.2045 | | Docosanamide | | [M+H]+ | | | Lipids and lipid-like molecules |  |
| 174 | 662.49503 | 58.9820 | | Dodecanamide, n-[(1s,2r)-2-hydroxy-1-(hydroxymethyl)heptadecyl]-12-[(7-nitro-2,1,3-benzoxadiazol-4-yl)amino]- | | [M+H]+ | | | Lipids and lipid-like molecules |  |
| 175 | 216.19365 | 51.1010 | | Dodecanoic acid, 12-[[(tricyclo[3.3.1.13,7]dec-1-ylamino)carbonyl]amino]- | | [M+H-C11H15NO]+ | | | Lipids and lipid-like molecules |  |
| 176 | 318.23853 | 39.8990 | | Drofenine | | [M+H]+ | | | Undefined |  |
| 177 | 143.07980 | 353.0140 | | Ectoine | | [M+H]+ | | | Organic acids and derivatives |  |
| 178 | 464.27521 | 160.1440 | | Emetine | | [M+H-NH3]+ | | | Undefined |  |
| 179 | 321.12809 | 79.4900 | | Enoxacin | | [M+H]+ | | | Organoheterocyclic compounds |  |
| 180 | 114.08983 | 19.9630 | | epsilon-Caprolactam | | (M+H)+ | | | Undefined |  |
| 181 | 338.34123 | 37.2600 | | Erucamide | | [M+H]+ | | | Lipids and lipid-like molecules |  |
| 182 | 413.38563 | 127.5330 | | Erythrodiol | | [M+H-CH2O]+ | | | Lipids and lipid-like molecules |  |
| 183 | 398.97306 | 292.5880 | | Ethiprole | | [M+H+2i]+ | | | Organoheterocyclic compounds |  |
| 184 | 293.09884 | 455.6620 | | Ethylenediaminetetraacetic acid | | [M+H]+ | | | Organic acids and derivatives |  |
| 185 | 471.17974 | 130.3580 | | Evodin | | [M+H]+ | | | Lipids and lipid-like molecules |  |
| 186 | 274.27226 | 308.6560 | | Fenpropidin | | [M+H]+ | | | Benzenoids |  |
| 187 | 420.95331 | 415.0450 | | Fipronil sulfide | | [M+H]+ | | | Organoheterocyclic compounds |  |
| 188 | 423.23120 | 117.9080 | | Fludrocortisone | | [M+H]+ | | | Lipids and lipid-like molecules |  |
| 189 | 399.17585 | 28.5930 | | Fluorometholone | | [M+Na]+ | | | Lipids and lipid-like molecules |  |
| 190 | 309.16342 | 506.1910 | | Fructoselysine | | [M+H]+ | | | Organic acids and derivatives |  |
| 191 | 172.13178 | 39.0430 | | Gabapentin | | [M+H]+ | | | Organic acids and derivatives |  |
| 192 | 359.12126 | 462.8320 | | Gardenin b | | [M+H]+ | | | Phenylpropanoids and polyketides |  |
| 193 | 411.11925 | 19.5450 | | Geniposide | | [M+Na]+ | | | Lipids and lipid-like molecules |  |
| 194 | 407.12537 | 398.8770 | | Ginkgolide b | | [M+H-H2O]+ | | | Undefined |  |
| 195 | 767.50287 | 157.0005 | | Ginsenoside rg3 (r-form) | | [M+H-H2O]+ | | | Lipids and lipid-like molecules |  |
| 196 | 605.42623 | 166.8480 | | Ginsenoside rh2 | | [M+H-H2O]+ | | | Lipids and lipid-like molecules |  |
| 197 | 789.47110 | 76.9960 | | Ginsenoside rk1 | | [M+Na]+ | | | Lipids and lipid-like molecules |  |
| 198 | 613.15555 | 510.3810 | | Glutathione, oxidized | | [M+H]+ | | | Organic acids and derivatives |  |
| 199 | 194.11341 | 45.1270 | | Gly-Thr | | (M+NH4)+ | | | Undefined |  |
| 200 | 488.39123 | 143.7060 | | Glycerol tricaprylate | | [M+NH4]+ | | | Lipids and lipid-like molecules |  |
| 201 | 258.10807 | 402.9570 | | Glycerophosphocholine | | [M+H]+ | | | Lipids and lipid-like molecules |  |
| 202 | 357.27677 | 165.2710 | | Glycodeoxycholic acid | | [M+H-C2H7O3N]+ | | | Lipids and lipid-like molecules |  |
| 203 | 501.19463 | 462.8230 | | Gossypol | | [M+H-H2O]+ | | | Lipids and lipid-like molecules |  |
| 204 | 270.27716 | 168.8380 | | Heptadecasphinganine | | [M+H-H2O]+ | | | Organic nitrogen compounds |  |
| 205 | 340.39162 | 129.2055 | | Hexetidine | | [M+H]+ | | | Undefined |  |
| 206 | 337.04645 | 451.9850 | | Hydroxyflutamide | | (M-H+2Na)+ | | | Undefined |  |
| 207 | 137.04414 | 188.0680 | | Hypoxanthine | | [M+H]+ | | | Organoheterocyclic compounds |  |
| 208 | 465.28505 | 83.2005 | | Indinavir | | [M+H-C9H11NO]+ | | | Organic acids and derivatives |  |
| 209 | 130.06335 | 51.9530 | | Indole-3-acetamide | | [M+H-CH3ON]+ | | | Organoheterocyclic compounds |  |
| 210 | 194.11588 | 352.9800 | | Isoproterenol | | [M+H-H2O]+ | | | Benzenoids |  |
| 211 | 175.11714 | 523.5340 | | L-Arginine | | (M+H)+ | | | Organic acids and derivatives |  |
| 212 | 162.11070 | 373.8970 | | L-carnitine | | [M+H]+ | | | Organic nitrogen compounds |  |
| 213 | 144.06370 | 50.4150 | | L-glutamic acid, dimethyl ester | | [M+H-CH4O]+ | | | Organic acids and derivatives |  |
| 214 | 308.08665 | 447.7375 | | L-glutathione, reduced | | [M+H]+ | | | Organic acids and derivatives |  |
| 215 | 400.36237 | 165.6495 | | L-palmitoylcarnitine | | [M+H]+ | | | Lipids and lipid-like molecules |  |
| 216 | 365.10265 | 413.8090 | | Lactose | | [M+Na]+ | | | Organic oxygen compounds |  |
| 217 | 365.10214 | 448.2345 | | Lactulose | | [M+Na]+ | | | Lipids and lipid-like molecules |  |
| 218 | 535.26280 | 38.2005 | | Lappaconitine | | [M+H-CH6O2]+ | | | Lipids and lipid-like molecules |  |
| 219 | 343.29311 | 177.9180 | | Lauramidopropylbetaine | | [M]+ | | | Organic acids and derivatives |  |
| 220 | 288.25078 | 41.4220 | | Lauric acid diethanolamide | | [M+H]+ | | | Lipids and lipid-like molecules |  |
| 221 | 166.10545 | 308.3445 | | Leu-Ala-Lys | | [M+2H]2+ | | | Organic acids and derivatives |  |
| 222 | 317.21589 | 526.6040 | | Leu-Gly-Lys | | [M+H]+ | | | Organic acids and derivatives |  |
| 223 | 427.30070 | 39.7140 | | Leupeptin | | [M+H]+ | | | Organic acids and derivatives |  |
| 224 | 245.22441 | 38.2450 | | Linoelaidic acid | | [M+H-2H2O]+ | | | Lipids and lipid-like molecules |  |
| 225 | 324.28716 | 38.8065 | | Linoleoyl ethanolamide | | [M+H]+ | | | Organic nitrogen compounds |  |
| 226 | 427.22689 | 118.4800 | | Lovatatin | | [M+Na]+ | | | Organoheterocyclic compounds |  |
| 227 | 520.35933 | 188.2960 | | Lpc 18:2 | | [M+H]+ | | | Lipids and lipid-like molecules |  |
| 228 | 527.15346 | 460.3700 | | Maltotriose | | [M+Na]+ | | | Organic oxygen compounds |  |
| 229 | 533.25033 | 38.0070 | | Manumycin a | | [M+H-H2O]+ | | | Organic oxygen compounds |  |
| 230 | 127.07118 | 190.5155 | | Melamine | | [M+H]+ | | | Organoheterocyclic compounds |  |
| 231 | 437.19021 | 40.3640 | | Met-Met-Arg | | [M+H]+ | | | Organic acids and derivatives |  |
| 232 | 138.05331 | 237.2495 | | Methylpicolinate | | [M+H]+ | | | Organoheterocyclic compounds |  |
| 233 | 561.30640 | 148.8100 | | Mianserin n-oxide | | [2M+H]+ | | | Organoheterocyclic compounds |  |
| 234 | 188.12632 | 231.1520 | | Molinate | | [M+H]+ | | | Undefined |  |
| 235 | 311.25554 | 55.8430 | | Monopalmitolein (9c) | | [M+H-H2O]+ | | | Lipids and lipid-like molecules |  |
| 236 | 384.26146 | 181.2190 | | Myriocin | | [M+H-H2O]+ | | | Lipids and lipid-like molecules |  |
| 237 | 258.27874 | 157.0480 | | Myristamine oxide | | [M+H]+ | | | Organic nitrogen compounds |  |
| 238 | 231.11864 | 58.9910 | | N-(m-trifluoromethylphenyl)piperazine | | [M+H]+ | | | Organoheterocyclic compounds |  |
| 239 | 620.28550 | 124.9615 | | N-[(2s)-2-[[(1z)-1-methyl-3-oxo-3-[4-(trifluoromethyl)phenyl]-1-propen-1-yl]amino]-3-[4-[2-(5-methyl-2-phenyl-4-oxazolyl)ethoxy]phenyl]propyl]propanamide | | [M+H]+ | | | Organoheterocyclic compounds |  |
| 240 | 352.25029 | 134.1620 | | N-3-oxohexadec-11z-enoyl-l-homoserine lactone | | [M+H]+ | | | Organic acids and derivatives |  |
| 241 | 160.09515 | 313.6135 | | N-acetyl-dl-valine | | [M+H]+ | | | Organic acids and derivatives |  |
| 242 | 198.08540 | 328.7530 | | N-acetylhistidine | | [M+H]+ | | | Organic acids and derivatives |  |
| 243 | 306.27645 | 154.8840 | | N-acetylsphingosine | | [M+H-2H2O]+ | | | Lipids and lipid-like molecules |  |
| 244 | 684.45180 | 157.4300 | | N-demethylerythromycin | | [M+H-2H2O]+ | | | Organic oxygen compounds |  |
| 245 | 186.21980 | 165.5980 | | N-dodecylamine | | [M+H]+ | | | Organic nitrogen compounds |  |
| 246 | 484.46881 | 35.0385 | | N-lauroyl-d-erythro-sphinganine | | [M+H]+ | | | Lipids and lipid-like molecules |  |
| 247 | 512.50162 | 35.8340 | | N-myristoylsphinganine | | [M+H]+ | | | Lipids and lipid-like molecules |  |
| 248 | 474.37543 | 146.2990 | | N-nervonoyltaurine | | [M+H]+ | | | Lipids and lipid-like molecules |  |
| 249 | 270.31465 | 154.6580 | | N-octadecylamine | | [M+H]+ | | | Organic nitrogen compounds |  |
| 250 | 390.35490 | 74.9020 | | N-octanoylsphingosine | | [M+H-2H2O]+ | | | Lipids and lipid-like molecules |  |
| 251 | 528.32704 | 160.1910 | | N-octanoylsphingosine-1-phosphate | | [M+Na]+ | | | Lipids and lipid-like molecules |  |
| 252 | 343.10839 | 357.8120 | | N-oxyclozapine | | [M+H]+ | | | Organoheterocyclic compounds |  |
| 253 | 256.26199 | 38.6470 | | N-palmitoyl-d-sphingosine | | [M+H-C18H34O2]+ | | | Lipids and lipid-like molecules |  |
| 254 | 568.56475 | 35.9600 | | N-stearoylsphinganine | | [M+H]+ | | | Lipids and lipid-like molecules |  |
| 255 | 239.08688 | 378.8790 | | N.alpha.-z-d-2,3-diaminopropionic acid | | [M+H]+ | | | Benzenoids |  |
| 256 | 345.08431 | 268.7455 | | Nevadensin | | [M+H]+ | | | Phenylpropanoids and polyketides |  |
| 257 | 123.05372 | 68.0920 | | Niacinamide | | [M+H]+ | | | Organoheterocyclic compounds |  |
| 258 | 163.12094 | 88.9680 | | Nicotine | | [M+H]+ | | | Organoheterocyclic compounds |  |
| 259 | 955.53295 | 94.7060 | | Notoginsenoside r1 | | [M+Na]+ | | | Lipids and lipid-like molecules |  |
| 260 | 282.27994 | 38.0070 | | Oleamide | | [M+H]+ | | | Lipids and lipid-like molecules |  |
| 261 | 518.40139 | 193.0000 | | Orlistat | | [M+Na]+ | | | Organic acids and derivatives |  |
| 262 | 245.13370 | 53.0210 | | Osthole | | [M+H]+ | | | Undefined |  |
| 263 | 607.24954 | 42.9085 | | Ouabain | | [M+Na]+ | | | Lipids and lipid-like molecules |  |
| 264 | 336.24862 | 40.6960 | | Oxeladin | | [M+H]+ | | | Benzenoids |  |
| 265 | 300.28760 | 76.9960 | | Palmitoyl ethanolamide | | (M+H)+ | | | Undefined |  |
| 266 | 703.56953 | 187.7700 | | Palmitoyl sphingomyelin | | [M+H]+ | | | Lipids and lipid-like molecules |  |
| 267 | 826.53212 | 146.3400 | | PC(20:5(5Z,8Z,11Z,14Z,17Z)/20:5(5Z,8Z,11Z,14Z,17Z)) | | M+ | | | Undefined |  |
| 268 | 432.32725 | 199.0955 | | Peimine | | [M+H]+ | | | Lipids and lipid-like molecules |  |
| 269 | 309.22529 | 41.7360 | | Pentapropylene glycol | | [M+H]+ | | | Organic oxygen compounds |  |
| 270 | 263.12207 | 372.6510 | | Phe-pro | | [M+H]+ | | | Organic acids and derivatives |  |
| 271 | 341.26458 | 31.7540 | | Phenol, 5-(1,1-dimethylheptyl)-2-[(1r,2r,5r)-5-hydroxy-2-(3-hydroxypropyl)cyclohexyl]-, rel-(+)- | | [M+H-2H2O]+ | | | Benzenoids |  |
| 272 | 215.06822 | 54.3720 | | Phenyl salicylate | | [M+H]+ | | | Undefined |  |
| 273 | 593.27122 | 73.3140 | | Pheophorbide a | | [M+H]+ | | | Organoheterocyclic compounds |  |
| 274 | 389.98034 | 440.9680 | | Phosalone | | [M+Na]+ | | | Organoheterocyclic compounds |  |
| 275 | 748.57849 | 153.6870 | | Phosphatidylethanolamine | | [M+H]+ | | | Lipids and lipid-like molecules |  |
| 276 | 685.33496 | 62.0660 | | Phosphonic acid, p-[(3r)-3-amino-4-[(3-hexylphenyl)amino]-4-oxobutyl]- | | [2M+H]+ | | | Organic acids and derivatives |  |
| 277 | 717.46085 | 86.4580 | | Pleiokomenine a | | [M+H]+ | | | Undefined |  |
| 278 | 299.30271 | 42.1690 | | Pristanic acid | | [M+H]+ | | | Lipids and lipid-like molecules |  |
| 279 | 487.28344 | 116.6910 | | Pristimerin | | [M+Na]+ | | | Lipids and lipid-like molecules |  |
| 280 | 302.30464 | 70.6710 | | Pro-Trp | | [M+H]+ | | | Organic nitrogen compounds |  |
| 281 | 513.26952 | 26.3680 | | Proscillaridin a | | [M+H-H2O]+ | | | Lipids and lipid-like molecules |  |
| 282 | 415.34979 | 236.2740 | | Putative (3-hydroxyheptadecanoyl)lysine | | [M+H]+ | | | Organic acids and derivatives |  |
| 283 | 550.40532 | 184.7625 | | Pyrrolidinium, 1-[(7r)-7-(acetyloxy)-4-hydroxy-4-oxido-3,5,9-trioxa-4-phosphapentacos-1-yl]-1-methyl-, inner salt | | [M+H]+ | | | Undefined |  |
| 284 | 317.06301 | 446.4770 | | Quercetin 3'-methyl ether | | [M+H]+ | | | Phenylpropanoids and polyketides |  |
| 285 | 453.29522 | 161.1755 | | Repaglinide | | [M+H]+ | | | Organoheterocyclic compounds |  |
| 286 | 609.26578 | 104.0660 | | Reserpine | | [M+H]+ | | | Undefined |  |
| 287 | 619.28406 | 32.4610 | | Salannin | | [M+Na]+ | | | Lipids and lipid-like molecules |  |
| 288 | 322.18884 | 147.1670 | | Sisomicin | | [M+H-C3H12O4N]+ | | | Organic oxygen compounds |  |
| 289 | 428.37200 | 164.1165 | | Sorbitane monooleate - polysorbate 20 in-source fragment | | [M+H]+ | | | Lipids and lipid-like molecules |  |
| 290 | 402.35476 | 165.9010 | | Sorbitane monopalmitate - polysorbate 40 in-source fragment | | [M+H]+ | | | Lipids and lipid-like molecules |  |
| 291 | 300.28730 | 143.7390 | | Sphingosine | | [M+H]+ | | | Organic nitrogen compounds |  |
| 292 | 351.07850 | 143.3340 | | Spinosine | | [M+H-C8H18O9]+ | | | Phenylpropanoids and polyketides |  |
| 293 | 144.10031 | 290.5630 | | Stachydrine | | [M+H]+ | | | Organic acids and derivatives |  |
| 294 | 344.31318 | 55.7210 | | Stearamide | | (M+CH3COO+2H)+ | | | Lipids and lipid-like molecules |  |
| 295 | 294.24107 | 38.8070 | | Stearidonic Acid | | (M+NH4)+ | | | Undefined |  |
| 296 | 372.23999 | 143.7180 | | Tamoxifen | | [M+H]+ | | | Phenylpropanoids and polyketides |  |
| 297 | 563.32106 | 157.9515 | | Tandutinib | | [M+H]+ | | | Organoheterocyclic compounds |  |
| 298 | 413.31661 | 152.9785 | | Testosterone cypionate | | [M+H]+ | | | Lipids and lipid-like molecules |  |
| 299 | 214.25124 | 126.2895 | | Tetradecylamine | | [M+H]+ | | | Organic nitrogen compounds |  |
| 300 | 209.13642 | 58.7740 | | Tetraethylene glycol monomethyl ether | | [M+H]+ | | | Organic oxygen compounds |  |
| 301 | 223.15192 | 52.5450 | | Tetraglyme | | [M+H]+ | | | Organic oxygen compounds |  |
| 302 | 655.31040 | 31.3080 | | Tetrahydrogambogic acid | | [M+Na]+ | | | Organoheterocyclic compounds |  |
| 303 | 758.56464 | 41.4335 | | Thioetheramide-PC | | (M+Na)+ | | | Undefined |  |
| 304 | 645.10015 | 454.9390 | | Thymidine 5'-monophosphate | | [2M+H]+ | | | Nucleosides, nucleotides, and analogues |  |
| 305 | 127.04877 | 106.4120 | | Thymine | | [M+H]+ | | | Organoheterocyclic compounds |  |
| 306 | 276.27858 | 42.7020 | | Trans-hydroxyperhexiline | | [M+H-H2O]+ | | | Organic oxygen compounds |  |
| 307 | 265.25042 | 94.8090 | | trans-Vaccenic acid | | (M+H-H2O)+ | | | Lipids and lipid-like molecules |  |
| 308 | 457.20826 | 150.7385 | | Triamcinolone acetonide | | [M+Na]+ | | | Lipids and lipid-like molecules |  |
| 309 | 150.11069 | 188.3900 | | Triethanolamine | | [M+H]+ | | | Organic nitrogen compounds |  |
| 310 | 179.12589 | 52.5930 | | Triglyme | | [M+H]+ | | | Organic oxygen compounds |  |
| 311 | 138.05324 | 309.2580 | | Trigonelline | | [M+H]+ | | | Alkaloids and derivatives |  |
| 312 | 193.14165 | 43.9290 | | Tripropylene glycol | | [M+H]+ | | | Organic oxygen compounds |  |
| 313 | 122.07961 | 315.2500 | | Tris(hydroxymethyl)aminomethane | | [M+H]+ | | | Organic nitrogen compounds |  |
| 314 | 139.04860 | 190.8660 | | Urocanate | | [M+H]+ | | | Organoheterocyclic compounds |  |
| 315 | 410.32306 | 164.0250 | | Ursodeoxycholic acid | | [M+NH4]+ | | | Lipids and lipid-like molecules |  |
| 316 | 387.23413 | 118.3980 | | Valerylfentanyl | | [M+Na]+ | | | Organoheterocyclic compounds |  |
| 317 | 709.33595 | 61.7775 | | Valganciclovir | | [2M+H]+ | | | Organic acids and derivatives |  |
| 318 | 456.99590 | 451.0020 | | Vindoline | | [M+H]+ | | | Undefined |  |
| 319 | 119.08379 | 39.9470 | | Zerumbone | | [M+H-C6H12O]+ | | | Lipids and lipid-like molecules |  |
| 320 | 277.21638 | 51.8685 | | .gamma.-linolenic acid | | [M-H]- | | | Lipids and lipid-like molecules |  |
| 321 | 411.10534 | 45.9600 | | (-)-riboflavin | | [M+Cl]- | | | Organoheterocyclic compounds |  |
| 322 | 687.37434 | 59.9600 | | (.+/-.)-11-nor-.delta.9-tetrahydrocannabinol-9-carboxylic acid | | [2M-H]- | | | Organoheterocyclic compounds |  |
| 323 | 215.06621 | 408.3285 | | (+)-6-aminopenicillanic acid | | [M-H]- | | | Organic acids and derivatives |  |
| 324 | 289.08573 | 45.7350 | | (+)-catechin | | [M-H]- | | | Phenylpropanoids and polyketides |  |
| 325 | 327.21646 | 167.1750 | | (10e,15z)-9,12,13-trihydroxyoctadeca-10,15-dienoic acid | | [M-H]- | | | Lipids and lipid-like molecules |  |
| 326 | 347.19723 | 76.3465 | | (1s,4ar,5s)-5-[(e)-5-methoxy-3-methyl-5-oxopent-3-enyl]-1,4a-dimethyl-6-methylidene-3,4,5,7,8,8a-hexahydro-2h-naphthalene-1-carboxylic acid | | [M-H]- | | | Lipids and lipid-like molecules |  |
| 327 | 665.39302 | 155.5565 | | (2e,4e)-12-[(10e,12e)-13-carboxy-3-hydroxy-2-(hydroxymethyl)-8,10,12-trimethyltrideca-10,12-dienoyl]oxy-13-(hydroxymethyl)-3,5,7-trimethyltetradeca-2,4-dienedioic acid | | [M-H]- | | | Lipids and lipid-like molecules |  |
| 328 | 425.25660 | 30.0975 | | (2e,6e,10e)-13-[(2r)-6-hydroxy-2,8-dimethyl-3,4-dihydrochromen-2-yl]-2,6,10-trimethyltrideca-2,6,10-trienoic acid | | [M-H]- | | | Lipids and lipid-like molecules |  |
| 329 | 679.36510 | 194.5130 | | (2r,3r,4r,6ar,6bs,8as,11r,12r,12as,14br)-2,3,12-trihydroxy-4,6a,6b,11,12,14b-hexamethyl-8a-[(2s,3r,4s,5s,6r)-3,4,5-trihydroxy-6-(hydroxymethyl)oxan-2-yl]oxycarbonyl-1,2,3,4a,5,6,7,8,9,10,11,12a,14,14a-tetradecahydropicene-4-carboxylic acid | | [M-H]- | | | Lipids and lipid-like molecules |  |
| 330 | 485.13728 | 45.6625 | | (2s,3r,4as,12br)-2,3,4a,8-tetrahydroxy-12b-(5-hydroxy-6-methyloxan-2-yl)oxy-3-methyl-2,4-dihydrobenzo[a]anthracene-1,7,12-trione | | [M-H]- | | | Benzenoids |  |
| 331 | 265.10770 | 41.4740 | | (e)-5-(4-methoxy-5-methyl-6-oxopyran-2-yl)-3-methylhex-4-enoic acid | | [M-H]- | | | Lipids and lipid-like molecules |  |
| 332 | 349.06215 | 172.3390 | | (phenoxymethyl)penicilloic acid | | [M-H-H2O]- | | | Organic acids and derivatives |  |
| 333 | 299.25812 | 39.7440 | | (r)-2-hydroxystearic acid | | [M-H]- | | | Lipids and lipid-like molecules |  |
| 334 | 445.18538 | 41.2405 | | (z)-2,6-dimethyl-7-(4-methyl-5-oxooxolan-2-yl)-3-[[3,4,5-trihydroxy-6-(hydroxymethyl)oxan-2-yl]oxymethyl]hept-5-enoic acid | | [M-H]- | | | Undefined |  |
| 335 | 329.23166 | 163.8150 | | (z)-5,8,11-trihydroxyoctadec-9-enoic acid | | [M-H]- | | | Lipids and lipid-like molecules |  |
| 336 | 293.17489 | 68.0225 | | [6]-gingerol | | [M-H]- | | | Benzenoids |  |
| 337 | 495.27070 | 159.6230 | | 1-(10z-heptadecenoyl)-sn-glycero-3-phospho-(1'-rac-glycerol) | | [M-H]- | | | Undefined |  |
| 338 | 728.53837 | 154.4020 | | 1-(1z-octadecenyl)-2-(9z-octadecenoyl)-sn-glycero-3-phosphoethanolamine | | [M-H]- | | | Lipids and lipid-like molecules |  |
| 339 | 624.42842 | 156.7680 | | 1-behenoyl-2-hydroxy-sn-glycero-3-phosphocholine | | [M+HCO2]- | | | Lipids and lipid-like molecules |  |
| 340 | 791.49978 | 73.7555 | | 1-hexadecyl lysophosphatidic acid | | [2M-H]- | | | Lipids and lipid-like molecules |  |
| 341 | 642.43827 | 30.7850 | | 1-lignoceroyl-2-hydroxy-sn-glycero-3-phosphocholine | | [M+Cl]- | | | Lipids and lipid-like molecules |  |
| 342 | 750.50722 | 151.6875 | | 1-myristoyl-2-palmitoyl-sn-glycero-3-phosphocholine | | [M+HCO2]- | | | Lipids and lipid-like molecules |  |
| 343 | 403.26245 | 35.3790 | | 1-o-(9z-octadecenyl)-sn-glycero-2,3-cyclic-phosphate | | [M-H]- | | | Undefined |  |
| 344 | 509.28636 | 158.5085 | | 1-oleoyl-2-hydroxy-sn-glycero-3-phospho-(1'-rac-glycerol) | | [M-H]- | | | Lipids and lipid-like molecules |  |
| 345 | 478.29123 | 205.5520 | | 1-oleoyl-sn-glycero-3-phosphoethanolamine | | [M-H]- | | | Lipids and lipid-like molecules |  |
| 346 | 483.27039 | 161.1880 | | 1-palmitoyl-2-hydroxy-sn-glycero-3-phospho-(1'-rac-glycerol) | | [M-H]- | | | Lipids and lipid-like molecules |  |
| 347 | 452.27669 | 208.7640 | | 1-palmitoyl-2-hydroxy-sn-glycero-3-phosphoethanolamine | | [M-H]- | | | Lipids and lipid-like molecules |  |
| 348 | 712.49082 | 155.6300 | | 1-palmitoyl-2-lauroyl-sn-glycero-3-phosphorylcholine | | [M+Cl]- | | | Lipids and lipid-like molecules |  |
| 349 | 745.50025 | 38.1440 | | 1-palmitoyl-2-linoleoyl-sn-glycero-3-phospho-(1'-rac-glycerol) | | [M-H]- | | | Lipids and lipid-like molecules |  |
| 350 | 747.51818 | 37.5620 | | 1-palmitoyl-2-oleoyl-phosphatidylglycerol | | [M-H]- | | | Lipids and lipid-like molecules |  |
| 351 | 716.52221 | 156.1280 | | 1-palmitoyl-3-oleoyl-sn-glycero-2-phosphoethanolamine | | [M-H]- | | | Lipids and lipid-like molecules |  |
| 352 | 699.47678 | 34.0220 | | 1,2-dioleoyl-sn-glycero-3-phosphate | | [M-H]- | | | Lipids and lipid-like molecules |  |
| 353 | 756.55376 | 137.8200 | | 1,2-dioleoyl-sn-glycero-3-phosphoethanolamine-n-methyl | | [M-H]- | | | Lipids and lipid-like molecules |  |
| 354 | 686.47509 | 156.8550 | | 1,2-dipalmitoleoyl-sn-glycero-3-phosphoethanolamine | | [M-H]- | | | Lipids and lipid-like molecules |  |
| 355 | 721.49836 | 38.2420 | | 1,2-dipalmitoyl-sn-glycero-3-phospho-(1'-rac-glycerol) | | [M-H]- | | | Lipids and lipid-like molecules |  |
| 356 | 773.53302 | 76.9170 | | 1,2-distearoyl-sn-glycero-3-phospho-l-serine | | [M-H-NH3]- | | | Lipids and lipid-like molecules |  |
| 357 | 317.08074 | 46.9250 | | 1,4-d-xylobiose | | [M+Cl]- | | | Organic oxygen compounds |  |
| 358 | 481.29144 | 167.4360 | | 1.alpha.-methyl-5.alpha.-androstan-3.alpha.,17.beta.-diol glucuronide | | [M-H]- | | | Undefined |  |
| 359 | 513.30793 | 31.4775 | | 11-ketofusidic acid | | [M-H]- | | | Lipids and lipid-like molecules |  |
| 360 | 327.28870 | 57.3600 | | 12-hete-[d8] | | [M-H]1- | | | Lipids and lipid-like molecules |  |
| 361 | 365.24395 | 36.6660 | | 15-cyclohexylpentanorprostaglandin f2.alpha. | | [M-H]- | | | Lipids and lipid-like molecules |  |
| 362 | 339.19934 | 30.1060 | | 15-ketoiloprost | | [M-H-H2O]- | | | Lipids and lipid-like molecules |  |
| 363 | 371.25378 | 67.2630 | | 15(r),19(r)-hydroxyprostaglandin f1.alpha. | | [M-H]- | | | Lipids and lipid-like molecules |  |
| 364 | 271.22647 | 40.5385 | | 16-hydroxyhexadecanoic acid | | [M-H]- | | | Lipids and lipid-like molecules |  |
| 365 | 363.25387 | 51.9040 | | 16,16-dimethylprostaglandin a1 | | [M-H]- | | | Lipids and lipid-like molecules |  |
| 366 | 495.43943 | 96.4180 | | 16,19,22,25,28,31-tetratriacontahexaenoic acid, (all-z)- | | [M-H]- | | | Lipids and lipid-like molecules |  |
| 367 | 337.18400 | 30.7375 | | 18-carboxydinorleukotriene b4 | | [M-H]- | | | Lipids and lipid-like molecules |  |
| 368 | 337.23542 | 53.7340 | | 2-(2-hydroxybut-3-en-2-yl)-3a,6,6,9a-tetramethyl-2,4,5,5a,7,8,9,9b-octahydro-1h-benzo[e][1]benzofuran-4,5-diol | | [M-H]- | | | Undefined |  |
| 369 | 343.13845 | 83.9250 | | 2-[1-hydroxy-1-(4-methoxyphenyl)propan-2-yl]oxy-6-(hydroxymethyl)oxane-3,4,5-triol | | [M-H]- | | | Organic oxygen compounds |  |
| 370 | 527.25295 | 171.5000 | | 2-cis-4-trans-abscisic acid | | [2M-H]- | | | Lipids and lipid-like molecules |  |
| 371 | 223.02699 | 34.6510 | | 2-hydroxyanthraquinone | | [M-H]- | | | Benzenoids |  |
| 372 | 243.19547 | 78.1990 | | 2-hydroxymyristic acid | | [M-H]- | | | Lipids and lipid-like molecules |  |
| 373 | 714.50788 | 155.5215 | | 2-linoleoyl-1-palmitoyl-sn-glycero-3-phosphoethanolamine | | [M-H]- | | | Lipids and lipid-like molecules |  |
| 374 | 141.01670 | 308.3095 | | 2-Oxoadipic acid | | (M-H2O-H)- | | | Organic acids and derivatives |  |
| 375 | 213.05509 | 54.7450 | | 2,4-dihydroxybenzophenone | | [M-H]- | | | Benzenoids |  |
| 376 | 555.28339 | 167.4360 | | 2,5,7,8-tetramethyl-2-(.beta.-carboxyethyl)-6-hydroxychroman | | [2M-H]- | | | Organoheterocyclic compounds |  |
| 377 | 220.14721 | 713.2605 | | 2,6-di-tert-butyl-4-methoxyphenol | | [M-H-CH3]- | | | Benzenoids |  |
| 378 | 785.45070 | 73.3420 | | 3-(1h-indol-3-ylmethyl)-6,18-dimethyl-9-(2-methylpropyl)-12-(1-phenylethyl)-15-propan-2-yl-1,4,7,10,13,16,19-heptazacyclotricosane-2,5,8,11,14,17,20-heptone | | [M-H]- | | | Organic acids and derivatives |  |
| 379 | 255.06545 | 49.2780 | | 3-(5-phenylthiophen-2-yl)prop-2-ynyl acetate | | [M-H]- | | | Organoheterocyclic compounds |  |
| 380 | 236.09546 | 32.6810 | | 3-(cyclohexylamino)-2-hydroxy-1-propanesulfonic acid | | [M-H]- | | | Organic nitrogen compounds |  |
| 381 | 957.53288 | 164.9290 | | 3-glc-gal-glcua-soyasapogenol b | | [M-H]- | | | Lipids and lipid-like molecules |  |
| 382 | 181.00847 | 379.5490 | | 3,4-dihydroxyhydrocinnamic acid | | [M-H]- | | | Undefined |  |
| 383 | 659.06833 | 456.0550 | | 3',5'-cyclic inosine monophosphate | | [2M-H]- | | | Nucleosides, nucleotides, and analogues |  |
| 384 | 408.28596 | 53.4700 | | 5,8,11,14-eicosatetraenamide, n-(4-hydroxy-2-methylphenyl)-, (5z,8z,11z,14z)- | | [M-H]- | | | Benzenoids |  |
| 385 | 257.21209 | 69.1190 | | 5,8,11,14-eicosatetraenoic acid, 16-hydroxy-, (5z,8z,11z,14z,16r)- | | [M-H-CH2O3]- | | | Lipids and lipid-like molecules |  |
| 386 | 449.24159 | 157.9670 | | 5.alpha.-androstan-3.alpha.,17.beta.-diol-o-3-.beta.-glucuronic acid | | [M-H-H2O]- | | | Lipids and lipid-like molecules |  |
| 387 | 395.24274 | 34.9290 | | 5(S)-HpETE | | (M+CH3COO)- | | | Undefined |  |
| 388 | 426.96509 | 28.9340 | | 6:2 fluorotelomer sulfonic acid | | [M-H]- | | | Organic acids and derivatives |  |
| 389 | 257.92358 | 168.5855 | | 7-bromo-5-chloroquinolin-8-ol | | [M-H+2i]- | | | Organoheterocyclic compounds |  |
| 390 | 495.30655 | 164.0325 | | 7.alpha.,17.alpha.-dimethyl-5.beta.-androstane-3.alpha.,17.beta.-diol glucuronide | | [M-H]- | | | Lipids and lipid-like molecules |  |
| 391 | 221.15315 | 34.6920 | | 8,11-tridecadienoic acid, 13-(3-pentyl-2-oxiranyl)-, (8z,11z)- | | [M-H-C6H12O]- | | | Lipids and lipid-like molecules |  |
| 392 | 299.20025 | 52.2630 | | 8(9)-epoxy-5z,11z,14z,17z-eicosatetraenoic acid | | [M-H-H2O]- | | | Lipids and lipid-like molecules |  |
| 393 | 473.27928 | 29.4650 | | Amastatin | | [M-H]- | | | Organic acids and derivatives |  |
| 394 | 409.10693 | 51.5990 | | Amorphigenin | | [M-H]- | | | Phenylpropanoids and polyketides |  |
| 395 | 265.18020 | 62.3590 | | Aphyllic acid | | [M-H]- | | | Organic acids and derivatives |  |
| 396 | 523.30238 | 156.8180 | | Asiatic acid | | [M+Cl]- | | | Lipids and lipid-like molecules |  |
| 397 | 187.09679 | 355.4470 | | Azelaic acid | | [M-H]- | | | Lipids and lipid-like molecules |  |
| 398 | 339.32536 | 38.1795 | | Behenic acid | | [M-H]- | | | Lipids and lipid-like molecules |  |
| 399 | 227.12800 | 95.6315 | | Bisphenol a | | [M-H]- | | | Benzenoids |  |
| 400 | 331.09612 | 46.9435 | | Butamifos | | [M-H]- | | | Benzenoids |  |
| 401 | 171.13809 | 58.3335 | | Capric acid | | [M-H]- | | | Lipids and lipid-like molecules |  |
| 402 | 451.30406 | 164.0760 | | Chenodeoxycholate | | (M+CH3COO)- | | | Lipids and lipid-like molecules |  |
| 403 | 605.15932 | 50.9110 | | Chlorambucil | | [2M-H]- | | | Organic nitrogen compounds |  |
| 404 | 465.30368 | 28.8340 | | Cholesteryl sulfate | | [M-H]- | | | Lipids and lipid-like molecules |  |
| 405 | 407.27905 | 231.9815 | | Cholic acid | | [M-H]- | | | Lipids and lipid-like molecules |  |
| 406 | 293.17958 | 28.3080 | | Cinchonine | | [M-H]- | | | Undefined |  |
| 407 | 331.26348 | 48.9730 | | Cis-7,10,13,16-docosatetraenoic acid | | [M-H]- | | | Lipids and lipid-like molecules |  |
| 408 | 253.21675 | 52.7790 | | Cis-9-palmitoleic acid | | [M-H]- | | | Lipids and lipid-like molecules |  |
| 409 | 141.01621 | 375.6135 | | Cis,cis-muconic acid | | [M-H]- | | | Lipids and lipid-like molecules |  |
| 410 | 746.45049 | 158.4100 | | Clarithromycin | | [M-H]- | | | Organic oxygen compounds |  |
| 411 | 531.29806 | 121.4530 | | Cochlioquinone a | | [M-H]- | | | Undefined |  |
| 412 | 637.36028 | 159.6725 | | Decahydrogambogic acid | | [M-H]- | | | Organoheterocyclic compounds |  |
| 413 | 391.28397 | 163.0485 | | Deoxycholic acid | | [M-H]- | | | Lipids and lipid-like molecules |  |
| 414 | 867.02171 | 456.0840 | | Diguanosine tetraphosphate | | [M-H]- | | | Nucleosides, nucleotides, and analogues |  |
| 415 | 199.17056 | 56.0530 | | Dodecanoic acid | | [M-H]- | | | Lipids and lipid-like molecules |  |
| 416 | 531.30050 | 28.8150 | | Dodecyl sulfate | | [2M-H]- | | | Organic acids and derivatives |  |
| 417 | 471.34786 | 57.3960 | | Echinocystic acid | | [M-H]- | | | Lipids and lipid-like molecules |  |
| 418 | 309.27907 | 50.2700 | | Eicosenoic acid | | [M-H]- | | | Lipids and lipid-like molecules |  |
| 419 | 337.20429 | 28.3080 | | Eplerenone hydroxy acid | | [M-H-C2H6O4]- | | | Undefined |  |
| 420 | 329.23150 | 122.0570 | | Fa 18:1+3o | | [M-H]- | | | Lipids and lipid-like molecules |  |
| 421 | 665.07113 | 453.5940 | | Fenoxaprop | | [2M-H]- | | | Benzenoids |  |
| 422 | 689.37393 | 59.2965 | | Fumonisin la4 | | [M-H]- | | | Organic acids and derivatives |  |
| 423 | 703.35198 | 40.5400 | | Fumonisin py2 | | [M-H]- | | | Organic acids and derivatives |  |
| 424 | 687.35986 | 60.5885 | | Fumonisin py4 | | [M-H]- | | | Organic acids and derivatives |  |
| 425 | 651.37568 | 158.7220 | | Fungichromin | | (M-H2O-H)- | | | Undefined |  |
| 426 | 401.12787 | 379.1520 | | Galactinol | | (M+CH3COO)- | | | Organic oxygen compounds |  |
| 427 | 553.26645 | 168.7810 | | Ganoderic acid h | | [M-H-H2O]- | | | Lipids and lipid-like molecules |  |
| 428 | 269.13879 | 110.7290 | | Genistein | | M-H | | | Phenylpropanoids and polyketides |  |
| 429 | 945.53139 | 165.2975 | | Ginsenoside rd | | [M-H]- | | | Lipids and lipid-like molecules |  |
| 430 | 765.48167 | 76.7860 | | Ginsenoside rg5 | | [M-H]- | | | Lipids and lipid-like molecules |  |
| 431 | 811.46697 | 71.1580 | | Ginsenoside rg6 | | [M+FA-H]- | | | Lipids and lipid-like molecules |  |
| 432 | 464.31203 | 208.5840 | | Glycocholic acid | | [M-H]- | | | Lipids and lipid-like molecules |  |
| 433 | 325.30939 | 39.4940 | | Heneicosanoic acid | | [M-H]- | | | Lipids and lipid-like molecules |  |
| 434 | 269.24710 | 50.2990 | | Heptadecanoic acid | | [M-H]- | | | Lipids and lipid-like molecules |  |
| 435 | 395.38781 | 47.2815 | | Hexacosanoic acid | | [M-H]- | | | Lipids and lipid-like molecules |  |
| 436 | 241.08209 | 105.4920 | | His-ser | | [M-H]- | | | Nucleosides, nucleotides, and analogues |  |
| 437 | 325.18459 | 30.7270 | | Hydroquinidine | | [M-H]- | | | Undefined |  |
| 438 | 323.16700 | 31.3740 | | Hymeglusin | | [M-H]- | | | Lipids and lipid-like molecules |  |
| 439 | 227.06701 | 121.8850 | | Ile-Pro | | [M-H]- | | | Nucleosides, nucleotides, and analogues |  |
| 440 | 707.50072 | 83.9250 | | Ionomycin | | [M-H]- | | | Lipids and lipid-like molecules |  |
| 441 | 485.27772 | 31.9160 | | Kendomycin | | [M-H]- | | | Undefined |  |
| 442 | 529.30373 | 33.4850 | | Kukoamine a | | [M-H]- | | | Benzenoids |  |
| 443 | 381.23012 | 29.4700 | | L-alaninamide, n-[2-[2-(hydroxyamino)-2-oxoethyl]-4-methyl-1-oxopentyl]-3-methyl-l-valyl-n-(2-aminoethyl)- | | [M-H-H3NO]- | | | Organic acids and derivatives |  |
| 444 | 425.36107 | 113.4220 | | Lanosterol | | [M-H]- | | | Lipids and lipid-like molecules |  |
| 445 | 539.33312 | 149.1750 | | Leptomycin b | | [M-H]- | | | Lipids and lipid-like molecules |  |
| 446 | 567.28139 | 166.2670 | | Leukotriene f4 | | [M-H]- | | | Lipids and lipid-like molecules |  |
| 447 | 367.35628 | 37.9720 | | Lignoceric acid | | [M-H]- | | | Lipids and lipid-like molecules |  |
| 448 | 279.23268 | 50.8975 | | Linoleic acid | | [M-H]- | | | Lipids and lipid-like molecules |  |
| 449 | 269.21123 | 39.4740 | | Loxistatin acid | | [M-H-CO2]- | | | Organic acids and derivatives |  |
| 450 | 413.26605 | 129.5550 | | Lupulone | | [M-H]- | | | Undefined |  |
| 451 | 341.07579 | 83.9400 | | Maltose | | [M-H]- | | | Organic oxygen compounds |  |
| 452 | 661.34547 | 61.3830 | | Medicagenic acid base -h2o + o-hexa | | [M-H]- | | | Lipids and lipid-like molecules |  |
| 453 | 275.16796 | 34.0040 | | Menthyl salicylate | | [M-H]- | | | Benzenoids |  |
| 454 | 309.17367 | 28.8440 | | Mestranol | | [M-H]- | | | Lipids and lipid-like molecules |  |
| 455 | 227.20085 | 54.0950 | | Myristic acid | | [M-H]- | | | Lipids and lipid-like molecules |  |
| 456 | 394.26324 | 53.5030 | | N-(3-hydroxyphenyl)arachidonoylamide | | [M-H]- | | | Benzenoids |  |
| 457 | 765.56759 | 116.4330 | | N-(octadecanoyl)sphing-4-enine-1-phosphocholine | | [M+Cl]- | | | Lipids and lipid-like molecules |  |
| 458 | 374.24536 | 37.9565 | | N-arachidonoyl-l-alanine | | [M-H]- | | | Organic acids and derivatives |  |
| 459 | 438.29748 | 211.7390 | | N-arachidonoyldopamine | | [M-H]- | | | Benzenoids |  |
| 460 | 645.49763 | 195.1160 | | N-lauroyl-d-erythro-sphingosylphosphorylcholine | | [M-H]- | | | Lipids and lipid-like molecules |  |
| 461 | 618.47641 | 36.8750 | | N-palmitoyl-d-erythro-dihydroceramide-1-phosphate | | [M-H]- | | | Lipids and lipid-like molecules |  |
| 462 | 663.07293 | 456.0510 | | Nicotinic acid adenine dinucleotide | | [M-H]- | | | Nucleosides, nucleotides, and analogues |  |
| 463 | 157.12308 | 60.6300 | | Nonanoic acid | | [M-H]- | | | Lipids and lipid-like molecules |  |
| 464 | 423.41861 | 46.6815 | | Octacosanoic acid | | [M-H]- | | | Lipids and lipid-like molecules |  |
| 465 | 283.26556 | 38.9220 | | Octadecanoic acid | | [M-H]- | | | Lipids and lipid-like molecules |  |
| 466 | 455.34939 | 36.5240 | | Oleanolic acid | | [M-H]- | | | Lipids and lipid-like molecules |  |
| 467 | 803.49850 | 72.7310 | | Oligomycin b | | [M-H]- | | | Undefined |  |
| 468 | 292.18845 | 110.6770 | | Olopatadine | | [M-H-CO2]- | | | Organoheterocyclic compounds |  |
| 469 | 297.15326 | 31.3775 | | Ostruthin | | [M-H]- | | | Lipids and lipid-like molecules |  |
| 470 | 255.23421 | 51.6230 | | Palmitic acid | | [M-H]- | | | Lipids and lipid-like molecules |  |
| 471 | 804.57258 | 153.5180 | | Pc 33:1 | | [M+CH3COOH-H]- | | | Undefined |  |
| 472 | 836.54292 | 149.4870 | | Pc 36:6 | | [M+CH3COOH-H]- | | | Lipids and lipid-like molecules |  |
| 473 | 886.55801 | 145.5570 | | Pc 40:9 | | [M+CH3COOH-H]- | | | Lipids and lipid-like molecules |  |
| 474 | 732.55588 | 139.0770 | | PC(16:0/16:0) | | [M-H]- | | | Lipids and lipid-like molecules |  |
| 475 | 818.59168 | 152.3780 | | Pc(16:0e/13-hode) | | [M+Hac-H]- | | | Lipids and lipid-like molecules |  |
| 476 | 864.57285 | 147.9320 | | Pc(16:1e/17-hdohe) | | [M+Hac-H]- | | | Lipids and lipid-like molecules |  |
| 477 | 838.55944 | 149.0880 | | Pc(16:1e/8,9-epete) | | [M+Hac-H]- | | | Lipids and lipid-like molecules |  |
| 478 | 816.57596 | 151.9110 | | Pc(16:1e/9-hode) | | [M+Hac-H]- | | | Lipids and lipid-like molecules |  |
| 479 | 866.59835 | 147.5140 | | Pc(18:1e/8-hepe) | | [M+Hac-H]- | | | Lipids and lipid-like molecules |  |
| 480 | 844.60425 | 151.0950 | | Pc(18:1e/9-hode) | | [M+Hac-H]- | | | Lipids and lipid-like molecules |  |
| 481 | 688.49148 | 156.8550 | | Pe 32:1 | | [M-H]- | | | Lipids and lipid-like molecules |  |
| 482 | 764.52096 | 150.1370 | | Pe(16:0e/10-hdohe) | | [M-H]- | | | Lipids and lipid-like molecules |  |
| 483 | 740.52216 | 153.6240 | | Pe(16:0e/12-hete) | | [M-H]- | | | Lipids and lipid-like molecules |  |
| 484 | 762.50664 | 150.6325 | | Pe(16:1e/7-hdohe) | | [M-H]- | | | Lipids and lipid-like molecules |  |
| 485 | 241.21622 | 53.4630 | | Pentadecanoic acid | | [M-H]- | | | Lipids and lipid-like molecules |  |
| 486 | 1175.77609 | 32.6840 | | Pentaerythritol tetrakis(3,5-di-tert-butyl-4-hydroxyhydrocinnamate) | | [M-H]- | | | Organic acids and derivatives |  |
| 487 | 684.46210 | 54.7290 | | Pepstatin a | | [M-H]- | | | Organic acids and derivatives |  |
| 488 | 327.25208 | 58.4750 | | Phenol, 5-(1,1-dimethylnonyl)-2-(3-hydroxycyclohexyl)-, cis- | | [M-H-H2O]- | | | Benzenoids |  |
| 489 | 313.27350 | 59.9045 | | Phenol, 5-(1,1-dimethyloctyl)-2-[(1s,3s)-3-hydroxycyclohexyl]- | | [M-H-H2O]- | | | Benzenoids |  |
| 490 | 273.03324 | 115.2145 | | Phenylbenzimidazolesulfonic acid | | [M-H]- | | | Organoheterocyclic compounds |  |
| 491 | 283.11864 | 83.9920 | | Phenylethyl 2-glucoside | | [M-H]- | | | Organic oxygen compounds |  |
| 492 | 540.36624 | 205.9460 | | Phosphatidylcholine lyso alkyl 16:0 | | [M+CH3COO]- | | | Lipids and lipid-like molecules |  |
| 493 | 396.21722 | 34.0300 | | Phosphonic acid, p-[[4-[(1-oxotetradecyl)amino]phenyl]methyl]- | | [M-H]- | | | Benzenoids |  |
| 494 | 283.06772 | 22.4220 | | Physcion | | [M-H]- | | | Benzenoids |  |
| 495 | 311.29429 | 37.9720 | | Phytanic acid | | [M-H]- | | | Lipids and lipid-like molecules |  |
| 496 | 913.58139 | 33.4930 | | Pi 40:4 | | [M-H]- | | | Lipids and lipid-like molecules |  |
| 497 | 899.54368 | 168.0460 | | Pi(18:0/12-hepe) | | [M-H]- | | | Lipids and lipid-like molecules |  |
| 498 | 897.53182 | 168.8180 | | Pi(18:1/15-hepe) | | [M-H]- | | | Lipids and lipid-like molecules |  |
| 499 | 497.32194 | 163.6390 | | Poricoic acid a | | [M-H]- | | | Lipids and lipid-like molecules |  |
| 500 | 311.18517 | 74.6120 | | Pregna-4,16-diene-3,20-dione | | [M-H]- | | | Lipids and lipid-like molecules |  |
| 501 | 501.31334 | 127.6515 | | Propanoic acid, 2-[[4-[2-[[(cyclohexylamino)carbonyl](4-cyclohexylbutyl)amino]ethyl]phenyl]thio]-2-methyl- | | [M-H]- | | | Organosulfur compounds |  |
| 502 | 335.22114 | 58.0530 | | Prostaglandin d1 | | [M-H-H2O]- | | | Lipids and lipid-like molecules |  |
| 503 | 707.48612 | 84.1360 | | Prostaglandin e1 | | [2M-H]- | | | Lipids and lipid-like molecules |  |
| 504 | 367.21518 | 63.1430 | | Prostaglandin g2 | | [M-H]- | | | Lipids and lipid-like molecules |  |
| 505 | 351.22041 | 39.8685 | | Prostaglandin i2 | | [M-H]- | | | Lipids and lipid-like molecules |  |
| 506 | 264.15770 | 127.8780 | | Pyroxamide | | [M-H]- | | | Undefined |  |
| 507 | 353.19827 | 29.4895 | | Rauwolscine | | [M-H]- | | | Undefined |  |
| 508 | 343.04462 | 404.5610 | | Rhein | | (M+CH3COO)- | | | Undefined |  |
| 509 | 297.24217 | 57.9490 | | Ricinoleic acid | | [M-H]- | | | Lipids and lipid-like molecules |  |
| 510 | 439.37813 | 110.2610 | | Roburic acid | | [M-H]- | | | Lipids and lipid-like molecules |  |
| 511 | 695.30878 | 33.7445 | | S4:18(p3:16/f1:2) | | [M+HCOO]- | | | Undefined |  |
| 512 | 567.53244 | 51.5610 | | Stearic acid | | (2M-H)- | | | Lipids and lipid-like molecules |  |
| 513 | 341.10705 | 378.3975 | | Sucrose | | [M-H]- | | | Organic oxygen compounds |  |
| 514 | 574.45095 | 34.3670 | | Sulfobacin b | | [M-H]- | | | Lipids and lipid-like molecules |  |
| 515 | 296.06386 | 77.9720 | | Sulindac | | [M-H-C2H3O2]- | | | Benzenoids |  |
| 516 | 471.36113 | 60.6785 | | Sumaresinolic acid | | [M-H]- | | | Lipids and lipid-like molecules |  |
| 517 | 498.28873 | 149.1750 | | Taurochenodeoxycholate | | [M-H]- | | | Lipids and lipid-like molecules |  |
| 518 | 514.28347 | 202.8110 | | Taurocholate | | [M-H]- | | | Lipids and lipid-like molecules |  |
| 519 | 287.22147 | 113.3345 | | Testosterone | | [M-H]- | | | Lipids and lipid-like molecules |  |
| 520 | 311.16832 | 30.7310 | | Thymol-beta-d-glucoside | | [M-H]- | | | Lipids and lipid-like molecules |  |
| 521 | 324.21716 | 123.3615 | | Tolterodine | | [M-H]- | | | Benzenoids |  |
| 522 | 285.20642 | 73.5740 | | Totarol | | [M-H]- | | | Lipids and lipid-like molecules |  |
| 523 | 353.34023 | 37.9800 | | Tricosanoic acid | | [M-H]- | | | Lipids and lipid-like molecules |  |
| 524 | 215.12775 | 321.3000 | | Undecanedioic acid | | [M-H]- | | | Lipids and lipid-like molecules |  |
| 525 | 243.06123 | 171.5410 | | Uridine | | [M-H]- | | | Nucleosides, nucleotides, and analogues |  |
| 526 | 449.32888 | 28.8550 | | Vitamin k1 | | [M-H]- | | | Lipids and lipid-like molecules |  |
| 527 | 431.08673 | 51.2350 | | Vitexin | | [M-H]- | | | Phenylpropanoids and polyketides |  |
| 528 | 265.14937 | 30.0940 | | Zinniol | | [M-H]- | | | Benzenoids |  |
